# Adherence to PCSK9 Inhibitors in Clinical Practice: Systematic Review and Meta-Analysis of Observational Studies

**DOI:** 10.1016/j.jacadv.2026.102733

**Published:** 2026-04-17

**Authors:** Joseph Edgar Blais, Wenfang Zhong, Cheuk Ying Vanessa Li, Chuenjid Kongkaew, Amy Hai Yan Chan

**Affiliations:** a Centre for Safe Medication Practice and Research, Department of Pharmacology and Pharmacy, Li Ka Shing Faculty of Medicine, The University of Hong Kong, Pokfulam, Hong Kong Special Administrative Region, China; b Centre for Safety and Quality in Health, Department of Pharmacy Practice, Faculty of Pharmaceutical Sciences, Naresuan University, Phitsanulok, Thailand; c School of Pharmacy, Faculty of Medical and Health Sciences, The University of Auckland, Auckland, New Zealand

**Keywords:** alirocumab, discontinuation, evolocumab, inclisiran, initiation, medication adherence

## Abstract

**Background:**

Medication nonadherence is a barrier to the long-term effectiveness of proprotein convertase subtilisin/kexin type 9 (PCSK9) inhibitors in clinical practice.

**Objectives:**

The aim of the study was to determine the prevalence of adherence to PCSK9 inhibitors in real-world practice across all adherence phases.

**Methods:**

MEDLINE, EMBASE, PsycINFO, CINAHL Plus, and medRxiv were searched from inception to August 2, 2024. Observational studies reporting at least 1 quantitative adherence measure for alirocumab, evolocumab, or inclisiran were included. Study quality was assessed with the Joanna Briggs Institute checklist for prevalence studies. Data were pooled using random-effects meta-analysis with multilevel models to account for measurements at multiple time points. Measures of medication adherence were categorized into initiation, implementation (medication possession ratio [MPR] and proportion of days covered), and persistence (persistence and discontinuation) phases.

**Results:**

We included 94 studies in the systematic review, with 56 studies (n = 75,902), primarily evaluating alirocumab and evolocumab, contributing to a quantitative synthesis. Initiation was high at 91.7% (95% CI: 83.6-96.0; I^2^ = 94.2%). MPR was 95.1% (95% CI: 92.7-97.5; I^2^ = 98.4%) at 6 months and 86.5% (95% CI: 80.2-92.9; I^2^ = 99.7%) at 24 months. The 12-month proportion of days covered was 69.7% (95% CI: 55.9-83.5; I^2^ = 99.8%). At 12 months, persistence was 81.8% (95% CI: 68.2-90.4; I^2^ = 99.1%), and the discontinuation rate was 12.1% (95% CI: 7.4-19.0; I^2^ = 98.9%). Multilevel meta-analyses demonstrated declines in MPR and persistence beyond 12 months of follow-up.

**Conclusions:**

Although patients frequently initiate PCSK9 inhibitors, both implementation and persistence diminish over time, underscoring the need for strategies that sustain medication adherence in clinical practice. With limited evidence beyond 24 months of follow-up and for inclisiran, additional long-term observational studies are essential to guide real-world management.

Proprotein convertase subtilisin/kexin type 9 (PCSK9) inhibitors profoundly reduce low-density lipoprotein (LDL) cholesterol levels and lower cardiovascular risk.^1^ Currently available PCSK9 inhibitors are administered via injection and are typically intended for lifelong use.^2^ First-generation PCSK9 inhibitors, monoclonal antibodies (moAbs) such as alirocumab and evolocumab, are self-administered every 2 to 4 weeks. The second-generation PCSK9 inhibitor, inclisiran, is a small interfering ribonucleic acid (siRNA) administered by health care professionals biannually during the maintenance phase.^2^

Despite the efficacy of PCSK9 inhibitors in randomized controlled trials (RCTs), emerging real-world evidence indicates important medication adherence challenges. Medication adherence, defined as the process by which patients follow prescribed treatment regimens, is a dynamic behavior categorized into 3 phases based on the adherence taxonomy by Vrijens: initiation, implementation, and persistence.^3^^,^^4^ Studies of PCSK9 inhibitor moAbs have reported first-year discontinuation rates approaching 50%, which can lead to suboptimal LDL cholesterol reduction compromising prevention of cardiovascular events.^5^, ^6^, ^7^, ^8^, ^9^, ^10^, ^11^, ^12^, ^13^ While inclisiran’s twice-yearly dosing regimen is hypothesized to enhance medication adherence,^14^ real-world data remain limited and inconclusive regarding whether inclisiran yields better adherence than moAbs.^15^^,^^16^ Understanding adherence patterns across each medication adherence phase is therefore critical for identifying barriers and developing strategies to improve long-term adherence to PCSK9 inhibitors.

Although individual studies have reported on medication adherence to PCSK9 inhibitors in real-world settings, no systematic synthesis has been conducted assessing the prevalence of nonadherence across the different phases of the medication adherence process. Our systematic review aimed to synthesize quantitative evidence on short- and long-term medication adherence among individuals prescribed a PCSK9 inhibitor from observational studies across all adherence phases and to examine variations in medication adherence by geographical region, data source, and PCSK9 inhibitor drug class.

## Methods

Review protocol was registered on PROSPERO CRD42024567957) prior to conducting the initial literature search. This study-level systematic review was managed in Covidence (Veritas Health Innovation) and reported following the Preferred Reporting Items for Systematic Reviews and Meta-Analyses 2020 recommendations (Supplemental Table 1).^17^ Ethical approval was not required as this study is a systematic review of primary research.

### Literature search

We searched for published and preprint reports using MEDLINE (OVID), EMBASE (OVID), PsychInfo (ProQuest), CINAHL Plus (EBSCOHost), and medRxiv. Each literature database was searched from inception to 2 August 2024 to identify studies that reported on medication adherence to commercially available PCSK9 inhibitors (ie, alirocumab, evolocumab, or inclisiran). The literature search strategy was based on the defined patient, intervention, and outcome terms including “PCSK9 inhibitors,” “alirocumab,” “evolocumab,” “inclisiran,” “medication adherence,” “compliance,” “persistence,” and “discontinuation” and are provided in Supplemental Table 2.

### Eligibility criteria

We included observational studies with a cohort or cross-sectional design that assessed quantitative outcomes of medication adherence to PCSK9 inhibitor treatment. No restrictions were placed on the study data source or method of data collection (eg, registry, insurance claims data, electronic health records, chart reviews, and survey). Studies meeting the following criteria were included in the systematic review: reported at least 1 phase of adherence (initiation, implementation, or persistence); measured adherence as a quantitative outcome such as a proportion, time-to-event, or self-reported quantitative scale; assessed adherence to alirocumab, evolocumab, or inclisiran, either as monotherapy or in combination with other lipid-modifying medication; and had no restriction on the patient population (eg, age and indication for PCSK9 inhibitor). We included published studies, conference abstracts, and preprints in English.

We excluded studies that did not assess adherence to at least one of the PCSK9 inhibitors of interest (alirocumab, evolocumab, or inclisiran); RCTs, single-arm clinical trials, case reports, case series, or modeling studies; did not report medication adherence initiation, implementation, or persistence as a quantitative outcome or lacked sufficient information on adherence-related measures. RCTs were not included, as our objective was to evaluate adherence in real-world clinical practice rather than in trial participants, where adherence tends to be higher due to more controlled setting.

### Outcomes

To ensure consistency and comparability, we categorized the primary outcomes of interest—medication adherence measures—into 3 recommended adherence phases, following the ESPACOMP Medication Adherence Reporting Guideline (EMERGE),^3^^,^^4^ with an additional phase of reinitiation to account for temporary interruptions in PCSK9 inhibitor use. The phases and quantitative adherence measures were categorized as follows.

1. Initiation: the proportion of individuals prescribed a PCSK9 inhibitor who start treatment, assessed as a binary outcome at baseline.
2. Implementation or adherence: the medication possession ratio (MPR), calculated as the sum of the supply days of medication dispensed over a specified period divided by the number of days in that period; the proportion of days covered (PDC), defined as the proportion of days within a defined time window during which the patient was covered by a supply of the PCSK9 inhibitor; and adherence ≥80%, based on the proportion of patients with an MPR or PDC ≥80%.
3. Persistence: the proportion of persistent patients; the discontinuation rate and reasons; and the time from PCSK9 inhibitor initiation to permanent discontinuation.
4. Reinitiation: the resumption rate, defined as the proportion of patients who resumed PCSK9 inhibitor therapy following a temporary treatment interruption (as defined in original studies); switching rate, defined as the proportion of patients who switched between PCSK9 inhibitor moAb drugs.

The operational definitions of each adherence measure were as defined or reported in the original studies.

### Study selection

Search results were imported into Covidence review management software, where duplicates were automatically removed by the software. Two reviewers (V.C.Y.L., R.M., or J.E.B.) independently screened the titles and abstracts of study reports for potential relevance. Studies outside the scope of the review were excluded, and the full text of potentially eligible studies were independently assessed by 2 reviewers (V.C.Y.L. and J.E.B.), documenting the primary reason for exclusion based on a coded hierarchical list of exclusion criteria detailed in Supplemental Table 3A. Any discrepancies during abstract and full-text screening were resolved through discussion until consensus was reached. A list of studies excluded after full-text review and the reason for exclusion are provided in Supplemental Table 3B.

### Data extraction

One reviewer (V.C.Y.L. or W.Z.) extracted data on study characteristics, study data sources and setting, study participants, medication adherence definitions and outcomes (Supplemental Tables 4B and 4C) using a custom data extraction form in Covidence. A second reviewer (V.C.Y.L., W.Z., or J.E.B.) independently checked all extracted data for accuracy.

### Quality assessment

We selected the Joanna Briggs Institute (JBI) prevalence critical appraisal tool to assess study quality because of its coverage of key methodological domains and ease of use.^18^, ^19^, ^20^ This choice was based on the nature of medication adherence measures, which are typically calculated as a point or period prevalence. Although medication persistence is best conceptualized as a longitudinal time-to-event outcome, in practice it is often reported as the proportion of individuals who persist at a specific follow-up time making it challenging to apply checklists for cohort studies of interventions. Each checklist item was categorized as ‘yes,’ ‘no,’ or ‘unclear.’ Two reviewers independently conducted the quality assessment using the JBI tool, with any discrepancies resolved through discussion with a third reviewer. For each study, the maximum score that could be obtained on the JBI tool is 9, with each “Yes” response scoring 1 point, and both “No” and “Unclear” responses scoring 0. In line with previous studies, a study with a score of ≥6 was considered to be at low risk of bias.^21^^,^^22^

### Quantitative synthesis and data analysis

We conducted meta-analyses on the number of cohorts (indicated by a lowercase letter) or the number of observations to explore medication adherence at specific time points, as well as short-term (≤12 months) and long-term (>12 months). To ensure data completeness for quality assessment and methodological rigor, the meta-analyses were restricted to studies published as full journal articles. Given the anticipated statistical heterogeneity, consistent with previous adherence studies,^23^, ^24^, ^25^ we employed a random-effects model using a generalized linear mixed model for pooling studies.

The selection of specific time points for each adherence measure in our main analyses was based on whether the sample size was the largest. For MPR, we analyzed the pooled estimates at 6 and 24 months separately. Adherence measures that were pooled included PDC, proportion of adherence ≥80%, proportion of persistent patients, and discontinuation at 12 months. We also pooled the proportion of initiation, reinitiation, switching, discontinuation for different reasons, and the time from PCSK9 inhibitor initiation to permanent interruption.

When exploring short- and long-term medication adherence, a single study might contribute data for multiple time points within the same time period. To account for the dependency of estimates at multiple times within studies, we fit three-level meta-analytic models with time periods (short-term or long-term) as a moderator.^26^ The three-level model incorporates 3 distinct strata of effect size variability: the sampling variance associated with individual extracted effect sizes (level 1), the variance among effect sizes derived from the same study (level 2), and the variance between effect sizes obtained from different studies (level 3). The variability at levels 2 and 3 is estimated, whereas the variability at level 1 is presumed to be known and is calculated based on the observed sampling variance of the extracted effect sizes.^27^ An omnibus test based on the F-distribution was conducted to examine whether the moderating effect of time periods was significant.

For continuous variables, we standardized the sample mean and SD.^28^ Proportions were combined using logit transformation to address the issue of CI estimates falling outside the zero to 1 range.^29^ Time from PCSK9i initiation to permanent interruption was combined using log transformation. Statistical heterogeneity was assessed using *I*^*2*^. If a meta-analysis of a medication adherence measure had adequate power (ie, ≥10 cohorts), funnel plots were generated and visually inspected, and the Egger test was used to assess for small study effects (funnel plot asymmetry) potentially caused by reporting bias.^30^

### Subgroup and sensitivity analyses

We conducted subgroup analyses by stratifying studies according to geographical region (ie, United States, Europe, and other regions) and study data source (ie, patient support program, outpatient specialty clinics, database, survey, and multiple data sources). However, subgroup analysis to compare drug class (moAb and siRNA) and medication type (alirocumab, evolocumab, and inclisiran) were not feasible as ≤1 published study was available per medication for the pooled adherence measures. Given the current limited clinical data on inclisiran in many regions, we pooled the discontinuation rate reported in conference abstracts and articles for inclisiran. We also conducted meta-analyses to explore the following potential sources of heterogeneity to explain variation in the results of the included studies: demographic characteristics (ie, gender, age, and smoking status), disease history (ie, familial hypercholesterolemia, atherosclerotic cardiovascular disease, statin intolerance, diabetes, and hypertension), medication history (ie, history of lipid-lowering treatment, history of combination therapy with statin and ezetimibe, and history of statin or ezetimibe use alone), lipid or lipoprotein biomarkers (ie, total cholesterol [TC], LDL cholesterol, non-high-density lipoprotein cholesterol [non-HDL-C], high-density lipoprotein cholesterol [HDL-C], triglycerides [TG], apolipoprotein B [ApoB], and lipoprotein(a) [Lp(a)]), and publication year.

Sensitivity analyses were performed to assess the robustness of our findings. For medication adherence at specific time points, analyses were conducted in the following ways: 1) restricting to studies with a JBI checklist score of ≥6 to evaluate the influence of study quality on the pooled estimates; 2) applying log transformation to the implementation measures of MPR and PDC;^31^ and 3) using the Freeman-Tukey double arcsine transformation for proportions.^29^ For short- and long-term medication adherence, we conducted a sensitivity analysis to reduce heterogeneity by including only those studies that had data for both time periods in the three-level meta-analytic models. All statistical analyses were performed using the *metafor* and *meta* packages in R software, version 4.0.2 (R Foundation for Statistical Computing).^32^^,^^33^

## Results

### Study selection and characteristics

**Figure 1:**
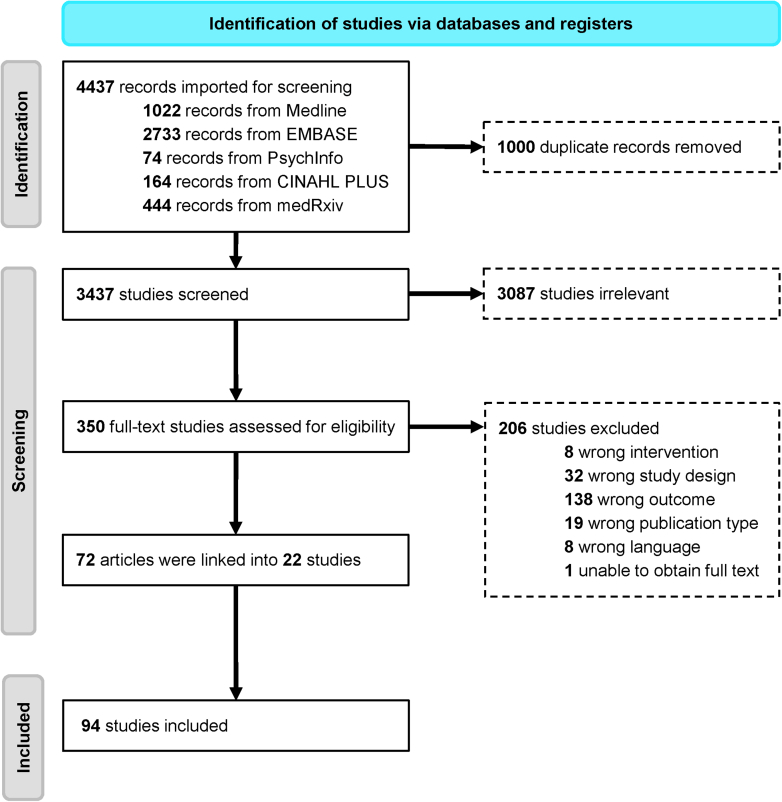
Preferred Reporting Items for Systematic Reviews and Meta-Analyses Flow Diagram of Included Studies

A total of 3,437 articles were retrieved from the literature search, of which 3,087 studies were excluded after screening titles and abstracts (Figure 1). A further 206 studies did not meet eligibility criteria upon full-text assessment (Supplemental Table 3). A final 94 studies reporting on 101 cohorts were included.^5^, ^6^, ^7^, ^8^, ^9^, ^10^, ^11^, ^12^^,^^34^, ^35^, ^36^, ^37^, ^38^, ^39^, ^40^, ^41^, ^42^, ^43^, ^44^, ^45^, ^46^, ^47^, ^48^, ^49^, ^50^, ^51^, ^52^, ^53^, ^54^^,^^55^, ^56^, ^57^, ^58^, ^59^, ^60^, ^61^, ^62^, ^63^, ^64^, ^65^^,^^15^^,^^66^, ^67^, ^68^, ^69^, ^70^, ^71^, ^72^, ^73^, ^74^, ^75^, ^76^, ^77^, ^78^, ^79^, ^80^, ^81^, ^82^, ^83^, ^84^, ^85^, ^86^, ^87^, ^88^, ^89^^,^^90^, ^91^, ^92^, ^93^, ^94^, ^95^, ^96^, ^97^, ^98^, ^99^, ^100^, ^101^, ^102^, ^103^, ^104^, ^105^, ^106^, ^107^, ^108^, ^109^, ^110^, ^111^, ^112^, ^113^ The distribution of studies by publication type, medication adherence phase, country, and data source are shown in Supplemental Table 4A. Most studies were published as full journal articles (n = 60), with the remainder as conference abstracts or brief reports (n = 34). Studies were primarily conducted in the United States (n = 31) and Europe (n = 47) with the remainder from other regions (n = 16). Data sources also varied widely, with 29 studies integrating multiple data sources.

Definitions of outcome measures for medication adherence phases (initiation, adherence, persistence, and reinitiation) varied across studies, with specific outcome definitions and characteristics of individual studies summarized in Supplemental Table 4B. The 56 studies eligible for meta-analysis enrolled a total of 75,092 individuals. Among them, 2 studies (3.6%) evaluated alirocumab only, 10 studies (17.9%) evaluated evolocumab only, 41 studies (73.2%) evaluated moAb, and 3 studies (5.4%) evaluated inclisiran. The mean (SD) age was 62.61 (11.9) years, 58.0% were males. A summary of baseline population characteristics by medication adherence measure is provided in Supplemental Figure 5. In addition, some studies reported PCSK9 inhibitor adherence in separate cohorts, for example, homozygous and heterozygous familial hypercholesterolemia, and the characteristics of these 56 eligible studies and cohorts are presented in Table 1.

**Table 1:** Characteristics of Studies Included in a Meta-Analysis

Lead Author, Year	Sample Size	Region	Data Source	PCSK9 Inhibitor Drugs Assessed	Medication Adherence Phase Studied	Outcome Definition
Alonso, 2023	696	Europe	Outpatient specialty clinics	• Alirocumab; Evolocumab	• Persistence • Reinitiation	Nonpersistent: gap ≥60 days
Arca, 2023a^a^	1,263	Europe	Database	• Alirocumab; Evolocumab	• Implementation • Persistence	Adherence reported as % Adherent: Adherence ≥80% Nonpersistent: discontinued the therapy before the expected observation
Arca, 2023b^a^	36					
Barrios, 2020	186	Europe	Database	• Evolocumab	• Implementation • Persistence	Adherent: Adherence ≥80% Nonpersistent: gap not specified
Bosch, 2024	193	Europe	Database	• Alirocumab; Evolocumab	• Persistence	Nonpersistent: gap not specified
Bradley, 2019	1,269	U.S.	Patient support program	• Alirocumab; Evolocumab	• Initiation	No. starting/No. prescribed
Cannon, 2021	554	U.S.	Mixed	• Alirocumab; Evolocumab	• Persistence	Nonpersistent: not receiving a PCSK9 inhibitor drug at 24 months postinitiation
Chai, 2023	63	Others (China)	Database	• Evolocumab	• Persistence	Nonpersistent: gap ≥30 days
Chlebus, 2022	55	Europe	Database	• Alirocumab; Evolocumab	• Persistence	Nonpersistent: gap not specified
Chng, 2022	80	Others (Singapore)	Outpatient specialty clinics	• Alirocumab; Evolocumab	• Persistence • Reinitiation	Nonpersistent: gap not specified
Davidson, 2020	55	U.S.	Outpatient specialty clinics	• Alirocumab; Evolocumab	• Initiation • Persistence	Initiation: No. starting/No. prescribed Nonpersistent: gap not specified
Davis, 2020	61	U.S.	Outpatient specialty clinics	• Alirocumab; Evolocumab	• Initiation • Persistence	Initiation: No. starting/No. eligible Nonpersistent: gap not specified
Donald, 2022	477	U.S.	Outpatient specialty clinics	• Alirocumab; Evolocumab	• Persistence	Nonpersistent: gap ≥60 days
Eloso, 2023a^a^	862	U.S.	Database	• Alirocumab; Evolocumab	• Implementation	Adherence assessed via MPR Adherent: MPR ≥80%
Eloso, 2023b^a^	526					
Eloso, 2023c^a^	269					
Eloso, 2023d^a^	594					
Engebretsen, 2022	1,266	Europe	Database	• Alirocumab; Evolocumab	• Implementation	Adherence assessed via PDC
Fairman, 2017	390	U.S.	Database	• Alirocumab; Evolocumab	• Persistence	Nonpersistent: gap ≥60 days
Fischer, 2021	237	Europe	Database	• Alirocumab; Evolocumab	• Persistence • Reinitiation	Nonpersistent: lost to follow-up (gap ≥365 days) or discontinued therapy
Galema-Boers, 2023	436	Europe	Outpatient specialty clinics	• Alirocumab; Evolocumab	• Persistence	Nonpersistent: gap not specified
Garcia-Pena, 2023	906	Others (Colombia)	Patient support program	• Evolocumab	• Persistence	Nonpersistent: gap ≥60 days
Gargiulo, 2024	771	Europe	Database	• Alirocumab; Evolocumab	• Persistence	Nonpersistent: permanently stopped therapy during the observation period
Gayoso-Rey, 2021	154	Europe	Mixed	• Alirocumab; Evolocumab	• Implementation • Persistence	Adherence assessed via MPR Nonpersistent: gap not specified
Goicoechea, 2022	60	Europe	Mixed	• Evolocumab	• Persistence	Nonpersistent: gap not specified
Gupta, 2023	578	Others (Canada, Colombia, Kuwait, Mexico,Saudi Arabia)	Mixed	• Evolocumab	• Persistence	Nonpersistent: gap ≥56 days
Gurgoze, 2018	164	Europe	Database	• Alirocumab; Evolocumab	• Persistence	Nonpersistent: gap not specified
Hines, 2018	13,151	U.S.	Database	• Alirocumab; Evolocumab	• Implementation • Persistence	Adherence assessed via PDC Adherent: PDC ≥80% Nonpersistent: gap ≥60 days
Iqbal, 2022	102	Others (United Arab Emirates)	Outpatient specialty clinics	• Evolocumab	• Persistence	Nonpersistent: gap ≥90 days
Iqbal, 2024	146	Others (United Arab Emirates)	Outpatient specialty clinics	• Inclisiran	• Persistence	Nonpersistent: gap ≥90 days
Kaufman, 2019	271	U.S.	Outpatient specialty clinics	• Alirocumab; Evolocumab	• Persistence	Nonpersistent: gap not specified
Khatib, 2022	48	Europe	Outpatient specialty clinics	• Alirocumab; Evolocumab	• Initiation • Persistence	Initiation: No. starting/No. eligible Nonpersistent: gap not specified
Kim, 2023	91	U.S.	Database	• Alirocumab; Evolocumab	• Persistence • Reinitiation	Nonpersistent: gap not specified
Koenig, 2024	1,940	Europe	Database	• Alirocumab; Evolocumab	• Persistence	Nonpersistent: gap ≥90 days
Kohli, 2017	80	Europe	Outpatient specialty clinics	• Alirocumab; Evolocumab	• Initiation • Persistence	Initiation: No. starting/No. eligible Nonpersistent: gap not specified
Lafratte, 2023	178	U.S.	Database	• Alirocumab; Evolocumab	• Implementation • Persistence	Adherence assessed via PDC Nonpersistent: gap ≥60 days
Lahoz, 2024	667	U.S.	Database	• Alirocumab; Evolocumab	• Implementation • Persistence	Adherence assessed via PDC Adherent: PDC ≥80% Nonpersistent: gap ≥60 days
Leitner, 2020	112	Europe	Outpatient specialty clinics	• Alirocumab	• Persistence	Nonpersistent: gap not specified
Maciejko, 2019	73	U.S.	Outpatient specialty clinics	• Alirocumab; Evolocumab	• Initiation • Persistence	Initiation: No. starting/No. eligible Nonpersistent: gap not specified
Mongiello, 2023	302	Europe	Database	• Alirocumab; Evolocumab	• Implementation	Adherence assessed via MPR Adherent: MPR ≥80%
Mulder, 2023	65	Europe	Outpatient specialty clinics	• Inclisiran	• Persistence	Nonpersistent: gap not specified
Muntner, 2024	16,588	U.S.	Database	• Alirocumab; Evolocumab	• Implementation • Persistence	Adherence assessed via PDC Adherent: PDC ≥80% Nonpersistent: gap ≥60 days
Nanchen, 2022	100	Europe	Outpatient specialty clinics	• Evolocumab	• Implementation • Persistence	Adherence reported as % Nonpersistent: gap not specified
Naoum, 2024	503	Other (Israel)	Database	• Inclisiran	• Persistence	Nonpersistent: gap ≥60 days
Oren, 2019	96	U.S.	Outpatient specialty clinics	• Alirocumab; Evolocumab	• Initiation	No. starting/No. approved
Parhofer, 2019	612	Europe	Outpatient specialty clinics	• Alirocumab	• Persistence	Nonpersistent: gap not specified
Piccinni, 2019	266	Europe	Database	• Alirocumab; Evolocumab	• Persistence • Reinitiation	Nonpersistent: gap ≥30 days
Rallidis, 2020	141	Europe	Outpatient specialty clinics	• Alirocumab; Evolocumab	• Persistence	Nonpersistent: gap not specified
Ray, 2023	1,951	Europe	Mixed	• Evolocumab	• Persistence	Nonpersistent: permanently stopped therapy during the observation period
Reynolds, 2019	287	U.S.	Outpatient specialty clinics	• Alirocumab; Evolocumab	• Initiation	No. starting/No. approved
Rymer, 2020	6,151	U.S.	Database	• Alirocumab; Evolocumab	• Persistence • Reinitiation	Nonpersistent: gap ≥30 days
Sheng, 2024	4,022	Others (Japan)	Database	• Evolocumab	• Persistence	Nonpersistent: gap ≥60 days
Stoekenbroek, 2017	238	Europe	Database	• Alirocumab; Evolocumab	• Persistence	Nonpersistent: gap not specified
Stummer, 2023	7,302	Europe	Database	• Alirocumab; Evolocumab	• Reinitiation	NA
Svensson, 2024	2,341	Europe	Database	• Evolocumab	• Implementation • Persistence	Adherence assessed via PDC Adherent: PDC ≥80% Nonpersistent: gap ≥56 days
Vicente-Valor, 2021	115	Europe	Database	• Alirocumab; Evolocumab	• Persistence	Nonpersistent: discontinued therapy prior to the cutoff date for analysis
Warden, 2021	89	U.S.	Outpatient specialty clinics	• Alirocumab; Evolocumab	• Initiation • Persistence	Initiation: No. starting/No. prescribed Nonpersistent: gap not specified
Wong, 2023	3,162	U.S.	Database	• Alirocumab; Evolocumab	• Implementation	Adherence assessed via PDC
Zafrir, 2018	101	Others (Israel)	Outpatient specialty clinics	• Alirocumab; Evolocumab	• Initiation • Persistence	Initiation: No. starting/No. approved Nonpersistent: gap not specified
Zafrir, 2020	1,600	Others (Israel)	Database	• Alirocumab; Evolocumab	• Implementation • Persistence • Reinitiation	Adherence assessed via PDC Adherent: PDC ≥80% Nonpersistent: gap ≥60 days

MPR = medication possession ratio; NA = not applicable; No. = number; PDC = proportion of days covered.

a Cohorts reported within a study are indicated by a lowercase letter following the publication year.

### Narrative synthesis of medication adherence outcomes and measures

Medication adherence was most frequently assessed for the persistence phase (n = 74), followed by the implementation phase (n = 44). In contrast, other adherence phases, such as initiation (n = 22) and reinitiation (n = 11), were less frequently evaluated. There was variability in the reported adherence measures, and the inconsistent definitions or limited number of studies restricted our ability to pool results for some measure of implementation and persistence.

Nearly all studies (n = 85) evaluated medication adherence to moAb PCSK9 inhibitors, while 7 studies evaluated siRNA PCSK9 inhibitor, of which 2 were included in meta-analysis.^15^^,^^70^ A summary of the pooled results from the siRNA studies for our primary outcomes is presented in Supplemental Material Section 6. Only 2 studies, published as conference abstracts, assessed all 3 PCSK9 inhibitors and reported on the implementation and persistence phases of medication adherence. Although their follow-up ≤1 year, the results suggested that inclisiran could be an alternative to enhance adherence.^86^^,^^91^

### Study quality assessment

Overall 34 (61.8%) studies met the low risk of bias threshold (≥6 points), while 21 (38.2%) were classified as high-risk of bias (Supplemental Figure 7). The median quality score across the studies was 6 (Q1-Q3: 5-7), indicating generally reasonable study quality (Supplemental Table 7).

### Meta-analysis of medication adherence outcomes

#### Initiation phase

**Figure 2:**
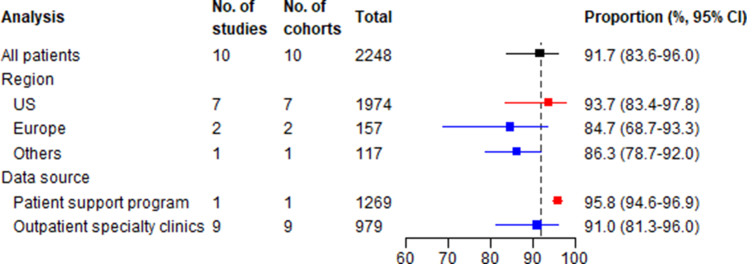
Summary Forest Plot for Proportion of Patients Initiating PCSK9 Inhibitors The analysis column refers to whether the estimate is for the overall pooled effect (“All patients”) or a stratified analysis by geographical region or data source. The total column show the total number of patients included in each meta-analysis.

The pooled proportion of patients initiating a PCSK9 inhibitor was 91.7% (95% CI: 83.6-96.0; I^2^ = 94.2%) (Figure 2, Supplemental Figure 8A). Subgroup analysis by region showed the highest initiation rates in the United States, followed by Europe and others. When analyzed by data source, there was a slightly higher initiation rate (95.8%, 95% CI: 94.6-96.9) in patient support programs than in outpatient specialty clinics (91.0%, 95% CI: 81.3-96.0) (Supplemental Figure 9A). The results of meta-regression suggest that familial hypercholesterolemia was associated with lower PCSK9 inhibitor initiation, while patients using statins, ezetimibe, or a combination of both at baseline were more likely to initiate a PCSK9 inhibitor (Supplemental Table 10).

#### Implementation phase

Studies reporting on adherence implementation used various implementation measures (Supplemental Table 4B). We present pooled prevalence estimates for the 3 most reported adherence measures at the time points with the largest number of cohorts: MPR, PDC, and the proportion of patients with ≥80% adherence implementation. Summary results for other less frequently reported time points with smaller sample sizes are reported in Supplemental Table 11A.

##### Medication possession ratio at 6 and 24 months

**Figure 3:**
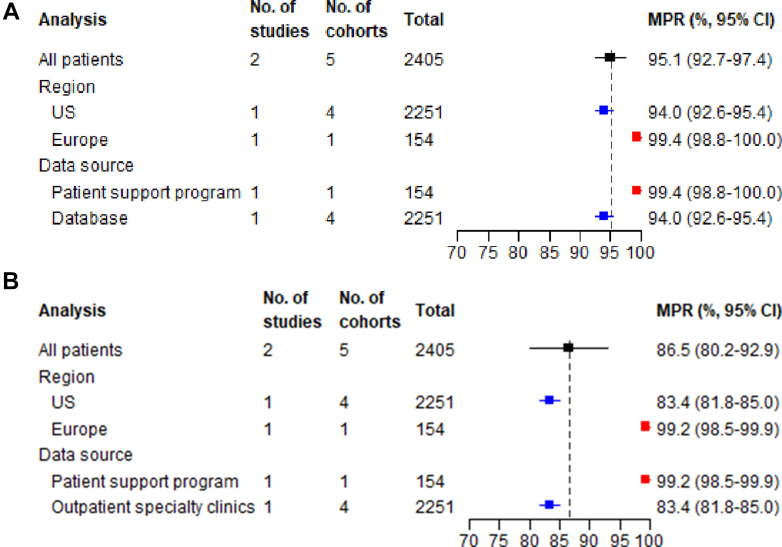

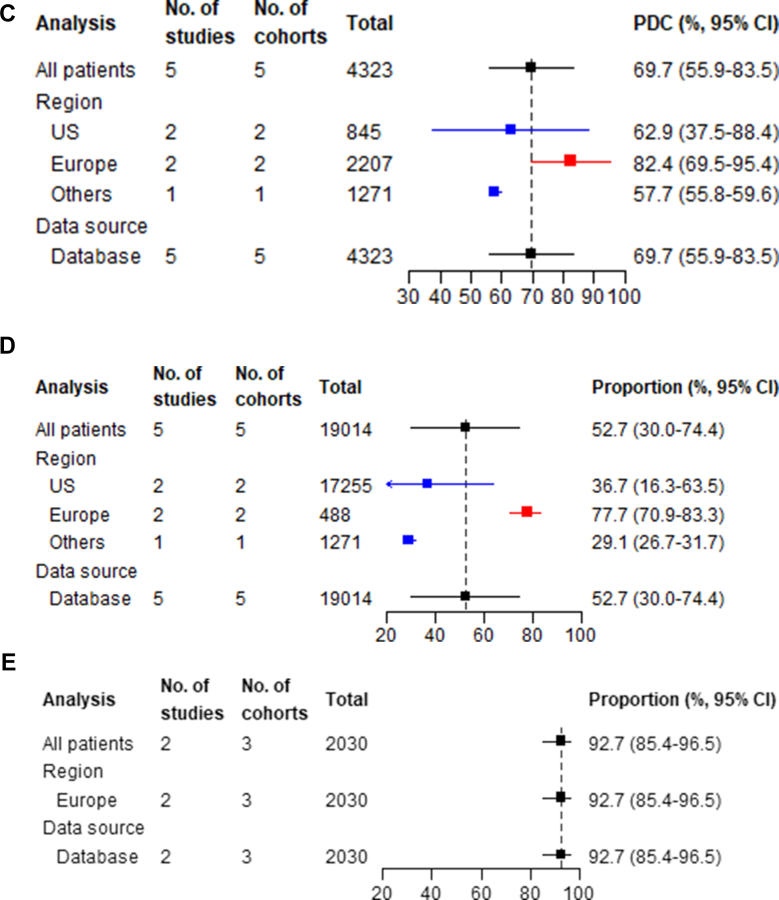
**Summary Forest Plots for Implementation Phase Measures** The analysis column refers to whether the estimate is for the overall pooled effect (”All patients”) or a stratified analysis by geographical region or data source. The total column show the total number of patients included in each meta-analysis. (A) MPR at 6 months; (B) MPR at 24 months; (C) proportion of days covered (PDC) at 12 months; (D) proportion of adherence ≥80% (total population as denominator) at 12 months; (E) proportion of adherence ≥80% (persistent population as denominator) at 12 months. MPR = medication possession ratio.

The pooled MPR was 95.1% (95% CI: 92.7-97.5; I^2^ = 98.4%) (Figure 3A) at 6 months and 86.5% (95% CI: 80.2-92.9; I^2^ = 99.7%) (Figure 3B, Supplemental Figure 8B) at 24 months. Subgroup analysis by region showed consistently higher MPR in Europe compared to the United States at both timepoints, and stratification by data source showed higher MPR in patient support programs than databases (Supplemental Figure 9B). Meta-regression consistently indicated diabetes mellitus was associated with poorer MPR at both time points (Supplemental Table 10).

##### Proportion of days covered at 12 months

The PDC at 12 months was 69.7% (95% CI: 55.9-83.5; I^2^ = 99.8%) (Figure 3C, Supplemental Figure 8C). Studies from Europe had the highest PDC, followed by the United States and other regions (Supplemental Figure 9C). All studies used databases so subgroup analysis by data source could not be performed. Meta-regression suggested that female patients, those with diabetes mellitus, and older age were associated with lower PDC at 12 months (Supplemental Table 10).

##### Adherence ≥80% at 12 months

Studies assessing PCSK9 inhibitor adherence using a ≥80% implementation threshold for either MPR or PDC demonstrated significant differences based on the choice of population denominator. Using the total population as the denominator yielded a pooled proportion of 52.7% (95% CI: 30.0-74.4; I^2^ = 99.4%) (Figure 3D, Supplemental Figure 8D), while using the persistent population showed markedly higher proportion at 92.7% (95% CI: 85.4-96.5; I^2^ = 99.1%) (Figure 3E).

Geographic variations were prominent in analyzable population studies, with Europe demonstrating the highest adherence (77.7%; 95% CI: 70.9-83.3), followed by the United States (36.7%; 95% CI: 16.3-63.5) and other regions (29.1%; 95% CI: 26.7-31.7) (Supplemental Figure 9D). All studies used databases so no subgroup analysis was conducted. Meta-regression for both methodologies consistently indicated diabetes mellitus and older age were associated with poorer adherence ≥80% at 12 months (Supplemental Table 10).

#### Persistence phase

##### Proportion of persistent patients at 12 months

**Figure 4:**
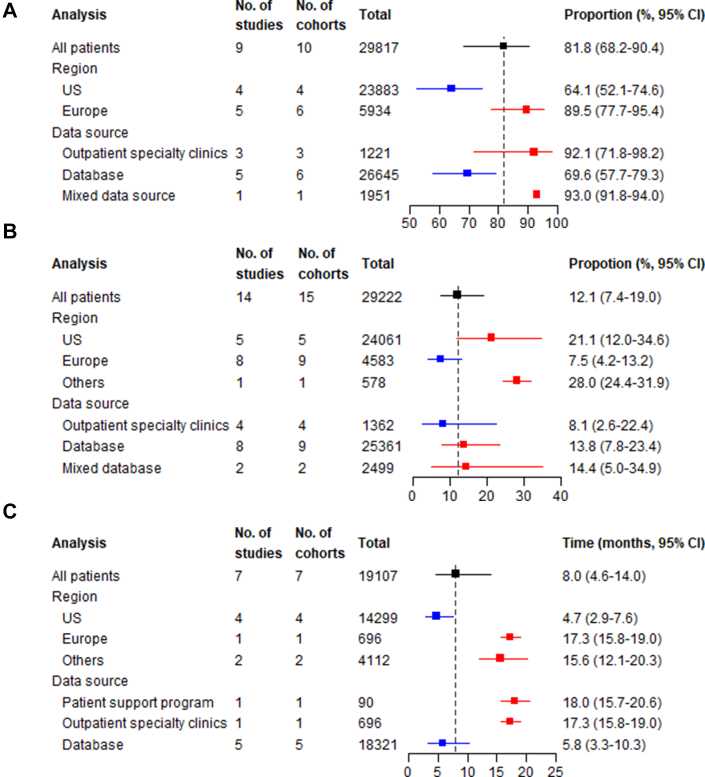
**Summary Forest Plots for Persistence Phase Measures** The analysis column refers to whether the estimate is for the overall pooled effect (”All patients”) or a stratified analysis by geographical region or data source. The total column show the total number of patients included in each meta-analysis. (A) Proportion of persistent patients at 12 months; (B) discontinuation rate at 12 months; (C) mean time from PCSK9 inhibitor initiation to permanent discontinuation (months).

The proportion of persistent patients at 12 months was 81.8% (95% CI: 68.2-90.4; I^2^ = 99.1%) (Figure 4A, Supplemental Figure 8E). European countries demonstrated higher persistence (89.5%; 95% CI: 77.7-95.4) than the United States (64.1%; 95% CI: 52.1-74.6) and outpatient specialty clinics (92.1%; 95% CI: 71.8-98.2), and mixed data sources (93.0%; 95% CI: 91.8-94.0) showed higher persistence than databases (69.6%; 95% CI: 57.7-79.3) (Supplemental Figure 9E). Meta-regression suggested statin intolerance, statin-ezetimibe combination use, and higher HDL-C were associated with a higher persistence rate, while diabetes mellitus, hypertension, higher TC, and non-HDL-C predicted poorer persistence at 12 months (Supplemental Table 10).

##### Discontinuation rate at 12 months and reasons for PCSK9 inhibitor discontinuation

The 12-month discontinuation rate was 12.1% (95% CI: 7.4-19.0; I^2^ = 98.9%) (Figure 4B, Supplemental Figure 8F). The highest discontinuation rates were observed in other regions (28.0%, 95% CI: 24.4-31.9), followed by the United States (21.1%, 95% CI: 12.0-34.6) and Europe (8.1%, 95% CI: 4.2-13.2). When stratified by data source, discontinuation rates were similar across outpatient specialty clinics (8.1%, 95% CI: 2.6-22.4), databases (13.8%, 95% CI: 7.8-23.4), and mixed data sources (14.4%, 95% CI: 5.0-34.9) (Supplemental Figures 9F). Meta-regression analysis suggested that hypertension, higher TC, and higher non-HDL-C were associated with higher discontinuation rate at 12 months (Supplemental Table 10). Among studies reporting reasons for PCSK9 inhibitor discontinuation, adverse effects (45.1%, 95% CI: 30.4-60.6; I^2^ = 87.2%), and patient decision (28.8%, 95% CI: 15.8-46.5; I^2^ = 86.9%) were the most common categories for discontinuation, but the proportion of patients discontinuing for each reason varied widely across studies (Supplemental Figure 7F). Medication cost of PCSK9 inhibitor therapy accounted for 19.4% (95% CI: 8.4%-38.8%; I^2^ = 85.8%) of discontinuations.

##### Time from PCSK9 inhibitor initiation to permanent discontinuation

The mean duration from PCSK9 inhibitor initiation to permanent treatment discontinuation was 8.00 months (95% CI: 4.57-13.98; I^2^ = 99.8%) (Figure 4C, Supplemental Figure 8G). Studies from Europe demonstrated the longest mean persistence at 17.33 months (95% CI: 15.80-19.01), followed by other regions at 15.63 months (95% CI: 12.06-20.26), the United States showing the shortest duration at 4.70 months (95% CI: 2.90-7.62). When stratified by data source, patient support programs recorded the longest persistence (18.00 months, 95% CI: 15.70-20.63), followed by outpatient specialty clinics (17.33 months, 95% CI: 15.80-19.01), while database studies showed markedly shorter persistence (5.83 months, 95% CI: 3.31-10.26) (Supplemental Figure 9G). Meta-regression suggested that baseline lipid-lowering drug use was associated with a longer time from PCSK9 inhibitor initiation to permanent interruption, while statin use predicted shorter duration (Supplemental Table 10).

#### Reinitiation phase

##### Resumption rate

**Figure 5:**
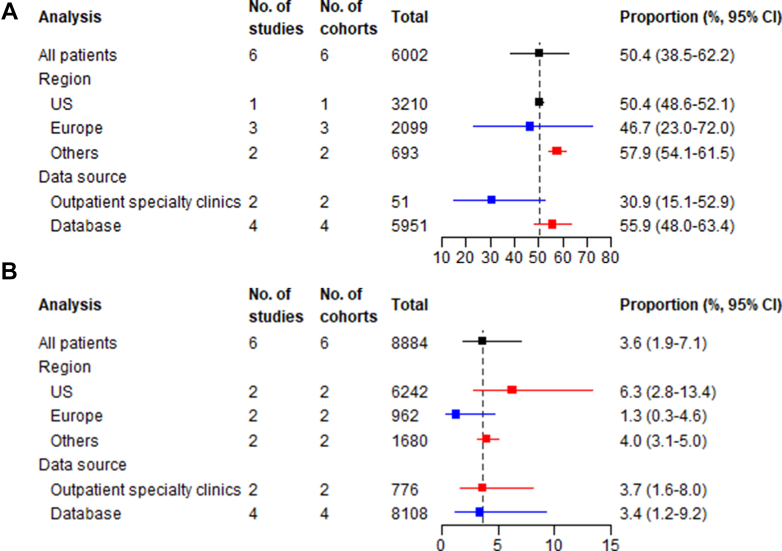
**Summary Forest Plots for Reinitiation Phase Measures** The analysis column refers to whether the estimate is for the overall pooled effect (”All patients”) or a stratified analysis by geographical region or data source. The total column show the total number of patients included in each meta-analysis. (A) The proportion of patients reinitiating a PCSK9 inhibitor after a temporary interruption and (B) the proportion of patients switching a PCSK9 inhibitor.

The rate of PCSK9 inhibitor resumption after a temporary interruption was 50.4% (95% CI: 38.5-62.2; I^2^ = 87.0%) (Figure 5A, Supplemental Figure 8H). Other regions showed highest resumption rate (57.9%, 95% CI: 54.1-61.5), vs the United States (50.4%, 95% CI: 48.6% to 52.1%) and Europe (46.7%, 95% CI: 23.0%-72.0%). Database studies reported higher resumption (55.9%, 95% CI: 48.0-63.4) compared to outpatient specialty clinic data (30.9%, 95% CI: 15.1-52.9) (Supplemental Figure 9H).

##### Switching rate

The proportion of patients who switched between moAb PCSK9 inhibitor drugs was 3.6% (95% CI: 1.9-7.1; I^2^ = 81.2%) (Figure 5B, Supplemental Figure 8I). The United States showed highest switching (6.3%, 95% CI: 2.8-13.4), followed by other regions (4.0%, 95% CI: 3.1-5.0), and substantially lower rates in Europe (1.3%, 95% CI: 0.3-4.6). Switching was similar by data source (Supplemental Figures 9I).

### Multilevel meta-analyses with time period as a moderator

Three-level random effects meta-analyses showed that MPR and the proportion of persistent patients were lower in the long-term compared to short-term (Table 2). Adherence as measured by MPR significantly decreased from 91.9% (95% CI: 87.1-96.8) to 85.4% (95% CI: 80.6-90.2), and the proportion of persistent patients significantly declined from 80.1% (95% CI: 70.5-87.1) to 67.3% (95% CI: 55.1-77.6). Adherence ≥80% remained stable over time, showing comparable estimates in both the short- and long-term. In contrast, discontinuation rates significantly increased in long-term (22.3%, 95% CI: 14.8-32.3) as compared to the short-term (13.5%, 95% CI: 9.9-18.1). Heterogeneity was substantial across outcomes, with discontinuation rates showing the greatest between-study variability (Supplemental Table 11B). These findings suggest that implementation and persistence decrease over time, with progressively more patients discontinuing therapy in the long-term.

**Table 2:** Moderation Analyses in the Three-Level Random-Effects Models for Selected Measures of Medication Adherence Implementation and Persistence

Time Point	MPR (%)				Adherence ≥80% (%)				Proportion of Persistent Patients (%)^a^				Discontinuation Rate (%)			
	N	Estimate (95% CI)	*Q*_*M*_	*P* Value	N	Estimate (95% CI)	*Q*_*M*_	*P* Value	N	Estimate (95% CI)	*Q*_*M*_	P Value	N	Estimate (95% CI)	*Q*_*M*_	*P* Value
Studies with time points in either short-term or long-term																
Short-term	8	91.94 (87.13-96.76)	7.12	0.008	7	91.42 (94.74-86.30)	0.01	0.920	20	80.09 (70.51-87.12)	6.36	0.012	34	13.49 (9.94-18.05)	12.04	<0.001
Long-term	10	85.39 (80.55-90.22)			6	91.63 (82.39-96.24)			9	67.32 (55.12-77.55)			13	22.32 (14.75-32.29)		
Studies with time points in both short-term and long-term																
Short-term	8	91.94 (87.13-96.76)	7.12	0.008	7	91.42 (94.74-86.30)	0.01	0.920	8	86.22 (77.50-91.91)	12.17	<0.001	8	7.70 (5.33-10.98)	12.67	<0.001
Long-term	10	85.39 (80.55-90.22)			6	91.63 (82.39-96.24)			8	68.38 (56.88-78.01)			10	14.52 (5.97-31.26)		

MPR = medication possession ratio.

N indicates the number of measurements within the specified timeframe, since one study could have measurements of medication adherence at multiple time points. Short-term refers to measurements ≤12 months and long-term refers to measurements >12 months.

a The denominator of proportion of adherence ≥80% (%) is estimated using the persistent population. There was insufficient data to estimate the measure of proportion of adherence ≥80% (%) with the denominator of total population.

### Sensitivity analyses and assessment of publications bias

Supplemental Table 12 summarizes the results of the sensitivity analyses, which demonstrated comparable pooled results across all medication adherence measures at specific time points, supporting the robustness of our main findings. There was limited evidence of funnel plot asymmetry (Supplemental Figure 13), and Egger's tests were not statistically significant (Supplemental Table 13).

## Discussion

This is the first systematic review to determine the prevalence of medication adherence to PCSK9 inhibitors across all adherence phases—initiation, implementation, persistence, and reinitiation (Central Illustration). By pinpointing key adherence challenges, this study provides insights to guide targeted interventions, inform evidence-based guidelines, and develop novel strategies that improve adherence to PCSK9 inhibitors.

**Central Illustration:**
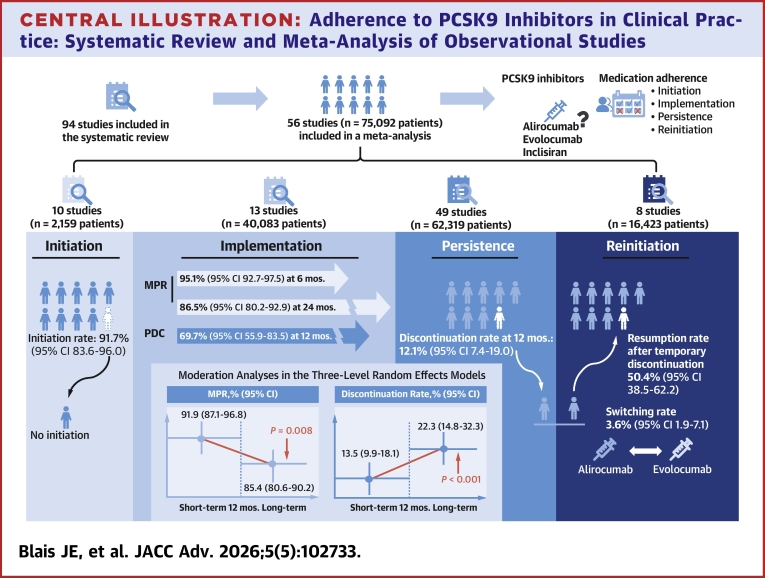
**Adherence to PCSK9 Inhibitors in Clinical Practice: Systematic Review and Meta-Analysis of Observational Studies** Mos. = months; MPR = medication possession ratio; PDC = proportion of days covered.

Evidence from this review contrasts with certain adherence outcomes reported in PCSK9 inhibitor clinical trials. A pooled analysis of 6 randomized, double-blind, controlled ODYSSEY trials, demonstrated high levels of implementation to alirocumab, with a mean (SD) overall treatment adherence of 98.0% (4.4) over 1 to 2 years.^114^ In contrast, our meta-analysis found much lower implementation, with a pooled 12-month PDC of 69.7% and 24-month MPR of 86.5%. Regarding the persistence phase, the FOURIER open-label extension study reported a 14.0% overall premature discontinuation rate over 5 years among 6,635 patients randomized to evolocumab.^115^^,^^116^ Similarly, the ODYSSEY OUTCOMES trial, reported a 14.2% premature discontinuation rate for alirocumab.^117^ Our results partly align with findings from these landmark trials, showing a similar 12% discontinuation rate at 12 months. The few real-world studies of inclisiran included in this review, also suggest poorer medication adherence than what has been observed in inclisiran RCTs. ORION-8, a multicenter open-label extension of phase 2 (ORION-3) and phase 3 (ORION-9, -10, −11) trials involving 3,274 patients with a mean cumulative exposure of 3.7 years, demonstrated that only 2.4% of patients discontinued inclisiran due to treatment emergent adverse effects.^118^ In contrast, our study found a nearly three-fold higher discontinuation rate of 7.2% across all studies and 6.3% at 3 months. Nevertheless, ongoing studies such as VICTORION-REAL (NCT05399992) will assess long-term adherence to inclisiran.

Our findings suggest substantial disparities in PCSK9 inhibitor adherence globally. European populations exhibited better implementation and persistence than those in the United States, likely attributable to health care system accessibility, cost-sharing structures, study design, and medication monitoring practices.^119^, ^120^, ^121^, ^122^, ^123^ U.S. health insurers implement restrictive measures such as prior authorization, step therapy, and complex appeals processes, if initial coverage for PCSK9 inhibitors is denied.^120^ These managed care formulary restrictions imposed by insurers have been shown to negatively impact medication adherence, aligning with our subgroup analyses by region for most outcomes.^124^ Adherence also varied by data source, with superior adherence observed in patient support programs and specialty clinics compared to database studies. This reflects differences in patient selection and structures of care in specialty clinics settings, where patients are often highly motivated and additional resources are available to proactively resolve barriers to medication adherence. Our findings also emphasize the need to further study adherence to PCSK9 inhibitors in diverse settings, including low- and middle-income regions and in primary care.

Because of the differences in operationalizing adherence measures across studies, the binary nature of many of our outcomes, and the wide range of study sample sizes (and hence precision of CIs), it is not surprising that our meta-analyses had high *I*^*2*^ values. Prior work has shown that studies of prevalence have a median *I*^*2*^ of 96.9%, although this does not always correspond to high heterogeneity and *I*^*2*^ should therefore be interpreted with care.^125^ Rather, assessment of between-study heterogeneity can be informed by visual inspection of the range of study-level estimates in the forest plots.^125^ For example, estimates for MPR at 6 months ranged from 92.1% to 99.4% (Supplemental Figure 8i) implying low heterogeneity, despite an *I*^*2*^ of 98%, whereas the discontinuation rate at 12 months ranged from 1.1% to 48.7% (Supplemental Figure 8Fi) implying higher heterogeneity, with a similarly high *I*^*2*^ of 99%.

Our meta-regression analysis identified several potentially important study-level predictors of PCSK9 inhibitor adherence. Patients with diabetes and older individuals demonstrated poorer adherence implementation, potentially due to competing treatment demands or therapeutic inertia.^126^, ^127^, ^128^ Furthermore, hypertension and elevated lipid levels (TC and non-HDL-C) were significant predictors of reduced treatment persistence. This likely reflects both the asymptomatic nature of these conditions—leading to patient perceptions of wellness without medication—and the pill burden associated with managing multiple cardiovascular medications.^129^

Our findings highlight distinct adherence challenges across treatment phases. Optimal management should involve early implementation support for at-risk patients, sustained adherence monitoring beyond the first 6 months of therapy, and reinforced persistence strategies during routine clinic visits. Addressing cost barriers and enhancing care coordination through health care system interventions are also critical to long-term treatment success.^120^^,^^123^

These clinical challenges are compounded by methodological issues in adherence research—particularly the wide variability in how adherence implementation, persistence, and discontinuation are defined and measured.^4^ Such inconsistencies reflect both the complexity of medication-taking behavior and heterogeneous reporting standards. Adherence was often arbitrarily defined, several studies using MPR or PDC threshold of 80% or greater, despite its subjective nature and potential to oversimplify complex behaviors. In some studies, adherence measure was calculated using only the persistent population as the denominator, potentially inflating adherence estimates by excluding patients who discontinued treatment. Similarly, discontinuation was typically defined as a treatment gap exceeding a specific period, most commonly 90 days. To minimize bias caused by arbitrary definitions of adherence, we aggregated adherence estimates only when the original studies used comparable definitions, ensuring that pooled estimates reflected consistent operational criteria.

These encountered inconsistencies represent a common challenge in adherence-related research.^3^ Even commonly used adherence metrics such as MPR and PDC can yield different results for the same population. This lack of standardized definitions highlights the need for consistent criteria to improve comparability and accuracy in real-world medication, and specifically, PCSK9 inhibitor medication adherence research. Future studies using adherence as a key variable should consider the implications for evidence synthesis, to ensure their reporting facilitates integration into meta-analyses.

Currently, there is no universally accepted gold standard for measuring medication adherence, as all existing methods have inherent strengths and limitations.^130^, ^131^, ^132^ Self-reported adherence is simple and cost-effective but prone to recall and social desirability bias.^130^^,^^131^^,^^133^^,^^134^ Persistence measures derived from databases, such as claims data, cannot confirm actual medication use and may misclassify patients who switch insurers as nonadherent.^130^^,^^131^^,^^134^ Additionally, refill records may attribute treatment gaps to patients, even when discontinuation was clinician-directed.^130^^,^^131^^,^^134^ Despite these limitations, database-derived methods provide valuable insights into refill timeliness and medication use continuity.

Our review has determined the prevalence of PCSK9 inhibitor adherence and reasons for discontinuation in typical clinical practice for both moAbs and inclisiran. Notably, our review is among the first to examine reinitiation rates following temporary PCSK9 inhibitor discontinuation, offering novel insights into real-world medication-taking behavior beyond initial persistence especially for chronic medications administered by injection. These prevalence estimates can inform clinicians, serve as model inputs for decision makers and pharmacoeconomic researchers, and provide evidence to pharmaceutical companies to quantify the current unmet needs for novel treatments, such as oral PCSK9 inhibitors. Our analysis included several studies with data on over 70,000 patients, providing real-world insights that enhance the generalizability of the results. The inclusion of emerging evidence on inclisiran is valuable since it has been suggested as an alternative to moAbs, to enhance medication adherence.^14^ Lastly, we used a methodologically rigorous approach informed by standardized adherence metrics,^3^^,^^4^ conducted quality assessments via JBI critical appraisal checklist,^19^ and restricted the meta-analyses to fully reported studies, with sensitivity analyses to ensure robustness.

### Study Limitations

Despite strict eligibility criteria, some clinical heterogeneity among patients and variability in adherence measures exist across the included studies, which could complicate cross-study comparisons and introduce bias. To minimize heterogeneity, we aggregated adherence estimates only when the original studies used comparable definitions, and call for standardized criteria and reporting practices in future research. The JBI prevalence tool could underestimate the study-level risk of bias because it was not designed for longitudinal outcomes: medication persistence or discontinuation, although in the studies included in our review, persistence was often measured as a point prevalence (proportion) at a specific follow-up time. While statistical heterogeneity across studies was expected and explored through meta-regression and subgroup analyses, these approaches did not fully account for all heterogeneity. Despite an extensive literature search, only a limited number of studies on inclisiran were identified, even fewer with direct comparisons to moAbs. Consequently, we were unable to determine whether inclisiran offers better adherence than moAbs, highlighting a critical gap in the evidence. Long-term comparative studies are needed to clarify the role of PCSK9 inhibitor dosing frequency in improving medication adherence, particularly inclisiran’s biannual dosing regimen. Most of the included studies were conducted in high-income regions, while evidence from low- and middle-income regions remains scarce. Given the substantial differences in health care systems and access to PCSK9 inhibitors across regions, our findings may have limited applicability in low- and middle-income settings, and further research in low-middle income regions should be prioritized. Medication adherence may be overestimated because many included studies were conducted in specialty settings, where patients receive more intensive support, limiting generalizability to the broader population. Lastly, reporting biases such as selective outcome reporting and unpublished data are possible, especially among observational studies, where study registration is not required for publication.

## Conclusions

In this systematic review, initiation of PCSK9 inhibitor therapy was generally high, while implementation and persistence declined over time. Given the limited number of studies with follow-up beyond 24 months and on inclisiran, additional research is needed to understand long-term medication adherence to all PCSK9 inhibitors.

## Funding support and author disclosures

This study was supported by The University of Hong Kong, Hong Kong, SAR China (Enhanced New Staff Start-up Research Grant & URC Seed Funding for Basic Research for New Staff). Dr Blais reports research grants from the Research Fund Secretariat of the Food and Health Bureau (Health and Medical Research Fund [HMRF], Hong Kong SAR) and consulting fees from the Institute of Medical Advancement and Clinical Excellence (IMACE) Hong Kong, outside of this work. Dr Chan reports research grants from Health Research Council of New Zealand, Auckland Medical Research Foundation, Asthma UK, University of Auckland, Oakley Mental Health Foundation, Chorus Ltd, AstraZeneca, World Health Organization, and the University of Hong Kong, outside of this work and all paid to her institution (the University of Auckland); is current clinical director of Asthma NZ; reports consultancy fees from AcademyeX and Spoonful of Sugar Ltd; reports travel support from AstraZeneca; reports advisory board fees from CSL Seqirus, AstraZeneca, and GSK; and is a member of Respiratory Effectiveness Group (REG), ESPACOMP research, policy and implementation committee, Global Asthma Network steering committee, and co-chair for the European Respiratory Society Clinical Research Collaboration “CONNECT.” All other authors have reported that they have no relationships relevant to the contents of this paper to disclose.
