# Supplementary material for: Adherence to PCSK9 Inhibitors in Clinical Practice: Systematic Review and Meta-Analysis of Observational Studies
Source: JACC Adv. 2026 Apr 17;5(5):102733. doi: 10.1016/j.jacadv.2026.102733 (PMC13098587; doi:10.1016/j.jacadv.2026.102733)
Supplement: Supplemental_Material [file mmc1.docx]

**Supplementary Material**

**Adherence to PCSK9 inhibitors in clinical practice: systematic review and meta-analysis of observational studies**

Contents
[1. PRISMA Checklist 3](#_Toc222227864)

[2. Search Strategy 6](#_Toc222227865)

[3. List of excluded studies and exclusion reasons 8](#_Toc222227866)

[4. List of included studies 27](#_Toc222227867)

[5. Boxplots of Baseline Population Characteristics 43](#_Toc222227868)

[6. Summary of results from siRNA PCKS9 inhibitor studies 53](#_Toc222227869)

[7. Quality Assessment 54](#_Toc222227870)

[8. Forest plots (random effects) of adherence outcomes 57](#_Toc222227871)

[9. Subgroup analysis 64](#_Toc222227872)

[10. Meta-regression analysis 72](#_Toc222227873)

[11. Additional results on adherence outcomes 74](#_Toc222227874)

[12. Sensitivity analysis 75](#_Toc222227875)

[13. Assessment of publication bias 82](#_Toc222227876)

[14. Reference list of all included studies 87](#_Toc222227877)

# PRISMA Checklist

**Table S1.** PRISMA 2020 checklist

| **Section and Topic** | **Item #** | **Checklist item** | **Reported on page #** |
| --- | --- | --- | --- |
| **TITLE** | | |  |
| Title | 1 | Identify the report as a systematic review. | 1 |
| **ABSTRACT** | | |  |
| Abstract | 2 | See the PRISMA 2020 for Abstracts checklist. | 4-5 |
| **INTRODUCTION** | | |  |
| Rationale | 3 | Describe the rationale for the review in the context of existing knowledge. | 6 |
| Objectives | 4 | Provide an explicit statement of the objective(s) or question(s) the review addresses. | 6-7 |
| **METHODS** | | |  |
| Eligibility criteria | 5 | Specify the inclusion and exclusion criteria for the review and how studies were grouped for the syntheses. | 8-9 |
| Information sources | 6 | Specify all databases, registers, websites, organisations, reference lists and other sources searched or consulted to identify studies. Specify the date when each source was last searched or consulted. | 8 |
| Search strategy | 7 | Present the full search strategies for all databases, registers and websites, including any filters and limits used. | Supplementary Material Table S2 |
| Selection process | 8 | Specify the methods used to decide whether a study met the inclusion criteria of the review, including how many reviewers screened each record and each report retrieved, whether they worked independently, and if applicable, details of automation tools used in the process. | 10 |
| Data collection process | 9 | Specify the methods used to collect data from reports, including how many reviewers collected data from each report, whether they worked independently, any processes for obtaining or confirming data from study investigators, and if applicable, details of automation tools used in the process. | 11 |
| Data items | 10a | List and define all outcomes for which data were sought. Specify whether all results that were compatible with each outcome domain in each study were sought (e.g. for all measures, time points, analyses), and if not, the methods used to decide which results to collect. | 9-10 |
|  | 10b | List and define all other variables for which data were sought (e.g. participant and intervention characteristics, funding sources). Describe any assumptions made about any missing or unclear information. | 11 |
| Study risk of bias assessment | 11 | Specify the methods used to assess risk of bias in the included studies, including details of the tool(s) used, how many reviewers assessed each study and whether they worked independently, and if applicable, details of automation tools used in the process. | 11 |
| Effect measures | 12 | Specify for each outcome the effect measure(s) (e.g. risk ratio, mean difference) used in the synthesis or presentation of results. | 11-13 |
| Synthesis methods | 13a | Describe the processes used to decide which studies were eligible for each synthesis (e.g. tabulating the study intervention characteristics and comparing against the planned groups for each synthesis (item #5)). | 11-13 |
|  | 13b | Describe any methods required to prepare the data for presentation or synthesis, such as handling of missing summary statistics, or data conversions. | 11-13 |
|  | 13c | Describe any methods used to tabulate or visually display results of individual studies and syntheses. | 11-13 |
|  | 13d | Describe any methods used to synthesize results and provide a rationale for the choice(s). If meta-analysis was performed, describe the model(s), method(s) to identify the presence and extent of statistical heterogeneity, and software package(s) used. | 11-13 |
|  | 13e | Describe any methods used to explore possible causes of heterogeneity among study results (e.g. subgroup analysis, meta-regression). | 13-14 |
|  | 13f | Describe any sensitivity analyses conducted to assess robustness of the synthesized results. | 13-14 |
| Reporting bias assessment | 14 | Describe any methods used to assess risk of bias due to missing results in a synthesis (arising from reporting biases). | 13-14 |
| Certainty assessment | 15 | Describe any methods used to assess certainty (or confidence) in the body of evidence for an outcome. | 13-14 |
| **RESULTS** | | |  |
| Study selection | 16a | Describe the results of the search and selection process, from the number of records identified in the search to the number of studies included in the review, ideally using a flow diagram. | 15, Figure 1 |
|  | 16b | Cite studies that might appear to meet the inclusion criteria, but which were excluded, and explain why they were excluded. | 15, Supplementary Material Table S3 |
| Study characteristics | 17 | Cite each included study and present its characteristics. | 15, Supplementary Material Table S4 |
| Risk of bias in studies | 18 | Present assessments of risk of bias for each included study. | 16, Supplementary Material Table S7 |
| Results of individual studies | 19 | For all outcomes, present, for each study: (a) summary statistics for each group (where appropriate) and (b) an effect estimate and its precision (e.g. confidence/credible interval), ideally using structured tables or plots. | 15-21, Figure 2-5, Supplementary Material S8-13 |
| Results of syntheses | 20a | For each synthesis, briefly summarise the characteristics and risk of bias among contributing studies. | 15-21, Table 1, Supplementary Material Table S7 |
|  | 20b | Present results of all statistical syntheses conducted. If meta-analysis was done, present for each the summary estimate and its precision (e.g. confidence/credible interval) and measures of statistical heterogeneity. If comparing groups, describe the direction of the effect. | 15-21, Figure 2-5, Supplementary Material S8-13 |
|  | 20c | Present results of all investigations of possible causes of heterogeneity among study results. | 15-21, Supplementary Material S8-13 |
|  | 20d | Present results of all sensitivity analyses conducted to assess the robustness of the synthesized results. | 22, Supplementary Material Table S12 |
| Reporting biases | 21 | Present assessments of risk of bias due to missing results (arising from reporting biases) for each synthesis assessed. | 21, Supplementary Material S13 |
| Certainty of evidence | 22 | Present assessments of certainty (or confidence) in the body of evidence for each outcome assessed. | 15-21, Figure 2-5, Supplementary Material S8-13 |
| **DISCUSSION** | | |  |
| Discussion | 23a | Provide a general interpretation of the results in the context of other evidence. | 23-25 |
|  | 23b | Discuss any limitations of the evidence included in the review. | 25-26 |
|  | 23c | Discuss any limitations of the review processes used. | 26-28 |
|  | 23d | Discuss implications of the results for practice, policy, and future research. | 25-26 |
| **OTHER INFORMATION** | | |  |
| Registration and protocol | 24a | Provide registration information for the review, including register name and registration number, or state that the review was not registered. | 8 |
|  | 24b | Indicate where the review protocol can be accessed, or state that a protocol was not prepared. | 8 |
|  | 24c | Describe and explain any amendments to information provided at registration or in the protocol. | 8 |
| Support | 25 | Describe sources of financial or non-financial support for the review, and the role of the funders or sponsors in the review. | 2 |
| Competing interests | 26 | Declare any competing interests of review authors. | 2 |
| Availability of data, code and other materials | 27 | Report which of the following are publicly available and where they can be found: template data collection forms; data extracted from included studies; data used for all analyses; analytic code; any other materials used in the review. | 3 |

# Search Strategy

**Table S2A.** Database search strategy for MEDLINE via OVID

Database: Ovid MEDLINE(R) and Epub Ahead of Print, In-Process, In-Data-Review & Other Non-Indexed Citations, Daily and Versions (1946 to August 02, 2024)

| **#** | **Search term** | **Results** |
| --- | --- | --- |
| 1 | exp PCSK9/ or exp Proprotein Convertase 9/ or exp Proprotein Convertase, Subtilisin Kexin Type 9/ | 3612 |
| 2 | exp PCSK9i/ or exp PCSK9 inhibitors/ | 1278 |
| 3 | PCSK*.mp. | 7250 |
| 4 | (Alirocumab or praluent).mp. | 1022 |
| 5 | (Evolocumab or AMG-145 or repatha).mp. | 1183 |
| 6 | (Inclisiran or ALN-60212 or leqvio).mp. | 385 |
| 7 | exp medication adherence/ or exp drug adherence/ | 26878 |
| 8 | exp patient compliance/ or exp patient adherence/ | 87416 |
| 9 | exp treatment adherence/ or exp treatment compliance/ | 282814 |
| 10 | exp compliance/ | 4076 |
| 11 | Adhere*.mp. | 291896 |
| 12 | Compliance.mp. | 199455 |
| 13 | Persistence.mp. | 117263 |
| 14 | (Initiat* or start*).mp. | 1338347 |
| 15 | Implement*.mp. | 776379 |
| 16 | (Discontinu* or stop*).mp. | 321312 |
| 17 | or/1-6 | 7801 |
| 18 | or/7-16 | 2938231 |
| 19 | 17 and 18 |  |

**Table S2B.** Database search strategy for EMBASE via OVID

Database: Embase (1974 to August 02, 2024)

| **#** | **Search term** | **Results** |
| --- | --- | --- |
| 1 | exp PCSK9/ or exp Proprotein Convertase 9/ or exp Proprotein Convertase, Subtilisin Kexin Type 9/ | 7237 |
| 2 | exp PCSK9i/ or exp PCSK9 inhibitors/ | 6273 |
| 3 | PCSK*.mp. | 11593 |
| 4 | (Alirocumab or praluent).mp. | 3039 |
| 5 | (Evolocumab or AMG-145 or repatha).mp. | 3794 |
| 6 | (Inclisiran or ALN-60212 or leqvio).mp. | 1073 |
| 7 | exp medication adherence/ or exp drug adherence/ | 51279 |
| 8 | exp patient compliance/ or exp patient adherence/ | 203564 |
| 9 | exp treatment adherence/ or exp treatment compliance/ | 203564 |
| 10 | exp compliance/ | 36803 |
| 11 | Adhere*.mp. | 397770 |
| 12 | Compliance.mp. | 432111 |
| 13 | Persistence.mp. | 154077 |
| 14 | (Initiat* or start*).mp. | 2062090 |
| 15 | Implement*.mp. | 1003836 |
| 16 | (Discontinu* or stop*).mp. | 526413 |
| 17 | or/1-6 | 15308 |
| 18 | or/7-16 | 4050734 |
| 19 | 17 and 18 | 2732 |

**Table S2C.** Database search strategy for PsychInfo (ProQuest)

Database: PsychInfo (Inception to August 02, 2024)

| **#** | **Search term** | **Results** |
| --- | --- | --- |
| 1 | PCSK9 OR Proprotein Convertase OR PCSK9 inhibitors OR Alirocumab OR Evolocumab OR Inclisiran | 74 |

**Table S2C.** Database search strategy for CINAHL Plus (EBSCOHost)

Database: CINAHL Plus (Inception to August, 2024)

| **#** | **Search term** | **Results** |
| --- | --- | --- |
| 1 | PCSK9 OR Proprotein Convertase OR PCSK9 inhibitors OR Alirocumab OR Evolocumab OR Inclisiran | 164 |

**Table S2D.** Database search strategy for medRxiv

Database: medRxiv (Inception to August 02, 2024)

| **#** | **Search term** | **Results** |
| --- | --- | --- |
| 1 | PCSK9 OR Proprotein Convertase OR Alirocumab OR Evolocumab OR Inclisiran | 444 |

# List of excluded studies and exclusion reasons

**Table S3A.** Code list of study exclusion criteria to be assessed in hierarchical order during full text screening

| **Order** | **Description** | **Examples** |
| --- | --- | --- |
| 1 | Wrong intervention | - Study is of a non-clinically available PCSK9 inhibitor - Study is of lipid-modifying drugs that are not PCSK9 inhibitor (i.e., alirocumab, evolocumab and inclisiran) |
| 2 | Wrong study design | - Randomized controlled trial - Case reports or case-series - Modelling study |
| 3 | Wrong outcome | - Medication initiation, implementation or persistence is not reported |
| 4 | Wrong publication type | - Systematic reviews - Narrative reviews - Brief reports such as conference abstracts, letters to the editor, correspondence (and similar) if the methods of assessing medication adherence are inadequately described - Protocol only with no results reported - Ongoing study with no results published - Premature termination of study with no outcomes reported |
| 5 | Wrong language | - Study not reported in English |
| 6 | Animal study | - In-vivo study in animals or laboratory studies in cells or tissues |
| 7 | Unable to obtain full text of paper | - Only abstract available from database and full text cannot be obtained for review |

**Table S3B.** List of excluded studies and reasons for exclusion after full text review

| **#** | **Citation** | **Exclusion reason** |
| --- | --- | --- |
| 1 | Abud A, Ziajka P. Effects of concomitant lipid modifying therapy on the LDL-C lowering Efficacy of PCSK-9 inhibition. Journal of Clinical Lipidology. 2017;11(3):838-9. | 3: Wrong outcome |
| 2 | Aguilar E, Dani L, Moya Carmona I, Estaun C, Fernandez Ovies JM. Assessement of efficacy of proprotein convertase subtilisin/kexin type 9 inhibitors (IPCSK9) for hypercholesterolaemia with or without statins. European Journal of Hospital Pharmacy. 2018;25(Supplement 1):A230. | 3: Wrong outcome |
| 3 | Alonso Martinez C, Sanchez-Sanchez P, Larrosa-Garcia M, Garcia-Garcia S, Miarons-Font M, Gomez-Domingo M, et al. Alirocumab and evolocumab: Effectiveness after 3 years of follow-up in a real world setting. European Journal of Hospital Pharmacy. 2020;27(SUPPL 1):A55-A6. | 3: Wrong outcome |
| 4 | Altomari A, Sanga V, Ceradini G, Fabris A, Lupo A, Bonora E, et al. Preliminary clinical experience of PCSK9 inhibitors in patients with heterozygous familial hypercholesterolemia. considerations on their use in pati-ENTS on lipoprotein apheresis treatment and in statins intolerant patients. Nutrition, Metabolism and Cardiovascular Diseases. 2017;27(1):e4-e5. | 3: Wrong outcome |
| 5 | Anastasiou G, Liamis G, Milionis H, Elisaf M, Christopoulou E, Dimitriou T, et al. PCSK9 inhibitors: The breakthrough lipid-lowering treatment at real-life setting. A 3-year regional lipid clinic experience. *Atherosclerosis*. 2021;331((Anastasiou, Liamis, Milionis, Elisaf, Christopoulou, Dimitriou, Liberopoulos) University Hospital of Ioannina, Internal Medicine, Ioannina, Greece):e134. | 4: Wrong publication type |
| 6 | Andersen KM, Ng DK, Blaha MJ, Segal JB, Alexander GC. Longitudinal LDL changes among proprotein convertase subtilisin kexin type 9 inhibitor (PCSK9i) users in the United States. Pharmacoepidemiology and Drug Safety. 2020;29(SUPPL 3):138. | 3: Wrong outcome |
| 7 | Andretta M, Menti AM, Polini M, Degli EL, Bilato C, Zambon A, et al. PCSK9-inhibitors: Identification of eligible patients from regional administrative healthcare databases. Value in Health. 2016;19(7):A659. | 1: Wrong intervention |
| 8 | Antonio Aurelio Rojas Sanchez AA, Mora Robles J, Aviles Toscano AL, Navarrete Espinosa I, Guardia Martinez P, Prieto Toro D, et al. Alirucumab in cardiac rehabilitation patients. Clinical profile and initial results. European Journal of Preventive Cardiology. 2018;25(2 Supplement 1):S147. | 3: Wrong outcome |
| 9 | Aretio A, Campos-Baeta Y, Feliu A, Masip M, Mangues MA. Management of lipid-lowering therapy in PCSK9 inhibitors-treated patients. International Journal of Clinical Pharmacy. 2018;40(1):247-8. | 3: Wrong outcome |
| 10 | Austin C, Kioussopoulos K, Kovacich DO, Chugh AR. Use of PCSK9 inhibitors at a dedicated cardiology-based lipid clinic: A real world cohort. Clinical Cardiology. 2018;41(Supplement 1):5. | 3: Wrong outcome |
| 11 | Avlasevich V, Pilat S, Reindel K, Manou K, Trawinski A, Rightmier E. Optimal follow-up schedule for patients taking PCSK9 monoclonal antibodies in a health system: Analysis of specialty pharmacy clinical interventions. American Journal of Health-System Pharmacy. 2024;81(13):e358-e64. | 3: Wrong outcome |
| 12 | Baez Gutierrez N, Romero Lara C, Rodriguez Ramallo H, Santos Rubio MD, Seisdedos Elcuaz R. Effectiveness and safety of alirocumab in a third level hospital. European Journal of Clinical Pharmacology. 2019;75(Supplement 1):S24-S5. | 4: Wrong publication type |
| 13 | Ballantyne CM, Varisco T, Graham T, Iteld BJ, Serota H, McElligott S, et al. Reductions in LDL-C within the first year of treatment with inclisiran: Results from a multicenter real world cohort. Journal of the American College of Cardiology. 2024;83(13 Supplement):1941. | 4: Wrong publication type |
| 14 | Banach M, Lopez-Sendon JL, Averna M, Cariou B, Loy M, Manvelian G, et al. Treatment adherence and effect of concurrent statin intensity on the efficacy and safety of alirocumab in a real-life setting: Results from ODYSSEY APPRISE. Archives of Medical Science. 2022;18(2):285-92. | 2: Wrong study design |
| 15 | Banga S, Pendyala S, Kizhakekuttu TJ. To compare the "real world" efficacy of proprotein convertase subtilisin-kexin type 9 (PCSK-9) inhibitors alirocumab versus evolocumab on LDL lowering in patients presenting with dyslipidemia: A single center experience. Cardiology (Switzerland). 2018;140(Supplement 1):116. | 3: Wrong outcome |
| 16 | Barrios V, Escobar C, Arrarte V, Bravo M, del Campo A, Hidalgo R, Recasens L, Cequier A. Analysis of the prescription process of PCSK9 inhibitors in the cardiology departments of Spanish hospitals and optimization proposal. The IKIGAI study. Clinica e Investigacion en Arteriosclerosis. 2021;33(6):296-305. | 3: Wrong outcome |
| 17 | Bashir B, Haslam S, Ahmad S, Elnaggar MN, Allcock R, Ali S, et al. Protein convertase subtilisin/kexin type 9 monoclonal antibodies (PCSK9mab) in clinical practice at secondary care - real world multicentre experience. Cureus. 2022;14(12):e33044. | 3: Wrong outcome |
| 18 | Basiak M, Kosowski M, Hachula M, Okopien B. Impact of PCSK9 inhibition on proinflammatory cytokines and matrix metalloproteinases release in patients with mixed hyperlipidemia and vulnerable atherosclerotic plaque. Pharmaceuticals. 2022;15(7):802. | 2: Wrong study design |
| 19 | Basile C, Gargiulo P, Marzano F, Catalano A, Musumeci G, Galasso G, et al. Fast track use of PCSK9i in acute coronary syndromes in real world practice. Insights from the AT-TARGET-IT registry. Circulation. 2023;148(Supplement 1). | 3: Wrong outcome |
| 20 | Bates M, Sridharan M, Betz Y, McClean K, Perry A, Thomas MJ, et al. Rapid, cost-free inclisiran access: A real-world, single centre experience. Journal of the American College of Cardiology. 2024;83(13 Supplement):2011. | 4: Wrong publication type |
| 21 | Baum S, Wade R, Harrison D, Xiang P, Patel J, Nunna S, et al. Cardiovascular risk in patients prescribed proprotein convertase subtilisin/kexin type 9 inhibitor therapy and patients newly initiating statins or changing other lipid-lowering therapy. Journal of Managed Care and Specialty Pharmacy. 2018;24(10 A):S59-S60. | 3: Wrong outcome |
| 22 | Bellato N, Campanardi MC, Calzavara E, Nobili S, De Vivo G, Cambareri M, et al. Evolocumab real-world data: Five-year experience of an Italian hospital. International Journal of Clinical Pharmacy. 2023;45(3):791. | 3: Wrong outcome |
| 23 | Bertani G, Mugnolo A, Venturi G, Bacchion F, Morando G, Zamboni A. Small interfering RNA: Real-life data with a new frontier in hypolipidemic therapy. European Heart Journal, Supplement. 2023;25(Supplement D):D185-D6. | 3: Wrong outcome |
| 24 | Bertran de lis Bartolome B, Gonzalez Sevilla M, Ferrari Piquero JM. Adherence to evolocumab and its impact on LDL cholesterol reduction. European Journal of Hospital Pharmacy. 2023;30(Supplement 1):A31-A2. | 3: Wrong outcome |
| 25 | Bigness C, Tungol Lin A. Evaluation of prior authorization requests for alirocumab and evolocumab at a PPO health plan. Journal of Managed Care and Specialty Pharmacy. 2016;22(10-A SUPPL.):S42. | 3: Wrong outcome |
| 26 | Bizier J, Stryker M, Kane M. Extended-use, proprotein convertase subtilisin/kexin type 9 Inhibitor (PCSK9i) data in patients with diabetes mellitus: A pharmacist-led initiative. Journal of Clinical Lipidology. 2017;11(3):778. | 3: Wrong outcome |
| 27 | Bonafede M, Lopez-Gonzalez L, Rane P, Patel J, Harrison D. Clinical characteristics of early adopters of proprotein convertase subtilisin/ kexin type 9 inhibitors (PCSK9I). Journal of the American College of Cardiology. 2017;69(11 Supplement 1):1869. | 3: Wrong outcome |
| 28 | Boytsov SA, Shakhnovich RM, Tereschenko SN, Erlikh AD, Kukava NG, Pevsner DV, et al. The prevalence of hyperlipidemia and features of lipid-lowering therapy in patients with myocardial infarction according to the Russian register of acute myocardial infarction REGION-MI. Kardiologiia. 2022;62(7):12-22. | 3: Wrong outcome |
| 29 | Brambilla A, Venezia S, Latella M, Garavaglia A, Grillo E. New era in hypercholesterolemia treatment, inclisiran: Early and sustained LDL-C reduction with a twice per year administration. European Heart Journal, Supplement. 2021;23(SUPPL G):G35. | 2: Wrong study design |
| 30 | Brandt E. The effect of PCSK9 inhibition on sterol absorption in a cohort of real world patients. Journal of the American College of Cardiology. 2017;69(11 Supplement 1):1715. | 3: Wrong outcome |
| 31 | Brandt EJ, Benes LB, Lee L, Dayspring TD, Sorrentino M, Davidson M. The effect of proprotein convertase subtilisin/kexin type 9 inhibition on sterol absorption markers in a cohort of real-world patients. Journal of Cardiovascular Pharmacology and Therapeutics. 2019;24(1):54-61. | 3: Wrong outcome |
| 32 | Brinar IV, Ivandic E, Gellineo L, Jelakovic A, Prlic MF, Jelakovic B. PCSK9 inhibitors are efficient in treatment of resistant hypercholesterolemia in nephrotic syndrome. Nephrology Dialysis Transplantation. 2022;37(SUPPL 3):i167 | 4: Wrong publication type |
| 33 | Browne C, Neves E, Breen J, Davies L, Priestley-Barnham L, Pottle A, et al. Inject away: The Harefield experience on delivering the new PCSK9 inhibitors. Atherosclerosis Supplements. 2017;28((Browne, Neves, Breen, Davies, Priestley-Barnham, Pottle, Hayes, Thompson, Barbir) Royal Brompton and Harefield NHS Foundation Trust, Harefield Hospital, Hill End Road, Harefield UB9 6JH, United Kingdom):e12-e3. | 4: Wrong publication type |
| 34 | Buonocore D, Basile C, Calabro P, Marzano F, Asile G, Abbate V, et al. Safety of PCSK9i in a single country, multicenter, observational study: A post-hoc analysis of the AT-TARGET-IT study. European Heart Journal, Supplement. 2022;24(Supplement K):K250. | 3: Wrong outcome |
| 35 | Calcaterra I, Di Minno A, Orsini R, Chiesa M, Cavalca V, Tripaldella M, et al. Treatment with PCSK9 inhibitors in patients with familial hypercholesterolemia lowers plasma levels of platelet activating factor and its precursors: A combined metabolomic and lipidomic approach. Research and Practice in Thrombosis and Haemostasis. 2023;7(Supplement 2):100925. | 3: Wrong outcome |
| 36 | Capps N. Impact of PCSK9 inhibitors alirocumab and evolocumab on total & LDL cholesterol in clinical practice. Atherosclerosis Supplements. 2017;28((Capps) Department of Clinical Biochemistry, Shrewsbury and Telford Hospital NHS Trust, Princess Royal Hospital, Telford, United Kingdom):e13. | 3: Wrong outcome |
| 37 | Catapano AL, Maggioni AP, Rossi F, Tirone G, Notarianni L, Agnelli G, et al. Observational multicentre study on effectiveness and tolerability of alirocumab in real world, the OMERO study: Interim data from the first 699 patients. European Heart Journal, Supplement. 2021;23(SUPPL G):G193-G4. | 3: Wrong outcome |
| 38 | Cavarra M, Salerno E, Chiaranda G. Alirocumab effectiveness and rapidity in achieving and maintaining target LDL, HDL cholesterol and triglyceride values in patients at very high cardiovascular risk. European Journal of Preventive Cardiology. 2021;28(SUPPL 1):i375. | 3: Wrong outcome |
| 39 | Cavarra M, Salerno E, Chiaranda M, Chiaranda G, Tamburino C. Alirocumab effectiveness and rapidity in achieving and maintaining target LDL cholesterol values in patients at very high cardiovascular risk, also assessing its effects on triglyceride and HDL cholesterol values. European Heart Journal, Supplement. 2023;25(Supplement D):D234 | 3: Wrong outcome |
| 40 | Cesaro A, Gragnano F, Fimiani F, Moscarella E, Diana V, Pariggiano I, et al. Quality of life assessment in patients treated with PCSK9 inhibitors. European Heart Journal, Supplement. 2019;21(SUPPL J):J197. | 3: Wrong outcome |
| 41 | Cesaro A, Gragnano F, Fimiani F, Moscarella E, Pariggiano I, Diana V, et al. Quality of life improvement in high and very high cardiovascular risk patients treated with PCSK9 inhibitors. Atherosclerosis. 2019;287((Cesaro, Gragnano, Fimiani, Moscarella, Pariggiano, Diana, Carfora, Conte, Falato, Cesarano, Di Maio, Calabro) University of Campania "L. Vanvitelli", Department of Medical Translational Sciences, Naples, Italy):e199. | 3: Wrong outcome |
| 42 | Choi J, Khan AM, Jarmin M, Goldenberg N, Glueck CJ, Wang P. Efficacy and safety of proprotein convertase subtilisin-kexin type 9 (PCSK9) inhibitors, alirocumab and evolocumab, a post-commercialization study. Lipids in Health and Disease. 2017;16(1):141. | 3: Wrong outcome |
| 43 | Chubykina U, Ezhov M, Shaposhnik I, Genkel V, Ershova A, Gurevich V, et al. Compliance of heterozygous familial hypercholesterolemia patients: 5-years follow-up of the Russian familial hypercholesterolemia registry. Atherosclerosis. 2023;379(Supplement 1):S84. | 1: Wrong intervention |
| 44 | Clemente Lorenzo MM, Saez Jimenez SA, Gervas Pabon H, Temprano Ferreras JL, Penalver Talavera D. Difficult-to-treat hypercholesterolemia. Early experience with PCSK9-inhibitors in real world practice in a local hospital. Global Heart. 2018;13(4):433-4. | 4: Wrong publication type |
| 45 | Collados Arroyo V, Fernandez-Caballero R, Henares Lopez A, Mayo Lopez C. Budgetary impact of PCSK9i doses regimen optimisation. European Journal of Hospital Pharmacy. 2022;29(SUPPL 1):A82-A3. | 4: Wrong publication type |
| 46 | Collins M, Weber B, Dajani K. Side effect profile in PCSK9 inhibitors. Clinical Cardiology. 2019;42(Supplement 2):S44-S5. | 3: Wrong outcome |
| 47 | Colvin CL, Poudel B, Bress AP, Derington CG, King JB, Wen Y, et al. Race/ethnic and sex differences in the initiation of non-statin lipid-lowering medication following myocardial infarction. Journal of Clinical Lipidology. 2021;15(5):665-73. | 3: Wrong outcome |
| 48 | Cordero A, Fernandez Del Olmo MR, Cortez Quiroga GA, Romero-Menor C, Facila L, Seijas-Amigo J, et al. Sex differences in low-density lipoprotein cholesterol reduction with PCSK9 Inhibitors in real-world patients: The LIPID-REAL Registry. Journal of Cardiovascular Pharmacology. 2022;79(4):523-9. | 3: Wrong outcome |
| 49 | Cordero A, Fernandez Olmo MR, Cortez Quiroga GA, Romero-Menor C, Facila L, Seijas-Amigo J, et al. Effect of PCSK9 inhibitors on remnant cholesterol and lipid residual risk: The LIPID-REAL registry. European Journal of Clinical Investigation. 2022;52(12):e13863. | 3: Wrong outcome |
| 50 | Cortizo ENG, Rodriguez EM, Carrasco AC, Ruiz-Granados ES, Estruch EB, Vazquez JCA, et al. Safety and effectiveness of triple hypolipemian therapy in the light of the new European dyslipidaemia guidelines. Atherosclerosis. 2020;315((Cortizo, Rodriguez, Carrasco, Ruiz-Granados, Vazquez, Jimenez) Infanta Elena Hospital, Internal Medicine, Huelva, Spain(Estruch) Infanta Elena Hospital, Clinical Analysis Laboratory, Huelva, Spain):e107. | 3: Wrong outcome |
| 51 | D'Souza K, Rothschild M, Jetty V, Anaba U, Min S, Goldenberg N, et al. Efficacy, safety, LDL cholesterol lowering, and 10-year ascvd risk reduction: Alirocumab and evolocumab in addition to maximal tolerated LDL lowering. Journal of Clinical Lipidology. 2017;11(3):802. | 3: Wrong outcome |
| 52 | Dal Pino B, Sbrana F. Therapeutic adherence in patients treated with PCSK9i: Focus on skin side effects. Acta Cardiologica. 2024. | 2: Wrong study design |
| 53 | Dayoub EJ, Eberly LA, Nathan AS, Khatana SAM, Adusumalli S, Navar AM, et al. Adoption of PCSK9 inhibitors among patients with atherosclerotic disease. Journal of the American Heart Association. 2021;10(9):e019331. | 3: Wrong outcome |
| 54 | Dayoub EJ, Nathan AS, Adusumalli S, Groeneveld PW. PCSK9 inhibitor use and health characteristics among high-risk patients with atherosclerotic cardiovascular disease. Circulation: Cardiovascular Quality and Outcomes. 2019;12(SUPPL 1). | 3: Wrong outcome |
| 55 | de Carvalho LSF, Yoshida H. Monthly PCSK9 inhibitors: The CHOICE for prolonged duration of effect. Atherosclerosis. 2016;254((de Carvalho) Cardiology Department, State University of Campinas (Unicamp), Campinas, SP, Brazil(Yoshida) Department of Laboratory Medicine, Jikei University Kashiwa Hospital, Chiba, Japan):300-2. | 2: Wrong study design |
| 56 | Delnevo F, Annibali G, De Rosa C, Muccioli S, Civera S, Colopi M, et al. A real world single center experience with early use of triple lipid lowering therapy. Atherosclerosis. 2023;379(Supplement 1):S196. | 4: Wrong publication type |
| 57 | Demichelis B, Giammaria M, Annibali G, De Rosa C, Muccioli S, Fasano R, et al. A real-world protocol of early use of triple lipid lowering therapy with I-PCSK9. European Heart Journal, Supplement. 2024;26(Supplement 2):ii77-ii8. | 3: Wrong outcome |
| 58 | Deo S, McAllister D, Laforest SK, Bal S, Altarabsheh S, Elgudin Y, et al. Disparities in PCSK9 inhibitor initiation among patients with peripheral arterial disease or cerebrovascular disease: A cohort study of 500,000 patients with atherosclerotic vascular disease. Circulation: Cardiovascular Quality and Outcomes. 2022;15(Supplement 1). | 3: Wrong outcome |
| 59 | Deo SV, McAllister D, LaForest S, Altarabsheh S, Elgudin YE, Dunlay S, et al. Disparities in PCSK9 initiation among us veterans with peripheral arterial disease or cerebrovascular disease. American Journal of Cardiovascular Drugs. 2023;23(3):311-21. | 3: Wrong outcome |
| 60 | Derington CG, Colantonio LD, Herrick JS, Cook J, King JB, Rosenson RS, et al. Factors associated with PCSK9 inhibitor initiation among US Veterans. Journal of the American Heart Association. 2021;10(8):e019254. | 2: Wrong study design |
| 61 | Desai N, Rane P, Nunna S, Chen CC, Exter J, Habib M, et al. Lipid lowering treatment patterns and risk of subsequent cardiovascular outcomes among patients initially rejected for PCSK9 inhibitor therapy. Journal of Clinical Lipidology. 2020;14(4):599-600. | 3: Wrong outcome |
| 62 | Desai N, Xiang P, Exter J, Habib M, Wang X, Nunna S, et al. Low-density lipoprotein-cholesterol lowering in patients with a recent myocardial infarction and subsequent treatment with evolocumab in real-world clinical practice. Journal of Clinical Lipidology. 2020;14(4):551-2. | 3: Wrong outcome |
| 63 | Desai NR, Wade RL, Xiang P, Nunna S, Wang X, Exter J, et al. Low-density lipoprotein-cholesterol lowering in real-world patients treated with evolocumab. Circulation. 2019;140(Supplement 1). | 3: Wrong outcome |
| 64 | Di Minno MND, Gentile M, Di Minno A, Iannuzzo G, Calcaterra I, Buonaiuto A, et al. Changes in carotid stiffness in patients with familial hypercholesterolemia treated with evolocumab: A prospective cohort study. Nutrition, metabolism, and cardiovascular diseases : NMCD. 2020;30(6):996-1004. | 3: Wrong outcome |
| 65 | Dufour R, Bergeron J, Gaudet D, Weiss R, Hovingh GK, Qing Z, et al. Open-label therapy with alirocumab in patients with heterozygous familial hypercholesterolemia: Results from three years of treatment. International Journal of Cardiology. 2017;228((Dufour) Institut de recherches cliniques de Montreal and Universite de Montreal, Montreal, QC, Canada(Bergeron) Clinique des Maladies Lipidiques, Centre Hospitalier Universitaire de Quebec-Universite Laval, Quebec, QC, Canada(Gaudet) ECOGENE-21 Clinical):754-60. | 2: Wrong study design |
| 66 | Dufour R, Hovingh GK, Guyton JR, Langslet G, Baccara-Dinet MT, Din-Bell C, et al. Individualized low-density lipoprotein cholesterol reduction with alirocumab titration strategy in heterozygous familial hypercholesterolemia: Results from an open-label extension of the ODYSSEY LONG TERM trial. Journal of Clinical Lipidology. 2019;13(1):138-47. | 2: Wrong study design |
| 67 | Ezhov MV, Tmoyan NA, Rozhkova TA, Duplyakov DV, Salchenko VA, Kachkovskii MA, et al. Adherence and efficiency of lipid-lowering therapy in patients with heterozygous familial hypercholesterolemia in Russia. Atherosclerosis. 2020;315((Ezhov, Tmoyan, Chubykina) National Medical Research Center of Cardiology, Laboratory Of Lipid Disorders, Moscow, Russian Federation(Rozhkova) National Medical Research Center of Cardiology, Department Of Atherosclerosis, moscow, Russian Federation(Duplya):e43. | 3: Wrong outcome |
| 68 | Fages Perez M, Romero Puerto J, Soria Martin A. Efficacy and adequacy of evolocumab in hypercholesterolemia. International Journal of Clinical Pharmacy. 2020;42(1):282. | 3: Wrong outcome |
| 69 | Farnier M, Guyton J, Langslet G, Dufour R, Baccara-Dinet M, Din-Bell C, et al. On-treatment LDL-C levels when alirocumab dose is decreased from 150 to 75 mg every 2 weeks in patients with heterozygous familial hypercholesterolemia: Results from ODYSSEY. Canadian Journal of Cardiology. 2017;33(10 Supplement 1):S115. | 2: Wrong study design |
| 70 | Farnier M, Hovingh GK, Langslet G, Dufour R, Baccara-Dinet M, Din-Bell C, et al. Durability of alirocumab effect: Data from an open-label extension to the ODYSSEY program for patients with heterozygous familial hypercholesterolemia. Journal of Clinical Lipidology. 2017;11(3):840. | 2: Wrong study design |
| 71 | Farnier MA, Hovingh GK, Langslet G, Dufour R, Baccara-Dinet MT, Din-Bell C, et al. High level of adherence to alirocumab and concomitant background treatments for patients with heterozygous familial hypercholesterolemia in the ODYSSEY open-label extension study. Circulation. 2017;136(Supplement 1). | 2: Wrong study design |
| 72 | Feng X, Berklein F, Rane PB, Habib M, Lin PJ. Patient characteristics and treatment patterns among medicare beneficiaries initiating PCSK9 inhibitor therapy. Cardiovascular Drugs and Therapy. 2021;35(5):965-73. Feng X, Berklein F, Rane PB, Habib M, Lin PJ. Patient characteristics and treatment patterns among medicare beneficiaries initiating PCSK9 inhibitor therapy. Cardiovascular Drugs and Therapy. 2021;35(5):965-73. | 3: Wrong outcome |
| 73 | Gabaldon Garnica P, Sobrino Jimenez C, Moreno Ramos F, Gonzalez del Valle L, Jimenez Vicente C, Herrero Ambrosio A. Adherence and effectiveness of PCSK9 inhibitors in routine clinical practice. European Journal of Hospital Pharmacy. 2019;26(Supplement 1):A79-A80. | 4: Wrong publication type |
| 74 | Gagliardi A, Palleria C, Naturale MD, De Francesco AE, Leporini C, Muraca L, et al. Safety profile of PCSK9 inhibitors in clinical practice: An Italian prospective pharmacovigilance study. Drug Safety. 2022;45(10):1150-1. | 3: Wrong outcome |
| 75 | Galema-Boers A, Mulder J, Steward K, Van Lennep JR. First clinical experiences with inclisiran in real world setting. Atherosclerosis. 2023;379(Supplement 1):S166. | 3: Wrong outcome |
| 76 | Gandhi SK, Kumar M, Wu C, Kralova K, Juhaeri J. Use of lipid-lowering treatments in patients infected with human immunodeficiency virus. Pharmacoepidemiology and Drug Safety. 2017;26(Supplement 2):115-6. | 3: Wrong outcome |
| 77 | Glueck CJ, Brown A, Goldberg A, McKenney J, Kantaros L, Stewart J, et al. Alirocumab in high-risk patients with baseline LDL-C >=160 mg/dL: Findings from the compassionate use program. Journal of the American College of Cardiology. 2017;69(11 Supplement 1):155. | 2: Wrong study design |
| 78 | Glueck CJ, Brown A, Goldberg AC, McKenney JM, Kantaros L, Stewart J, et al. Alirocumab in high-risk patients: Observations from the open-label expanded use program. Journal of Clinical Lipidology. 2018;12(3):662-8. | 2: Wrong study design |
| 79 | Goldberg A, Wooten M, Dunbar R, Hemphill L, Babirak S, Wilson G, et al. Retrospective review of the effect of alirocumab on apheresis. Journal of Clinical Lipidology. 2017;11(3):834-5. | 3: Wrong outcome |
| 80 | Goldberg AC, Dunbar RL, Hemphill L, Babirak SP, Wilson G, Wooten M, et al. A retrospective analysis of clinical use of alirocumab in lipoprotein apheresis patients. Journal of Clinical Lipidology. 2020;14(6):818-24. | 3: Wrong outcome |
| 81 | Gomez Zamora M, Rovira Torres P, Jimenez Portilla A, Ortiz M, Gomez Lobon A, Santandreu Esterlich MM, et al. Real-world effectiveness of evolocumab and alirocumab at 12 months of treatment. European Journal of Hospital Pharmacy. 2019;26(Supplement 1):A80. | 3: Wrong outcome |
| 82 | Gonzalez-Bustos P, Bustos-Merlo A, Roa-Chamorro R, Jaen-Aguila F, Mediavilla-Garcia JD. Safety, adherence and efficacy profile of alirocumab after one year of treatment. Atherosclerosis. 2021;331((Gonzalez-Bustos, Bustos-Merlo, Roa-Chamorro, Jaen-Aguila, Mediavilla-Garcia) Hospital Universitario Virgen de las Nieves. Granada, Internal Medicine, Granada, Spain):e261-e2. | 4: Wrong publication type |
| 83 | Gouni-Berthold I, Seshagiri D, Studer R, Durand A, Klebs S, Achouba A, et al. Treatment patterns in patients with familial hypercholesterolemia: Evidence from real-world studies in Germany and the UK. European Heart Journal. 2021;42(SUPPL 1):2570. | 4: Wrong publication type |
| 84 | Gouni-Berthold I, Seshagiri D, Studer R, Klebs S, Achouba A, Becker EM, et al. A real-world assessment of treatment patterns in patients with atherosclerotic cardiovascular disease with hypercholesterolemia: A retrospective database analysis in Germany. European Heart Journal. 2021;42(SUPPL 1):2571. | 1: Wrong intervention |
| 85 | Graesdal A, Bogsrud M, Johansen D, Dybvig A, Holven K. Real-life PCSK9 experience: 65% treatment target attainment in familial hypercholesterolemia patients. Journal of the American College of Cardiology. 2017;69(11 Supplement 1):1716. | 3: Wrong outcome |
| 86 | Graesdal A, Dybvig A. Real-life PCSK9 experience: Efficacy, compliance and side effects after one year treatment in familial hypercholesterolemia patients. Journal of the American College of Cardiology. 2018;71(11 Supplement 1). | 3: Wrong outcome |
| 87 | Graham TE, Varisco TJ, Ballantyne CM, Iteld BJ, Serota H, McElligott S, et al. Real-world use of inclisiran in outpatient physician clinics. Diabetes. 2023;72(Supplement 1). | 3: Wrong outcome |
| 88 | Guilmot A, Philippart M, Descamps O, Sablon G, Tarantino E. Familial hypercholesterolemia: A real life experience with anti-PSK9 with adult patients in Belgium. Acta Clinica Belgica. 2020;75(Supplement 1):51-2. | 3: Wrong outcome |
| 89 | Guyton JR, Hovingh GK, Langslet G, Dufour R, Baccara-Dinet M, Din-Bell C, et al. Alirocumab treatment in a real world setting: Safety update from an open-label treatment extension to the ODYSSEY program for patients with heterozygous familial hypercholesterolemia. Journal of the American College of Cardiology. 2017;69(11 Supplement 1):1660. | 2: Wrong study design |
| 90 | Harada-Shiba M, Kishimoto I, Makino H, Ogura M, Kurtz C, Honarpour N, et al. Efficacy of evolocumab (AMG 145) in patients with PCSK9 gain-of-function mutations. Atherosclerosis. 2014;235(2):e11. | 2: Wrong study design |
| 91 | Hayes C, Browne C, Neves E, Breen J, Priestley-Barnham L, Davis L, et al. A multi-disciplinary approach to delivering PCSK9 inhibitor therapy - first 12 months experience at a specialist cardiothoracic centre. Heart. 2018;104(Supplement 6):A60-A1. | 3: Wrong outcome |
| 92 | Henry P, Cariou B, Farnier M, Lakhdari SL, Detournay B. Lipid-lowering efficacy and safety of alirocumab in a real-life setting in France: Insights from the ODYSSEY APPRISE study. Archives of Cardiovascular Diseases. 2023;116(1):3-8. | 2: Wrong study design |
| 93 | Hirsch A, Bartsch K, Snider M, Larry J, Morton S, Sharma K. Early experience with proprotein convertase subtilisin/kexin type 9 (PCSK9) inhibitors in patients treated in an outpatient lipid clinic. Journal of Clinical Lipidology. 2016;10(3):716. | 3: Wrong outcome |
| 94 | Hollstein T, Vogt A, Grenkowitz T, Stojakovic T, Marz W, Laufs U, et al. Treatment with PCSK9 inhibitors reduces atherogenic VLDL remnants in a real-world study. Vascular Pharmacology. 2019;116((Hollstein, Grenkowitz, Steinhagen-Thiessen, Kassner) Department of Endocrinology, Campus Virchow-Klinikum, Charite Universitatsmedizin Berlin, Berlin, Germany(Stojakovic, Marz, Scharnagl) Clinical Institute of Medical and Chemical Laboratory Diagnostics):8-15. | 3: Wrong outcome |
| 95 | Hovingh GK, Guyton JR, Langslet G, Dufour R, Baccara-Dinet MT, Din-Bell C, et al. Alirocumab dosing patterns during 40 months of open-label treatment in patients with heterozygous familial hypercholesterolemia. Journal of Clinical Lipidology. 2018;12(6):1463-70. | 2: Wrong study design |
| 96 | Hovingh GK, Guyton JR, Langslet G, Dufour R, Baccara-Dinet MT, Din-Bell C, et al. Alirocumab dosing in a real world setting: Data from an open-label treatment extension to the ODYSSEY program for patients with heterozygous familial hypercholesterolemia. Circulation. 2016;134(Supplement 1). | 2: Wrong study design |
| 97 | Huth G, Mayer A, Collar J. Treating hypercholesterolemia with PCSK9 inhibitors in a real-world setting: Twenty month experience with ninety patients. Journal of Clinical Lipidology. 2017;11(3):839-40. | 3: Wrong outcome |
| 98 | Imarhia FI, Sulaica EM, Varisco T, Pilate M. Evaluation of the effectiveness and safety of alirocumab use in statin-intolerant Veterans. Federal practitioner : for the health care professionals of the VA, DoD, and PHS. 2021;38(Suppl 4):e67-e71. | 2: Wrong study design |
| 99 | Israni A, Jones B, Salem V, Bravis V. Impact of PCSK9 inhibitors on hypercholesterolaemic patients at a tertiary centre lipid clinic. Atherosclerosis. 2021;331((Israni) Imperial College London, Endocrinology, London, United Kingdom(Jones, Salem, Bravis) Imperial College Healthcare NHS Trust, Endocrinology, London, United Kingdom):e176. | 3: Wrong outcome |
| 100 | Jackson R, Jennings D, Gaine M, Fanek T, Mabasa A, Lee J, et al. Use of PCSK9 inhibitor therapy in a small cohort of heart transplant recipients with statin intolerance or refractory hyperlipidemia. Journal of Heart and Lung Transplantation. 2019;38(4 Supplement):S275. | 3: Wrong outcome |
| 101 | Jatem E, Lima J, Montoro B, Torres-Bondia F, Segarra A. Efficacy and safety of PCSK9 inhibitors in hypercholesterolemia associated with refractory nephrotic syndrome. Kidney International Reports. 2021;6(1):101-9. | 2: Wrong study design |
| 102 | Jensen JS, Weeke PE, Bang LE, Hofsten DE, Ripa MS, Schjerning AM, et al. Clinical characteristics and lipid lowering treatment of patients initiated on proprotein convertase subtilisin-kexin type 9 inhibitors: A nationwide cohort study. BMJ Open. 2019;9(4):e022702. | 3: Wrong outcome |
| 103 | Jin ZL, He T, Peng L, Wu XY, Fan D, Chen M, et al. Lipoprotein(a) and benefit of PCSK9 inhibition in emergency complex higher-risk and indicated patients. Current medical science. 2023;43(6):1206-12. | 3: Wrong outcome |
| 104 | Jubran A, Shapira C, Lavie G, Flugelman MY, Halon D, Zafrir B. Clinical features and gaps in the management of familial hypercholesterolemia with prevalent cardiovascular disease. Journal of the American College of Cardiology. 2017;69(11 Supplement 1):1706. | 1: Wrong intervention |
| 105 | Kassner U, Hollstein T, Grenkowitz T, Scharnagl H, Maerz W, Steinhagen-Thiessen E. PCSK9-Inhibitor treatment of cardiovascular high risk patients in a real-world setting. European Heart Journal. 2018;39(Supplement 1):1041. | 3: Wrong outcome |
| 106 | Kibbons A, Peter M, Bell J, Jolly J, Cherry E, Alhashemi B, et al. Identifying nonadherence to specialty medications: Comparing pharmacy claims data and individual reasons for nonadherence. Journal of Managed Care and Specialty Pharmacy. 2020;26(10-A SUPPL.):S75. | 1: Wrong intervention |
| 107 | Kolovou V, Katsiki N, Makrygiannis S, Mavrogieni S, Karampetsou N, Manolis A, et al. Lipoprotein apheresis and proprotein convertase subtilisin/kexin type 9 inhibitors in patients with heterozygous familial hypercholesterolemia: A one center study. Journal of Cardiovascular Pharmacology and Therapeutics. 2021;26(1):51-8. | 3: Wrong outcome |
| 108 | Koprivnik S, Casas Martinez A, Rodriguez Penin I. Initial experience with the use of PCSK9- inhibitors in the real-world clinical practice. European Journal of Hospital Pharmacy. 2018;25(Supplement 1):A58-A9. | 2: Wrong study design |
| 109 | Korneva V, Kuznetsova T, Vezikova N. Four years of application experience IPCSK9. Atherosclerosis. 2023;379(Supplement 1):S167. | 3: Wrong outcome |
| 110 | Korneva VA, Kuznetsova T, Vezikova N. Some social and clinical characteristics of patients on iPCSK9 therapy in karelia republic. Atherosclerosis. 2022;355((Korneva, Kuznetsova, Vezikova) Faculty Therapy, Petrozavodsk State University, PETROZAVODSK, Russian Federation):172. | 3: Wrong outcome |
| 111 | Korneva VA, Kuznetsova TY, Scopetc IS, Vezikova NN. Experience of the use of the PCSK9 inhibitor alirocumab in patients with extremely high cardiovascular risk. Kardiologiia. 2020;60(8):71-7. | 5: Wrong language |
| 112 | Korneva VA, Kuznetsova TY, Scopets IS, Vezikova NN. Efficacy and safety of the use of alirocumab in real clinical practice. Terapevticheskii arkhiv. 2023;94(12):1401-6. | 2: Wrong study design |
| 113 | Kosiborod MN, Arnold SV, Delemos J, Rosenson RS, Ballantyne CM, Liu Y, et al. Use of guideline-recommended risk-reduction strategies among patients with type 2 diabetes and established ASCVD: A 1-year update from getting to an improved understanding of low-density lipoprotein cholesterol and dyslipidemia management (GOULD). Diabetes. 2020;69(Supplement 1). | 3: Wrong outcome |
| 114 | Kosowski M, Basiak M, Hachula M, Okopien B. Impact of alirocumab on release markers of atherosclerotic plaque vulnerability in patients with mixed hyperlipidemia and vulnerable atherosclerotic plaque. Medicina (Kaunas, Lithuania). 2022;58(7). | 2: Wrong study design |
| 115 | Krempf M, Hopkins PN, Bruckert E, Lee S, Donahue S. Efficacy and safety of alirocumab in patients with autosomal dominant hypercholesterolemia associated with proprotein convertase subtilisin/kexin type 9 gain-of-function or apolipoprotein B loss-of-function mutations. American Journal of Cardiology. 2020;125(6):880-6. | 2: Wrong study design |
| 116 | Krempf M, Hopkins PN, Bruckert E, Luc G, Lee S, Donahue S. Pharmacodynamics of alirocumab in patients with autosomal dominant hypercholesterolemia associated with PCSK9 gain-of-function or ApoB loss-of-function mutations: An open-label extension study. Circulation. 2018;138(Supplement 1). | 2: Wrong study design |
| 117 | Kuchis M, Eldeiry A, Busch R, Racz M, Kane M. Real-world long-term safety and efficacy of proprotein convertase subtilisin/kexin type 9 (PCSK9) inhibitors. Endocrine Practice. 2020;26(SUPPL 2):166-7. | 3: Wrong outcome |
| 118 | Kuhl M, Binner C, Jozwiak J, Fischer J, Hahn J, Addas A, et al. Treatment of hypercholesterolaemia with PCSK9 inhibitors in patients after cardiac transplantation. PLoS ONE. 2019;14(1):e0210373. | 3: Wrong outcome |
| 119 | Kuroda K, Iwasaki K, Nakajima S, Watanabe T, Seguchi O, Yanase M, et al. The evaluation of safety and efficacy of PCSK9 inhibitor in patients with cardiac allograft vasculopathy and hypercholesterolemia. Transplantation. 2020;104(SUPPL 3):S601. | 4: Wrong publication type |
| 120 | Laufs U, Birkenfeld AL, Fraass U, Hohenstein B, Siegert C, Klotsche J, et al. Novel insights into the management of German patients with very high cardiovascular risk eligible for PCSK9 inhibitor treatment: Baseline characteristics from the PERI-DYS study. European Heart Journal. 2021;42(SUPPL 1):2562. | 3: Wrong outcome |
| 121 | Lecis M, Viglione E, Strobino S, Ceravolo G. PCSK-9 inhibitors: Real world effectiveness. European Journal of Hospital Pharmacy. 2020;27(SUPPL 1):A157-A8. | 4: Wrong publication type |
| 122 | Lecis M, Viglione E, Strobino S, Ceravolo G, Martinengo S, Rosa A, et al. Effectiveness in the treatment of hypercholesterolemia with PCSK-9 inhibitors. Giornale Italiano di Farmacia Clinica. 2019;33(1):20-8. | 5: Wrong language |
| 123 | Lin PL, Wu YW, Lin CF, Yeh HI, Chang WT, Charng MJ, et al. Real-world analyses of the treatment conditions in patients initiating proprotein convertase subtilisin/kexin type 9 (PCSK9) inhibitor in Taiwan. Journal of Atherosclerosis and Thrombosis. 2023;30(9):1123-31. | 3: Wrong outcome |
| 124 | Locuratolo N, Scicchitano P, Antoncecchi E, Basso P, Bonfantino VM, Brescia F, et al. Follow-up of patients after acute coronary syndrome: The PONTE-SCA Puglia program. Giornale Italiano di Cardiologia. 2022;23(1):63-74. | 7: Unable to obtain full text of paper |
| 125 | Lou B, Liu H, Luo Y, Jiang GT, Wu H, Wang C, et al. In-hospital initiation of PCSK9 inhibitor and short-term lipid control in patients with acute myocardial infarction. Lipids in Health and Disease. 2022;21(1):105. | 3: Wrong outcome |
| 126 | Lugari S, Nascimbeni F, Mondelli A, Bursi S, Onfiani G, Milazzo M, et al. Efficacy and safety of PCSK9 inhibitors: The real-life experience of the lipid clinic in Modena, Italy. Atherosclerosis. 2019;287((Lugari, Nascimbeni, Mondelli, Bursi, Onfiani, Milazzo, Pellegrini, Carubbi) University of Modena and Reggio Emilia, Department of Biomedical- Metabolic and Neural Sciences, Modena, Italy):e200. | 3: Wrong outcome |
| 127 | Luo T, Yuan J, Qiu L, Liu D, Jian X, Hu P, et al. Real-word effectiveness of early start-up and short-term use of PCSK9 inhibitor in the treatment of acute coronary syndrome in China. American Journal of Cardiology. 2023;207((Luo, Yuan, Qiu, Liu, Jian, Hu, Yan, Wang, Yan) Department of Cardiology, Wuhan Asia Heart Hospital Affiliated to Wuhan University of Science and Technology, Wuhan, China):137-9. | 3: Wrong outcome |
| 128 | Madan Raj Poudel MR, Stoyanova D, Gottfried-Kwasniok R, Van Buuren F, Horstkotte D, Mellwig KP. First real world experience with the novel lipid-lowering drug PCSK9 inhibitor evolocumab in patients undergoing lipid apheresis: 1-year follow up. European Journal of Preventive Cardiology. 2018;25(2 Supplement 1):S12. | 3: Wrong outcome |
| 129 | Madan Raj Poudel MR, Stoyanova D, Gottfried-Kwasniok R, Van Buuren F, Horstkotte D, Mellwig KP. Safety aspects and side effects of the novel lipid-lowering PCSK9 inhibitors evolocumab/alirozumab in atherosclerotic patients with familial hypercholesterolemia under lipid apheresis. European Journal of Preventive Cardiology. 2018;25(2 Supplement 1):S37. | 3: Wrong outcome |
| 130 | Maloberti A, Vatta A, Grasso A, Garofani I, Morelli M, Giani V, et al. Effects of PCSK-9 inhbitors on arterial structure and function: Preliminary results. Journal of Hypertension. 2023;41(Supplement 3):e310. | 3: Wrong outcome |
| 131 | Martinez L, Multani JK, Sun K, Kalich BA, Miglins ML, Sidelnikov E, et al. Timing of PCSK9i initiation and its relationship with clinical outcomes in US patients with prior cardiovascular events: A claims database analysis. Journal of Clinical Lipidology. 2023;17(4 Supplement):e44-e5. | 3: Wrong outcome |
| 132 | Martinez-Poles J, Nedkova-Hristova V, Escribano-Paredes JB, Garcia-Madrona S, Sanchez Sanchez A, Vera R, et al. Evolocumab in secondary prevention of ischemic stroke: Real-life experience. European Stroke Journal. 2019;4(Supplement 1):558. | 3: Wrong outcome |
| 133 | Masana L, Lopez Miranda J, Civeira F, Reinares L, Guijarro C, Plana N, et al. Clinical profile of patients treated with evolocumab in lipid/internal medicine units of Spain. Observational study (RETOSS-IMU). Clinica e Investigacion en Arteriosclerosis. 2020;32(5):183-92. | 3: Wrong outcome |
| 134 | Masana L, Lopez-Miranda J, Civeira F, Reinares L, Maraver JM, Plana N, et al. Evolocumab is mainly prescribed in fh patients with/without atherosclerotic cardiovascular disease (ASCVD) in lipid/internal medicine units in Spain: A retrospective, observational study (RETOSS-IMU). Atherosclerosis. 2019;287((Masana, Plana) Hospital Universitari Sant Joan, Internal Medicine, Reus, Spain(Lopez-Miranda) Hospital Universitario Reina Sofia, Internal Medicine, Cordoba, Spain(Civeira) Hospital Universitario Miguel Servet, Internal Medicine, Zaragoza, Spain(Reinares):e198. | 3: Wrong outcome |
| 135 | Matta A, Bongard V, Bouisset F, Taraszkiewicz D, Rabes JP, Ferrieres J. Real-world efficacy of proprotein convertase subtilisin/kexin type 9 inhibitors (PCSK9i) in heterozygous familial hypercholesterolemia patients referred for lipoprotein apheresis. Medical Science Monitor. 2021;27((Matta, Bongard, Bouisset, Taraszkiewicz, Ferrieres) Department of Cardiology, UMR, INSERM 1295, Toulouse-Rangueil University Hospital, Toulouse University, School of Medicine, Toulouse, France(Matta, Bongard, Bouisset, Taraszkiewicz, Ferrieres) Departmen):e928784. | 3: Wrong outcome |
| 136 | Mazhar F, Hjemdahl P, Clase CM, Johnell K, Jernberg T, Carrero JJ. Lipid-lowering treatment intensity, persistence, adherence and goal attainment in patients with coronary heart disease. American Heart Journal. 2022;251((Mazhar, Johnell) Department of Medical Epidemiology and Biostatistics, Karolinska Institute, Stockholm, Sweden(Hjemdahl) Department of Medicine Solna, Clinical Epidemiology Unit, Karolinska Institute and Clinical Pharmacology, Karolinska University Hospi):78-90. | 1: Wrong intervention |
| 137 | McCollum JC, Clary JM, Guglin ME, Rao RA. Utilization of PCSK-9 inhibitors in statin intolerant cardiac transplant patients. Journal of Cardiac Failure. 2020;26(10 Supplement):S106-S7. | 3: Wrong outcome |
| 138 | Medvedeva EA, Grigorenko EA, Mitkovskaya NP. Innovative lipid-lowering therapy: experience of inclisiran use in the Republic of Belarus. Russian Journal of Cardiology. 2023;28(4):53-61. | 5: Wrong language |
| 139 | Michael Kuehl M, Jozwiak J, Binner C, Fischer J, Hahn J, Meyer A, et al. Treatment of hypercholesterolaemia with PCSK-9 inhibitors in patients after cardiac transplantation. European Journal of Heart Failure. 2018;20(Supplement 1):78. | 3: Wrong outcome |
| 140 | Mondelo Garcia C, Garcia Queiruga M, Balea Filgueiras J, Fernandez Bargiela N, Gimenez Arufe V, Fernandez Oliveira C, et al. Real-world effectiveness and safety of alirocumab. International Journal of Clinical Pharmacy. 2019;41(1):377-8. | 3: Wrong outcome |
| 141 | Monforte Gasque MP, Varela Martinez I, Moncin Torres CA. Clinical experience of new lipid-lowering therapies: Evolocumab and alirocumab. European Journal of Hospital Pharmacy. 2019;26(Supplement 1):A81. | 3: Wrong outcome |
| 142 | Monge I, Acin P, Navarrete-Rouco E, Recasens L, Pedro-Botet J, Oliveras A, et al. Efficacy, safety and acceptance of treatment with alirocumab or evolocumab in patients with dyslipidaemia. European Journal of Hospital Pharmacy. 2019;26(Supplement 1):A78-A9. | 3: Wrong outcome |
| 143 | Mongiello P, Petti R, Mitaritonna A, Ciaccia A, Notarangelo ML, Lombardi R. Adherence to alirocumab or evolocumab therapy in real life. Giornale Italiano di Farmacia Clinica. 2023;37(3):104-12. | 5: Wrong language |
| 144 | Muccioli S, Giglio C, Annibali G, Cerutti E, Civera S, Casati R, et al. The importance of intensive lipid-lowering therapy after acute coronary syndrome: Changing the paradigm to improve the achievement of targets. Giornale Italiano di Cardiologia. 2022;23(7):553-61. | 5: Wrong language |
| 145 | Mues K, Bratton E, Wirtz HS, Maya JF, Muntner PM, Wang TY. Patient characteristics and lipid lowering treatment of early adopters of PCSK9i therapies in the United States. Circulation. 2017;136(Supplement 1). | 4: Wrong publication type |
| 146 | Mugwagwa A. Reductions in LDL-C within the first year of treatment with inclisiran: Results from a multicentre real-world cohort. Heart Lung and Circulation. 2024;33(Supplement 4):S532-S3. | 3: Wrong outcome |
| 147 | Mulverstedt S, Klausen IC, Kanstrup H, Knold J, Andersen LJ, Theilade J, et al. Treatment of hypercholesterolaemia with PCSK9 inhibitors in Denmark. Assessment of real-life data; extent and efficacy after the first years of clinical use. European Heart Journal. 2019;40(Supplement 1):1830. | 3: Wrong outcome |
| 148 | Mulverstedt S, Klausen IC, Martinsen MH, Kanstrup H, Thomsen KK, Knold J, et al. Treatment of hypercholesterolaemia with PCSK-9 inhibitors in Denmark. Assessment of real-life data; safety an extent of adverse effects after the first years of clinical use. European Heart Journal. 2019;40(Supplement 1):313. | 3: Wrong outcome |
| 149 | Nanna MG, Nelson AJ, Haynes K, Shambhu S, Eapen Z, Cziraky MJ, et al. Lipid-lowering treatment among older patients with atherosclerotic cardiovascular disease. Journal of the American Geriatrics Society. 2023;71(4):1243-9. | 3: Wrong outcome |
| 150 | Napolitano B, Napolitano V, Napolitano L, Maretta A, Pulicano M, Spagnuolo V. Safety of evolocumab use in clinical practice in subjects with severe hyperlipemia and statins intolerance. Atherosclerosis. 2021;331((Napolitano, Napolitano, Napolitano) Internship student, Internal Medicine, Cosenza, Italy(Maretta) UNICZ University of Catanzaro, Department Of Pharmacy, Catanzaro, Italy(Pulicano) Nurse, Self-employed Professional, Cosenza, Italy(Spagnuolo) AO of Cosenz):e164. | 3: Wrong outcome |
| 151 | Narayanan D, Holloway YL, Mohammed K. An audit on the use of PCSK9 monoclonal inhibitor therapy in lipid clinic at a tertiary referral centre. Atherosclerosis Supplements. 2017;28((Narayanan) Chemical Pathology and Metabolic Medicine, Hull and East Yorkshire Hospitals NHS Trust, United Kingdom(Holloway) Cardiology, Hull and East Yorkshire Hospitals NHS Trust, United Kingdom(Mohammed) Diabetes and Endocrinology, Hull and East Yorksh):e16-e7. | 3: Wrong outcome |
| 152 | Nasso G, Larosa C, Bartolomucci F, Brigiani MS, Contegiacomo G, Demola MA, et al. Safety and efficacy of PCSK9 inhibitors in patients with acute coronary syndrome who underwent coronary artery bypass grafts: A comparative retrospective analysis. Journal of Clinical Medicine. 2024;13(3):907. | 3: Wrong outcome |
| 153 | Nasso G, Vignaroli W, Amodeo V, Bartolomucci F, Larosa C, Contegiacomo G, et al. Evolocumab treatment in dyslipidemic patients undergoing coronary artery bypass grafting: One-year safety and efficacy results. Journal of Clinical Medicine. 2024;13(10):2987. | 3: Wrong outcome |
| 154 | Nikitin AE, Averin EE, Rozhkov DE, Sozykin AV, Procenko GA. Alirocumab administration experience to achieve low density lipoprotein cholesterol target levels in secondary prevention of cardiovascular disease. Rational Pharmacotherapy in Cardiology. 2020;16(1):33-9. | 5: Wrong language |
| 155 | Niu X, Parlapalli A, Ma X, Park J, McElligott S, Osei-Wusu A. Characteristics of patients initiating inclisiran in the real world: A retrospective analysis of a US database. Journal of Clinical Lipidology. 2023;17(4 Supplement):e57-e8. | 3: Wrong outcome |
| 156 | Padam P, Barton L, Wilson S, David A, Walji S, De Lorenzo F, et al. Reduction in LDL-C following treatment with inclisiran at 2 months in a single centre lipid clinic cohort. Atherosclerosis. 2023;379(Supplement 1):S171. | 3: Wrong outcome |
| 157 | Padam P, Barton L, Wilson S, David A, Walji S, De Lorenzo F, et al. Lipid lowering with inclisiran: A real-world single-centre experience. Open Heart. 2022;9(2):e002184. | 3: Wrong outcome |
| 158 | Palomo Palomo C, Romero Alonso MM, Guerra Estevez D, Estaire Gutierrez J, Parrado Gonzalez A, Reyes Malia M. Use and effectiveness of treatment with alirocumab and evolocumab in real clinical practice. International Journal of Clinical Pharmacy. 2022;44(6):1566. | 3: Wrong outcome |
| 159 | Pandav J, Saranu R, Muhsin S, Murakami N, Gabardi S, Safa K, et al. Efficacy and safety of PCSK-9 inhibitor, evolocumab for lipid management in kidney transplant recipients. American Journal of Transplantation. 2022;22(Supplement 3):678. | 4: Wrong publication type |
| 160 | Perrone V, Iacolare B, Dovizio M, Andretta M, Bacca M, Barbieri A, et al. PCSK-9 inhibitors for the control of hypercholesterolemia: Eligibility for treatment, prescription appropriateness and outcomes, in a real-world clinical setting. Value in Health. 2022;25(12 Supplement):S275-S6. | 3: Wrong outcome |
| 161 | Petkova R, Postadzhiyan A, Petrov I, Tisheva S, Borizanova-Petkova A, Bridges I. Baseline characteristics of Bulgarian patients prescribed evolocumab in clinical practice. Atherosclerosis. 2020;315((Petkova) Amgen, General Medicine, Sofia, Bulgaria(Postadzhiyan) UMBAL Sveta Anna, Cardiology, Sofia, Bulgaria(Petrov) City Clinic, City Clinic Heart And Vascular Institute, Sofia, Bulgaria(Tisheva) Medical University of Pleven, Head Of Departement Of Car):e212. | 3: Wrong outcome |
| 162 | Philippart M, Guilmot A, Tarantino E, Sablon G, Descamps O. Real life experience with anti-PSK9 in familial hypercholesterolemia in Belgium. Acta Clinica Belgica. 2020;74(Supplement 1):34. | 3: Wrong outcome |
| 163 | Picciolo A, Simonelli F, Corliano L, Tommasi R, Mussardo M, Tondo A, et al. 2010-2020 coronary heart disease secondary prevention: PCSK9-inhibitors role. European Heart Journal, Supplement. 2021;23(SUPPL C):C101. | 3: Wrong outcome |
| 164 | Pisciotta L, Catapano AL, Maggioni AP, Rossi F, Tirone G, Notarianni L, et al. Observational multicenter study on effectiveness and tolerability of alirocumab in real world, the Omero study: Interim data from the fist 352 participants. Atherosclerosis. 2021;331((Pisciotta) University of Genoa, Unige Department Of Internal Medicine And Medical Specialties, Genoa, Italy(Catapano) Epidemiology and Preventive Pharmacology Service (SEFAP), Department Of Pharmacological And Biomolecular Sciences, University Of Milan):e166. | 3: Wrong outcome |
| 165 | Poudel M, Stoyanova D, Van Buuren F, Horstkotte D, Mellwig KP. Reduction of LDL and Lp (a) under the PCSK9 inhibitor Evolocumab/Alirozumab in patients with familial hypercholesterolemia or manifest atherosclerosis. European Journal of Preventive Cardiology. 2017;24(1 Supplement 1):S85. | 3: Wrong outcome |
| 166 | Ray KK, Molemans B, Marieke Schoonen W, Giovas P, Bray S, Kiru G, et al. EU-wide cross-sectional observational study of lipid-modifying therapy use in secondary and primary care: The DAVINCI study. European Journal of Preventive Cardiology. 2021;28(11):1279-89. | 3: Wrong outcome |
| 167 | Ray KK, Troquay RPT, Visseren FLJ, Leiter LA, Scott Wright R, Vikarunnessa S, et al. Long-term efficacy and safety of inclisiran in patients with high cardiovascular risk and elevated LDL cholesterol (ORION-3): Results from the 4-year open-label extension of the ORION-1 trial. The Lancet Diabetes and Endocrinology. 2023;11(2):109-19. | 2: Wrong study design |
| 168 | Razek O, Cermakova L, Armani H, Lee T, Francis GA, Mancini GBJ, et al. Attainment of recommended lipid targets in patients with familial hypercholesterolemia: Real-world experience with PCSK9 inhibitors. Canadian Journal of Cardiology. 2018;34(8):1004-9. | 3: Wrong outcome |
| 169 | Reigota CP, Fernandes DDA, Vilao F, De Moura JP. Use of PCSK9 inhibitors in a tertiary hospital in Portugal - a persistent conundrum. Atherosclerosis. 2020;315((Reigota) Coimbra Hospital and University Centre (CHUC), 1internal Medicine, Coimbra, Portugal(Fernandes) Coimbra Hospital and University Centre, Cardiology, Coimbra, Portugal(Vilao, De Moura) Coimbra Hospital and University Centre, Internal Medicine, Coi):e281. | 3: Wrong outcome |
| 170 | Rodriguez Escobedo R, Gonzalez Martinez S, Diaz Naya L, Suarez Gutierrez L, Fernandez Morera JL, Riestra Fernandez M, et al. Real-life efficacy and safety of PCSK9 inhibitors treatment: Experience in three hospitals in Asturias. Semergen. 2021;47(6):369-75. | 5: Wrong language |
| 171 | Romagnoli A, Tommaso RD, Guarino FR, Colanardi P, De Vita F, D'Ovidio P. Drug utilization patterns of PCSK9 inhibitors: adherence, persistence and appropriateness prescription in a real life analysis. Giornale Italiano di Farmacia Clinica. 2023;37(2):38-45. | 5: Wrong language |
| 172 | Royuela M, Alonso MP, Balet A, El Boutrouki O, Bonet M, Ruiz D. Effectivity, safety and adherence with PCKS9 inhibitors. Atherosclerosis. 2022;355((Royuela) Internal Medicine. Lipid Unit, ALTHAIA. Xarxa assistencial Universitaria de Manresa, Manresa, Spain(Alonso, Balet) Pharmacy, ALTHAIA. Xarxa assistencial universitaria de Manresa, Manresa, Spain(El Boutrouki, Bonet, Ruiz) Internal Medicine, ALTHA):224. | 4: Wrong publication type |
| 173 | Saez Rodriguez MI, Arenas Villafranca JJ, Montero Salgado B, Chinchurreta Capote PA, Tortajada Goitia B. Real-world experience with PCSK9 inhibitors protocol for hypercholesterolaemia. European Journal of Hospital Pharmacy. 2022;29(SUPPL 1):A69-A70. | 3: Wrong outcome |
| 174 | Salim HY, Lwin K, Khoo C, Wilson D. Management of hyperlipidaemia following acute coronary syndrome: A retrospective audit. The British journal of cardiology. 2021;28(2):17. | 3: Wrong outcome |
| 175 | Santos RD, Stein EA, Hovingh GK, Blom DJ, Soran H, Watts GF, et al. Long-term evolocumab in patients with familial hypercholesterolemia. Journal of the American College of Cardiology. 2020;75(6):565-74. | 2: Wrong study design |
| 176 | Sarsam S, Berry A, Degheim G, Singh R, Zughaib M. Real-world use of PCSK9 inhibitors: A single-center experience. Journal of International Medical Research. 2019;47(1):265-70. | 3: Wrong outcome |
| 177 | Sbrana F, Dal Pino B, Bigazzi F, Ripoli A, Corciulo C, Lo Surdo G, et al. Major cardiovascular events increase in long-term proprotein convertase subtilisin/kexin type 9 inhibitors therapy: The Tuscany cost-effective study. Journal of Cardiovascular Medicine. 2023;24(11):808-14. | 3: Wrong outcome |
| 178 | Schlam I, Huda A, Min SH, Mahida C, Shah P, Wang P, et al. 10-Year cardiovascular risk reduction, efficacy and safety of alirocumab and evolocumab, in addition to maximal tolerated cholesterol lowering therapy: Post-commercialization study. Journal of the American College of Cardiology. 2017;69(11 Supplement 1):1717. | 3: Wrong outcome |
| 179 | Schwartz GG, Szarek M, Bhatt DL, Bittner VA, Bujas-Bobanovic M, Diaz R, et al. Transiently achieved very low LDL-cholesterol levels by statin and alirocumab after acute coronary syndrome are associated with cardiovascular risk reduction: The ODYSSEY OUTCOMES trial. European heart journal. 2023(em8, 8006263). | 2: Wrong study design |
| 180 | Scicchitano P, Locuratolo N, Lillo A, Sublimi Saponetti L, Palumbo V, Lanzone S, et al. The follow-up of patients after acute coronary syndrome: The Apulian PONTE-ACS project. European Heart Journal, Supplement. 2022;24(SUPPL C):C134-C5. | 3: Wrong outcome |
| 181 | Shah P, Glueck CJ, Goldenberg N, Min S, Mahida C, Schlam I, et al. Efficacy, safety, low density lipoprotein cholesterol lowering, and calculated 10-year cardiovascular risk reduction of alirocumab and evolocumab in addition to maximal tolerated cholesterol lowering therapy: A post-commercialization study. Lipids in Health and Disease. 2017;16(1):1-12. | 3: Wrong outcome |
| 182 | Shaik A, Cannon C, Liu Y, Mues K, Alam S, de Lemos J, et al. Use of lipid-lowering therapies in patients with CKD and ASCVD: A 1-year update from GOULD. Journal of Clinical Lipidology. 2021;15(5):e1. | 3: Wrong outcome |
| 183 | Shaik A, Cannon C, Liu Y, Mues K, Alam S, Lemos JD, et al. Use of lipid-lowering therapies in patients with CKD and ASCVD: A 1-year update from GOULD*. Journal of Clinical Lipidology. 2020;14(4):603-4. | 3: Wrong outcome |
| 184 | Smith A, Johnson D, Banks J, Keith SW, Karalis DG. Trends in PCSK9 inhibitor prescriptions before and after the price reduction in patients with atherosclerotic cardiovascular disease. Journal of Clinical Medicine. 2021;10(17):3828. | 3: Wrong outcome |
| 185 | Smith A, Johnson D, Karalis D. Changes in prescribing patterns before and after price reduction in PCSK9 inhibitors. Circulation: Cardiovascular Quality and Outcomes. 2020;13(SUPPL 1). | 3: Wrong outcome |
| 186 | Steffens D, Bramlage P, Muller J, Dorn C, Paar WD, Scheeff C, et al. Intensified lipid-lowering treatment with alirocumab in patients with coronary heart disease. Open Heart. 2021;8(1):e001572. | 3: Wrong outcome |
| 187 | Stronati G, Parente E, Nazziconi M, Brugiatelli L, Bastianoni G, Rago A, et al. PCSK9 inhibitors and incidence of arrhythmias in clinical practice: A phase IV multicentric study. Europace. 2023;25(Supplement 1):i852 | 3: Wrong outcome |
| 188 | Stryker M, Kane M, Busch R. An evaluation of proprotein convertase subtilisin/kexin type 9 (PCSK9) inhibitors in patients with diabetes mellitus. Journal of Clinical Lipidology. 2016;10(3):720. | 3: Wrong outcome |
| 189 | Terentes-Printzios D, Dima I, Benardos P, Mitrou P, Mathioudakis K, Tsolakidis A, et al. Real-world data on treatment patterns in at least high cardiovascular risk patients on dual and triple lipid lowering therapy in a Hellenic nationwide e-prescription database. International Journal of Cardiology: Cardiovascular Risk and Prevention. 2024;21((Terentes-Printzios, Dima, Tsioufis, Vlachopoulos) First Department of Cardiology, Hippokration Hospital, Medical School, National and Kapodistrian University of Athens, Greece(Benardos) National Technical University of Athens, School of Mechanical Engine):200261. | 1: Wrong intervention |
| 190 | Thum CH, Rosman A, Choong SY. LDL-C lowering with inclisiran - a single centre experience. International Journal of Cardiology. 2023;393(Supplement):131511. | 3: Wrong outcome |
| 191 | Trippi J, Jones B. Changing between PCSK9 inhibitor medications. Journal of Clinical Lipidology. 2019;13(3):e8. | 3: Wrong outcome |
| 192 | Tselmin S, Julius U, Weinert N, Bornstein SR, Schatz U. Experience with proprotein convertase subtilisin/kexine type 9 inhibitors (PCSK9i) in patients undergoing lipoprotein apheresis. Atherosclerosis Supplements. 2019;40(dyj, 100973461):38-43. | 3: Wrong outcome |
| 193 | Verdickt S, Van der Schueren B, Vangoitsenhoven R, Descamps OS, Mertens A. Belgian data of ODYSSEY APPRISE: Stringent LDL-c targets are in reach when using all available tools. International Journal of Clinical Practice. 2021;75(12):e14916. | 2: Wrong study design |
| 194 | Villacanas Palomares MV, Colon Lopez De Dicastillo A, Gutierrez Perez I, Uriarte Estefania F, Santos Del Prado R, Lorenzo Martin S, et al. Effectiveness and safety of evolocumab in real clinical practice. European Journal of Hospital Pharmacy. 2019;26(Supplement 1):A214-A5. | 2: Wrong study design |
| 195 | Vitale GG, Nugara C, Sarullo S, Vitale S, Sarullo FM. PCKS9 inhibitors in cardiac rehabilitation a single center experience. European Journal of Preventive Cardiology. 2020;27(1 SUPPL4):S61. | 3: Wrong outcome |
| 196 | Wang W. Real-world use of inclisiran in outpatient physician clinics. Heart Lung and Circulation. 2024;33(Supplement 4):S532. | 3: Wrong outcome |
| 197 | Warden BA, Miles JR, Oleaga C, Ganda OP, Duell PB, Purnell JQ, et al. Unusual responses to PCSK9 inhibitors in a clinical cohort utilizing a structured follow-up protocol. American journal of preventive cardiology. 2020;1(101769122):100012. | 3: Wrong outcome |
| 198 | Wassmuth S, Rohe K, Noack F, Noutsias M, Treede H, Schlitt A. Adherence to lipid-lowering therapy in patients with coronary heart disease from the state of Saxony-Anhalt, Germany. Vascular Health and Risk Management. 2019;15((Wasmuth, Rohe, Noutsias) Department of Medicine III, University Clinic, Halle, Germany(Noack) Emergency Department and Department of Medicine II, University Clinic, Halle, Germany(Treede) Department of Cardiac Surgery, University Clinic, Halle, Germany(S):477-83. | 1: Wrong intervention |
| 199 | Wierzbicki AS, Reynolds T, Vijayenthiran H, McMahon Z, Ramachandran R, Viljoen A, et al. Determinants of response and tolerability of PCSK9 inhibitor therapy. Journal of Clinical Lipidology. 2019;13(3):e57-e8. | 4: Wrong publication type |
| 200 | Wilkinson M, Chiou T, Taub P, Mazdeyasnan D. Real-world experience with inclisiran at a large academic lipid clinic. Journal of Clinical Lipidology. 2024;18(4 Supplement):e553-e4. | 3: Wrong outcome |
| 201 | Xu X, Chai M, Cheng Y, Peng P, Liu X, Yan Z, et al. Efficacy and safety of evolocumab in reducing low-density lipoprotein cholesterol levels in chinese patients with non-st-segment elevation acute coronary syndrome. Current Vascular Pharmacology. 2021;19(4):429-37. | 3: Wrong outcome |
| 202 | Yalamanchi R, Vinayagamoorthy N, Showkathali R. Efficacy and safety of evolocumab in post-acute coronary syndrome patients - a real world experience. Indian Heart Journal. 2023;75(Supplement 1):S65. | 3: Wrong outcome |
| 203 | Yanez Feria D, Selvi Sabater P, Pelaez Bejarano A, Montero Perez O, Santos Rubio MD. Analysis of adaptation to a protocol of use of the PCSK9 inhibitors. European Journal of Hospital Pharmacy. 2019;26(Supplement 1):A81. | 4: Wrong publication type |
| 204 | Zhang A, Wu Y, Zhang Y, Hu W, Chen P, Chen K, et al. Early addition of evolocumab to statin treatment in patients with acute coronary syndrome and multivessel disease undergoing percutaneous coronary intervention. Reviews in Cardiovascular Medicine. 2023;24(9):rcm2409270. | 2: Wrong study design |
| 205 | Zhang T, Zhang Y, Yang Y, Liao H, Li X, Liu R, et al. Real-world effectiveness and safety of evolocumab in very high-risk atherosclerotic cardiovascular disease patients with acute ischemic stroke. medRxiv. 2023:2023.06.25.23291877. | 3: Wrong outcome |
| 206 | Zhang Y, Zhang B, Chen Z, Wei Y, Chen P, Chang C, et al. Early initiation of evolocumab treatment in Chinese patients with acute coronary syndrome undergoing percutaneous coronary intervention. Clinical Therapeutics. 2022;44(6):901-12. | 2: Wrong study design |

# List of included studies

**Table S4A.** Number of studies on monoclonal antibodies and siRNA PCSK9 inhibitors included in systematic review by publication type, medication adherence phase, country, and data source

|  | **Monoclonal antibodies** | | | **siRNA** | **All three PCSK9 inhibitors** |
| --- | --- | --- | --- | --- | --- |
|  | **Both** | **Alirocumab only** | **Evolocumab only** | **Inclisiran** |  |
| **Publication type** | | | | | |
| All publications | 67 | 4 | 14 | 7 | 2 |
| Journal articles | 43 | 3 | 11 | 3 | 0 |
| Conference abstracts or brief reports | 24 | 1 | 3 | 4 | 2 |
| **Medication adherence phase studied** | | | | | |
| **Initiation** | 18 | 0 | 2 | 2 | 0 |
| **Implementation (any measure)** | 29 | 2 | 6 | 5 | 2 |
| MPR | 3 | 2 | 0 | 0 | 0 |
| PDC | 11 | 1 | 1 | 0 | 1 |
| Adherence ≥ 80% | 14 | 0 | 3 | 0 | 1 |
| Proportion of patients received doses* | 0 | 0 | 0 | 3 | 1 |
| Adherence (%)* | 11 | 0 | 4 | 1 | 0 |
| Other definitions* | 12 | 0 | 2 | 2 | 0 |
| **Persistence (any measure)** | 55 | 3 | 11 | 4 | 1 |
| Proportion (%) | 22 | 1 | 4 | 0 | 0 |
| Time from PCSK9i initiation to permanent discontinuation | 5 | 0 | 2 | 0 | 1 |
| Kaplan-Meier estimator (%)* | 2 | 0 | 1 | 0 | 0 |
| Other definitions* | 3 | 0 | 1 | 0 | 0 |
| Discontinuation rate | 53 | 3 | 10 | 4 | 1 |
| **Reinitiation (any measure)** | 10 | 1 | 0 | 0 | 0 |
| Resumption rate | 7 | 0 | 0 | 0 | 0 |
| Switching rate | 9 | 1 | 0 | 0 | 0 |
| **Region** | | | | | |
| US | 27 | 0 | 0 | 2 | 2 |
| Europe | 35 | 3 | 7 | 2 | 0 |
| Others | 5 | 1 | 7 | 3 | 0 |
| **Data source** | | | | | |
| Outpatient specialty clinics | 23 | 2 | 3 | 5 | 0 |
| Database | 37 | 2 | 6 | 2 | 2 |
| Patient support programs | 1 | 0 | 1 | 0 | 0 |
| Mixed data sources | 2 | 0 | 3 | 0 | 0 |
| Survey | 1 | 0 | 0 | 0 | 0 |
| Not reported | 3 | 0 | 1 | 0 | 0 |

*Measures of medication adherence that were not pooled due to variations in definitions between studies or a limited number of published studies

**Table S4B.** General characteristics (study overview, setting and outcome definitions used) of all studies included in the systematic review of PCSK9 inhibitor medication adherence

| **#** | **Study (Lead author, year)** | **Sample size** | **Country** | **Study design** | **Data source** | **Study enrolment period** | **Eligibility criteria** | **PCSK9 inhibitor*** | **Medication adherence phase studied** | **Outcome definition** |  |
| --- | --- | --- | --- | --- | --- | --- | --- | --- | --- | --- | --- |
| **Journal Articles** | | | | | | | | | | | |
| 1 | Alonso 2023 | 696 | Spain | Cohort study | Outpatient specialty clinics | 02/01/2016 to 30/09/2021 | A cohort of subjects with a molecular diagnosis of FH and their affected and non-affected relatives which was initiated in 2004 | moAbs (A&E) | - Persistence - Reinitiation | Non-persistent: gap ≥ 60 days |  |
| 2 | Arca 2023a | 1263 | Italy | Cohort study | Database | 02/01/2017 to 31/12/2019 | Patients with HeFH and HoFH treated with alirocumab or evolocumab in Italy, who started treatment between February 2017 and December 2019 | moAbs (A&E) | - Implementation - Persistence | Adherence reported as %  Adherent: Adherence ≥ 80%  Non-persistent: discontinued the therapy before the expected observation |  |
|  | Arca 2023b | 36 |  |  |  |  |  |  |  |  |  |
| 3 | Barrios 2020 | 186 | Spain | Cohort study | Database | 02/01/2016 to 15/05/2017 | Adults prescribed evolocumab between February 1, 2016 and May 15, 2017, had received at least one dose of evolocumab, had at least one LDL-C measurement in the 12 weeks prior to starting evolocumab, with informed consent | moAbs (E) | - Implementation - Persistence | Adherent: Adherence ≥ 80%  Non-persistent: gap not specified |  |
| 4 | Bosch 2024 | 193 | Spain | Cohort study | Database | 01/01/2016 to 31/12/2021 | All adult patients with primary hypercholesterolemia or mixed dyslipidaemia who started treatment with evolocumab or alirocumab at VHUH between January 1, 2016 and December 31, 2021 | moAbs (A&E) | - Persistence | Non-persistent: gap not specified |  |
| 5 | Bradley 2019 | 1269 | United States | Cross-sectional study | Patient support programs | 03/07/2017 to 11/02/2017 | All patients who had participated in a company-sponsored patient support program for PCSK9 inhibitor therapy, with informed consent | moAbs (A&E) | - Initiation - Persistence | Initiation: No. starting / No. prescribed  Non-persistent: discontinued therapy by the time they completed the survey |  |
| 6 | Cannon 2021 | 554 | United States | Cohort study | Mixed | 12/01/2016 to 31/07/2018 | Adults who have established ASCVD, received some type of stable LLT for at least 4 weeks prior to enrolment, including those in the PCSK9i cohort, at the discretion of the treating physician | moAbs (A&E) | - Persistence | Non-persistent: not receiving a PCSK9i drug at 24 months post initiation |  |
| 7 | Caso 2022 | 130 | Italy | Cohort study | Database | 03/09/2020 to 17/05/2020 | Patients frequently undertaking PCSK9i therapy at the Cardiac Diagnostic Unit of the University of Campania “Luigi Vanvitelli”, and who missed the follow-up visit from 9 March 2020 and 17 May 2020, due to the COVID-19 pandemic lockdown restrictions | moAbs (E) | - Implementation | Adherence assessed via MPR  Adherent: threshold not specified |  |
| 8 | Chai 2023 | 63 | China | Cohort study | Database | 03/01/2019 to 31/01/2020 | Patients from the hospital’s Cardiology department who received at least one prescription of evolocumab (140 mg) between March 2019 and January 2020, with very high-risk status and failure to achieve a goal LDL-C level of <1.4 mmol/L despite statin therapy for at least 4 weeks | moAbs (E) | - Persistence | Non-persistent: gap ≥ 30 days |  |
| 9 | Chlebus 2022 | 55 | Poland | Cohort study | Database | 03/01/2019 to 31/01/2021 | Patients with FH and LDL-C levels > 160 mg/dL (> 4.1 mmol/L) | moAbs (A&E) | - Persistence | Non-persistent: gap not specified |  |
| 10 | Chng 2022 | 80 | Singapore | Cohort study | Outpatient specialty clinics | 06/01/2017 to 31/07/2021 | Age ≥21 years, administration of at least one dose of PCSK9 inhibitor, and minimum follow-up of 1 month, and written informed consent | moAbs (A&E) | - Implementation - Persistence - Reinitiation | Adherence assessed via MPR  Adherent: MPR ≥ 80%  Non-persistent: gap not specified |  |
| 11 | Davidson 2020 | 55 | United States | Cohort study | Outpatient specialty clinics | 08/01/2015 to 31/01/2017 | All adult patients initially prescribed either alirocumab 75 mg every 2 weeks or evolocumab 140 mg every 2 weeks at the Lipid Clinic between August 2015 and January 2017 with a history of self-reported statin intolerance | moAbs (A&E) | - Initiation - Persistence | Initiation: No. starting / No. prescribed  Non-persistent: gap not specified |  |
| 12 | Davis 2020 | 61 | United States | Cohort study | Outpatient specialty clinics | 09/01/2015 to 31/03/2019 | Patients age ≥ 65 years, referred to the pharmacist clinic and received a prescription for a PCSK9i from September 2015 to March 2019 | moAbs (A&E) | - Initiation - Persistence | Initiation: No. starting / No. eligible  Non-persistent: gap not specified |  |
| 13 | Donald 2022 | 477 | United States | Cohort study | Outpatient specialty clinics | 09/01/2015 to 31/08/2018 | Patients who were prescribed and initiated on a PCSK9i by a VUMC provider between September 1, 2015, and August 31, 2018 | moAbs (A&E) | - Persistence | Non-persistent: gap ≥ 60 days |  |
| 14 | Elis 2023 | 623 | Israel | Cohort study | Database | 08/01/2016 to 05/01/2020 | Adult MHS members who have initiated treatment with alirocumab from 1 August 2016 to 1 May 2020, having at least two dispenses of alirocumab within 90 days from the index date, a blood lipids laboratory test within 180 days before the index date, and at least one additional one up to 120 days after it | moAbs (A) | - Implementation | Adherence assessed via PDC and MPR  Adherent: PDC or MPR ≥ 80% |  |
| 15 | Eloso 2023a | 862 | United States | Cohort study | Database | 21/08/2015 to 30/09/2020 | Veterans aged ≥18 years who had at least one outpatient prescription for alirocumab and/or evolocumab filled within VHA between August 21, 2015, and September 30, 2020 | moAbs (A&E) | - Implementation - Persistence | Adherence assessed via MPR  Adherent: MPR ≥ 80%  Non-persistent: gap ≥ 60 days |  |
|  | Eloso 2023b | 526 |  |  |  |  |  |  |  |  |  |
|  | Eloso 2023c | 269 |  |  |  |  |  |  |  |  |  |
|  | Eloso 2023d | 594 |  |  |  |  |  |  |  |  |  |
| 16 | Engebretsen 2022 | 1266 | Norway | Cohort study | Database | 01/01/2010 to 31/12/2019 | Patients with prescription fills on statins, ezetimibe, and PCSK9 inhibitors in 2010-2019 from the Norwegian Prescription Database | moAbs (A&E) | - Implementation | Adherence assessed via PDC |  |
| 17 | Fairman 2017 | 390 | United States | Cohort study | Database | 08/01/2015 to 31/12/2015 | Aged ≥18 years and continuously enrolled for benefits throughout 2015, newly initiated pharmacotherapy with either a) a PCSK9 inhibitor, with or without one or more statins (PCSK-9 inhibitor cohort, n=390) or b) highest-intensity statin therapy without a PCSK-9 inhibitor (statin cohort, n=26,306) during the time period from August 1, 2015, to December 31, 2015. | moAbs (A&E) | - Persistence | Non-persistent: gap ≥ 60 days |  |
| 18 | Fischer 2021 | 237 | Austria | Cohort study | Database | 01/01/2016 to 31/09/2019 | Patients age >18 years, treatment with locally available PCSK9 inhibitors (alirocumab 75 or 150 mg, or evolocumab 140 mg) in routine care, available laboratory reports on LDL-C levels at first prescription and LDL-C during a follow-up period longer than 3 months | moAbs (A&E) | - Persistence - Reinitiation | Non-persistent: lost to follow-up (gap ≥ 365 days) or discontinued therapy |  |
| 19 | Galema-Boers 2023 | 436 | Netherlands | Cohort study | Outpatient specialty clinics | NA | Patients aged ≥18 years who started a PCSK9 inhibitor at the Erasmus MC university hospital outpatient lipid clinic in Rotterdam, the Netherlands, as part of clinical care | moAbs (A&E) | - Implementation - Persistence | Adherent: threshold not specified |  |
| 20 | Garcia-Pena 2023 | 906 | Columbia | Cohort study | Patient support programs | 01/01/2017 to 30/09/2021 | Patients entering the patient support program for evolocumab in Colombia from its inception in 2017-2021 | moAbs (E) | - Implementation - Persistence | Adherence assessed via MPR  Adherent: MPR ≥ 80%  Non-persistent: gap ≥ 60 days |  |
| 21 | Gargiulo 2024 | 771 | Italy | Cohort study | Database | 03/01/2020 to 31/05/2023 | Patients with ACS from the AT-TARGET-IT registry who received PCSK9i prescription during in-hospital stay or at hospital discharge for ACS or within 4 weeks from ACS hospitalization, according to national reimbursement rules | moAbs (A&E) | - Implementation - Persistence | Adherence assessed via MPR  Adherent: MPR ≥ 80%  Non-persistent: permanently stopped therapy during the observation period |  |
| 22 | Gayoso-Rey 2021 | 154 | Spain | Cohort study | Mixed | 01/01/2017 to 31/12/2019 | Patients with hypercholesterolemia who met the funding criteria that were established by the Central Autonomic Pharmacy and Therapeutics Commission (CAPTC) and began treatment with PCSK9is between January 2017 and December 2019, with a minimum treatment period of 3 months | moAbs (A&E) | - Implementation - Persistence | Adherence assessed via MPR  Non-persistent: gap not specified |  |
| 23 | Goicoechea 2022 | 60 | Spain | Cohort study | Mixed | 02/01/2016 to 31/08/2018 | Patients age ≥18 years who started treatment with evolocumab prescribed by a physician from a nephrology department between February 1, 2016 and August 31, 2018, who had received at least one dose of evolocumab, with at least one LDL-C measurement and one estimated glomerular filtration rate (eGFR) within 24 weeks prior to starting evolocumab | moAbs (E) | - Persistence | Non-persistent: gap not specified |  |
| 24 | Gupta 2023 | 578 | Canada, Mexico, Colombia, Saudi Arabia, Kuwait | Cohort study | Mixed | 08/01/2017 to 07/09/2019 | Patients 18 years of age, with informed consent who initiated on evolocumab during August 2017 to July 2019 at 49 sites across Canada, Mexico, Colombia, Saudi Arabia and Kuwait | moAbs (E) | - Persistence | Non-persistent: gap ≥ 56 days |  |
| 25 | Gurgoze 2018 | 164 | Netherlands | Cohort study | Database | 06/01/2015 to 30/11/2017 | Patients with hypercholesterolemia, mostly patients with FH not reaching target LDL-C levels despite maximally tolerated statin and ezetimibe therapy who were eligible for treatment with PCSK9 inhibitors were recruited from the outpatient clinic in a tertiary university hospital setting. All patients fulfilled the Dutch criteria for reimbursement of PCSK9 inhibitors | moAbs (A&E) | - Persistence | Non-persistent: gap not specified |  |
| 26 | Hines 2018 | 13151 | United States | Cohort study | Database | 01/01/2016 to 30/06/2016 | Commercially insured and Medicare patients initiating PCSK9i therapy with evolocumab or alirocumab between January 1, 2016 and June 30, 2016. | moAbs (A&E) | - Implementation - Persistence | Adherence assessed via PDC  Adherent: PDC ≥ 80%  Non-persistent: gap ≥ 60 days |  |
| 27 | Iqbal 2022 | 102 | United Arab Emirates | Cohort study | Outpatient specialty clinics | 01/01/2017 to 31/10/2020 | All patients aged between 18 and 85 years who initiated PCSK9 inhibitor treatment at ICLDC and who attended follow-up with repetition of the lipid panel on at least one subsequent occasion | moAbs (E) | - Implementation - Persistence | (Non-)adherent: threshold not specified  Non-persistent: gap ≥ 90 days |  |
| 28 | Iqbal 2024 | 146 | United Arab Emirates | Cohort study | Outpatient specialty clinics | 05/01/2021 to 31/12/2022 | All patients aged 18 years and older who initiated inclisiran treatment at ICLDC and who attended follow-up visits with repetition of the lipid panel on at least 1 subsequent occasion. Follow-up visits post initiation of inclisiran were planned at 3-month intervals. | siRNA (I) | - Implementation - Persistence | (Non-)adherent: threshold not specified  Non-persistent: gap ≥ 90 days |  |
| 29 | Kaufman 2019 | 271 | United States | Cohort study | Outpatient specialty clinics | 07/01/2015 to 31/08/2018 | Patients who meet the standard of care indications for PCSK9i treatment, subjects were enrolled in the previously reported PCSK9i clinic of our Center for Preventive Cardiology at Oregon Health & Science University | moAbs (A&E) | - Persistence | Non-persistent: gap not specified |  |
| 30 | Khatib 2022 | 48 | United Kingdom | Cohort study | Outpatient specialty clinics | 02/01/2017 to 31/07/2018 | The first 100 individuals referred to the service | moAbs (A&E) | - Initiation - Persistence | Initiation: No. starting / No. eligible  Non-persistent: gap not specified |  |
| 31 | Kim 2023 | 91 | United States | Cohort study | Database | 04/01/2016 to 31/12/2020 | All adults (aged ≥18 years) at the 3 study sites who were dispensed a PCSK9i between April 1, 2016, and December 31, 2020 | moAbs (A&E) | - Persistence - Reinitiation | Non-persistent: gap not specified |  |
| 32 | Koenig 2024 | 1940 | Germany | Cohort study | Database | 07/01/2017 to 31/12/2017 | Patients with dyslipidemia who were newly prescribed LLTs between July and December 2017, and who were followed up for 39 months until March 2021 | moAbs (A&E) | - Implementation - Persistence | Adherence assessed via PDC  Non-persistent: gap ≥ 90 days |  |
| 33 | Kohli 2017 | 80 | United Kingdom | Cohort study | Outpatient specialty clinics | 07/01/2016 to 31/12/2016 | Prescription of PCSK-9 inhibitor therapy was initiated using National Institute of Health and Clinical Excellence (NICE) criteria for patients on maximally tolerated lipid-lowering therapy: patients with FH and LDL-C > 5 mmol/L; patients with established coronary heart disease (CHD) and LDL-C > 4 mmol/L; and patients with multi-vascular disease and LDL-C > 3.5 mmol/L | moAbs (A&E) | - Initiation - Persistence | Initiation: No. starting / No. eligible  Non-persistent: gap not specified |  |
| 34 | Lafratte 2023 | 178 | United States | Cohort study | Database | 04/01/2017 to 30/09/2019 | Patients aged ≥18 years old, continuously enrolled with medical and pharmacy benefits during the study period, and have no concurrent prescription claims for ezetimibe, who were newly initiated on a PCSK9i, either alirocumab or evolocumab, were identified using prescription claims (using National Drug Code from April 1, 2017, to September 30, 2019). Patients were included in the PCSK9i (pre-post) group if there were at least 2 fills of a new PCSK9i in the post period and at least 2 fills of a statin medication in the pre period | moAbs (A&E) | - Implementation - Persistence | Adherence assessed via PDC  Non-persistent: gap ≥ 60 days |  |
| 35 | Lahoz 2024 | 667 | United States | Cohort study | Database | 07/01/2015 to 31/12/2018 | Patients who had at least one ASCVD encounter, prescribed with at least one LLT (i.e. statins, ezetimibe, fibrates, and PCSK9i), or with a test result of LDL-C ≥70 mg/dL (any time prior to or 3 months after the index date) were included. Patients were required to have continuous enrolment (no allowance of gaps) 6 months prior to the index date (pre-index) and also a 12-month continuous post-index period | moAbs (A&E) | - Implementation - Persistence | Adherence assessed via PDC  Adherent: PDC ≥ 80%  Non-persistent: gap ≥ 60 days |  |
| 36 | Leitner 2020 | 112 | Austria | Cohort study | Outpatient specialty clinics | 09/01/2016 to 31/07/2018 | Adult patients (≥18 years) on treatment with alirocumab for hypercholesterolemia according to the summary of product characteristics (SmPC) and without contraindications, who did not participate in the ODYSSEY APPRISE study | moAbs (A) | - Persistence - Reinitiation | Non-persistent: gap not specified |  |
| 37 | Maciejko 2019 | 73 | United States | Cohort study | Outpatient specialty clinics | 08/01/2016 to 04/01/2018 | Hyperlipidemic patients referred by their physician to a hospital-based lipid clinic over a 20-month period (between August 1, 2016 and April 1, 2018) specifically for initiation of a PCSK9 inhibitor, who met existing FDA indications for this therapy | moAbs (A&E) | - Initiation - Persistence | Initiation: No. starting / No. eligible  Non-persistent: gap not specified |  |
| 38 | Mongiello 2023 | 302 | Italy | Cohort study | Database | 01/01/2021 to 31/12/2021 | All patients resident in the territory covered by ASL Foggia and who received at least one dose of Alirocumab or Evolocumab during the period of interest of this study (from January 2021 to December 2021) | moAbs (A&E) | - Implementation | Adherence assessed via MPR  Adherent: MPR ≥ 80% |  |
| 39 | Mulder 2023 | 65 | Netherlands | Cohort study | Outpatient specialty clinics | 02/01/2022 to 31/03/2023 | All consecutive patients ≥18 years who started PCSK9 inhibiting therapy as part of regular care at the outpatient lipid clinic of Erasmus MC university hospital in Rotterdam between February 2022 and March 2023. PCSK9 inhibiting therapy consisted of either PCSK9 mAb (alirocumab or evolocumab) or PCSK9 siRNA (inclisiran), and fufilled the Dutch reimbursement criteria for PCSK9 inhibitor | siRNA (I) | - Persistence | Non-persistent: gap not specified |  |
| 40 | Muntner 2024 | 16588 | United States | Cohort study | Database | 07/01/2015 to 31/12/2019 | US patients aged 21–64 (MarketScan) or ≥65 (Medicare) with continuous inpatient, outpatient, and pharmacy coverage for 365 days before and after the index date | moAbs (A&E) | - Implementation - Persistence | Adherence assessed via PDC  Adherent: PDC ≥ 80%  Non-persistent: gap ≥ 60 days |  |
| 41 | Nanchen 2022 | 100 | Switzerland | Cohort study | Outpatient specialty clinics | 07/01/2017 to 31/01/2019 | Between July 2017 and January 2019, ECARA enrolled adults (≥18 years) with ASCVD or high/very high cardiovascular risk who were receiving or planned to receive evolocumab per Swissmedic approved label and Swiss Atherosclerosis Association (AGLA) recommendations, on maximally tolerated statins or were statin-intolerant, and provided written informed consent | moAbs (E) | - Implementation - Persistence | Adherence reported as %  Non-persistent: gap not specified |  |
| 42 | Naoum 2024 | 503 | Israel | Cohort study | Database | 03/01/2022 to  30/11/2023 | Patients initiating inclisiran therapy between the period of March 2022 (following initial regulatory authorization in Israel) and November 2023. The patients were all members of the Clalit Health Services (CHS), the largest health care organization in Israel, which provides inclusive health care for about half of the country population, for whom we had full access to patients’ electronic data | siRNA (I) | - Persistence | Non-persistent: gap ≥ 60 days |  |
| 43 | Oren 2019 | 96 | United States | Cohort study | Outpatient specialty clinics | 09/01/2015 to 31/12/2018 | All patients who were referred to preventive cardiology at the Mayo Clinic (Minnesota) between September, 2015 and December, 2018 for management of severe dyslipidaemia | moAbs (A&E) | - Initiation | No. starting / No. approved |  |
| 44 | Parhofer 2019 | 612 | Germany | Cohort study | Outpatient specialty clinics | 11/01/2015 to 31/10/2016 | Patients who were eligible for the study had elevated LDL-C ≥1.8 mol/L (≥70 mg/dL; for patients with established cardiovascular disease) or ≥2.6 mmol/L (≥100 mg/dL; for patients without established cardiovascular disease) despite maximally tolerated non-alirocumab LLT, and their treating physicians decided to use alirocumab according to the summary of product characteristics | moAbs (A) | - Persistence | Non-persistent: gap not specified |  |
| 45 | Piccinni 2019 | 266 | Italy | Cohort study | Database | 07/01/2017 to 31/06/2018 | Individuals with at least one PCSK9 inhibitor dispensing with ATC code of evolocumab (C10AX13) or alirocumab (C10AX14) between July 2017 and June 2018, and at least two years in the inhabitant registry | moAbs (A&E) | - Implementation - Persistence - Reinitiation | Adherence assessed via PDC  Adherent: PDC ≥ 75%  Non-persistent: gap ≥ 30 days |  |
| 46 | Rallidis 2020 | 141 | Greece | Cohort study | Outpatient specialty clinics | NA | Patients who attended the lipid clinic of 3 large University general hospitals in Greece and started using PCSK9 | moAbs (A&E) | - Persistence | Non-persistent: gap not specified |  |
| 47 | Ray 2023 | 1951 | Austria, Belgium, Bulgaria, Czech Republic, Germany, Greece, Italy, Portugal, Slovakia, Spain, Sweden, Switzerland | Cohort study | Mixed | 08/01/2015 to 30/06/2021 | Adults aged ≥18 years who had their first prescription and received at least one dose of evolocumab as part of their clinical man­agement after 1 August 2015 | moAbs (E) | - Persistence | Non-persistent: permanently stopped therapy during the observation period |  |
| 48 | Reynolds 2019 | 287 | United States | Cohort study | Outpatient specialty clinics | 09/01/2015 to 12/01/2016 | Patients prescribed a PCSK9 inhibitor between September 1, 2015, and December 1, 2016 by a VUMC prescriber whose prescription was sent to the VSP lipid team within the study period | moAbs (A&E) | - Initiation | No. starting / No. approved |  |
| 49 | Rymer 2020 | 6151 | United States | Cohort study | Database | 07/01/2015 to 31/12/2017 | We restricted the analysis to patients initiating a PCSK9i, identified using National Drug Codes, from July 2015 through December 2017 | moAbs (A&E) | - Persistence - Reinitiation | Non-persistent: gap ≥ 30 days |  |
| 50 | Saborowski 2018 | 38 | Germany | Cohort study | Outpatient specialty clinics | NA to 30/06/2017 | Thirty-eight patients from the lipid outpatient clinic of the Hanover Medical School that had been treated with a PCSK9 inhibitor (alirocumab 75/150 mg or evolocumab 140 mg) until present | moAbs (A&E) | - Implementation | Adherence reported as % |  |
| 51 | Sheng 2024 | 4022 | Japan | Cohort study | Database | 21/04/2016 to 30/11/2021 | Patients who had at least one record of evolocumab and aged ≥15 years on the date of initial evolocumab treatment | moAbs (E) | - Persistence | Non-persistent: gap ≥ 60 days |  |
| 52 | Stoekenbroek 2017 | 238 | Netherlands | Cohort study | Database | NA to 30/06/2017 | Patients who initially participated in a clinical trial and started using alirocumab or evolocumab in routine care up to June 2017 were identified from the electronic hospital system of the Academic Medical Center (AMC) in Amsterdam | moAbs (A&E) | - Persistence | Non-persistent: gap not specified |  |
| 53 | Stummer 2023 | 7302 | Austria | Cohort study | Database | 09/01/2015 to 31/12/2020 | All patients, to whom at least one PCSK9i prescription was dispensed in extramural settings and reimbursed by Austrian Social Insurances during the study period (September 2015 - December 2020) | moAbs (A&E) | - Implementation - Persistence - Reinitiation | Adherence assessed via PDC  Adherent: PDC ≥ 80%  Non-persistent: gap ≥ 60 days |  |
| 54 | Svensson 2024 | 2341 | Sweden | Cohort study | Database | 07/01/2015 to 31/05/2020 | All residents of Sweden who had at least one prescription for evolocumab (ATC code: C10AX13) filled between 1 July 2015 and 31 May 2020 | moAbs (E) | - Implementation - Persistence | Adherence assessed via PDC  Adherent: PDC ≥ 80%  Non-persistent: gap ≥ 56 days |  |
| 55 | Tai 2018a | 222 | Germany,  Spain,  UK | Cohort study | Survey | 12/01/2016 to 30/04/2017 | The DSP is a unique point-in-time survey which was conducted in Germany, Spain, and the UK from December 2016 through April 2017 to collect physician-reported information on treatment of dyslipidemia, including detailed data on comorbidity and prescription lipid lowering therapies (LLT). A geographically representative sample of physicians in each country was identified from publicly available lists of healthcare professionals. Potential participants were recruited by field-based interviewers and screened for study eligibility prior to collection of patient-specific data | moAbs (A&E) | - Implementation | Adherence reported as % |  |
|  | Tai 2018b | 97 |  |  |  |  |  |  |  |  |  |
|  | Tai 2018c | 68 |  |  |  |  |  |  |  |  |  |
| 56 | Vicente-Valor 2021 | 115 | Spain | Cohort study | Database | 02/01/2017 to 30/04/2020 | All patients initiated treatment with alirocumab 75 mg (scalable to 150 mg) or evolocumab 140 mg once every two weeks, according to the conditions established by National Health System (patients with LDL-C > 100 mg and CVD and/or FH, tolerant or intolerant to statins) | moAbs (A&E) | - Implementation - Persistence | Adherent: Adherence ≥ 90%  Non-persistent: discontinued therapy prior to the cutoff date for analysis |  |
| 57 | Warden 2021 | 89 | United States | Cohort study | Outpatient specialty clinics | 05/01/2019 to 31/05/2020 | Adult patients from the CPC who received a new prescription for a PCSK9 inhibitor (alirocumab or evolocumab) for the first time between May 2019 and May 2020 | moAbs (A&E) | - Initiation - Implementation - Persistence | Initiation: No. starting / No. prescribed  Adherence reported as %  Non-persistent: gap not specified |  |
| 58 | Wong 2023 | 3162 | United States | Cohort study | Database | 01/01/2019 to 31/12/2019 | Continuous enrolment in a health plan for at least 12 months before and 6 months after the index date; having at least one filled prescription of PCSK9i prior to index date; no switching of NDCs or drugs during the 6-month post-index period; aged ≥18 years; and having no missing age, payer type, plan type or region of residence data | moAbs (A&E) | - Implementation | Adherence assessed via PDC |  |
| 59 | Zafrir 2018 | 101 | Israel | Cohort study | Outpatient specialty clinics | 01/01/2016 to 31/11/2017 | All patients at a regional lipid clinic of Lady Davis Carmel Medical Center in Haifa, Israel, who were prescribed PCSK9 monoclonal antibodies (subcutaneous injection of alirocumab 75/150 mg or evolocumab 140 mg every 2 weeks | moAbs (A&E) | - Initiation - Persistence | Initiation: No. starting / No. approved  Non-persistent: gap not specified |  |
| 60 | Zafrir 2020 | 1600 | Israel | Cohort study | Database | 01/01/2016 to 31/12/2019 | Patients initiating treatment with PCSK9 monoclonal antibodies (alirocumab or evolocumab) between the period of January 2016 and December 2019 | moAbs (A&E) | - Implementation - Persistence - Reinitiation | Adherence assessed via PDC  Adherent: PDC ≥ 80%  Non-persistent: gap ≥ 60 days |  |
| **Conference abstracts or brief reports** | | | | | | | | | | | |
| 61 | Ali 2024 | 204 | United Arab Emirates | Cohort study | Outpatient specialty clinics | 26/07/2021 to 31/12/2023 | Clinic patients that were prescribed inclisiran from July 26, 2021 - December 31, 2023 | siRNA (I) | - Initiation - Persistence | Initiation: No. starting / No. prescribed  Non-persistent: gap ≥ 90 days |  |
| 62 | Bajaj 2018 | 235 | Canada | Cohort study | Outpatient specialty clinics | NA | Atherosclerotic heart disease patients on maximally tolerated statin therapy with an LDL > 2.5 mmol/L who were prescribed the PCSK9 inhibitor evolocumab at a cardiac prevention facility in Cambridge, Canada | moAbs (E) | - Initiation | No. starting / No. prescribed |  |
| 63 | Bartsch 2024 | 26 | United States | Cohort study | Outpatient specialty clinics | 04/01/2022 to 31/10/2023 | Patients received at least one dose of inclisiran at the lipid clinic | siRNA (I) | - Initiation - Implementation | Initiation: No. starting / No. prescribed  Adherence assessed as proportion (%) of patients received doses at specific time points |  |
| 64 | Buckley 2019 | 24 | Ireland | Cohort study | Database | 01/01/2015 to NA | All patients treated locally with PCSK9 inhibitors | moAbs (A&E) | - Initiation - Persistence | Initiation: No. starting / No. prescribed  Non-persistent: gap not specified |  |
| 65 | Cadenas Chamorro 2018 | 36 | Spain | Cohort study | Database | 05/01/2016 to 31/10/2017 | Every patient who was prescribed Alirocumab or Evolocumab in a regional hospital with a target population of 300000 inhabitants in northern Madrid (Spain) from May 2016 to October 2017 | moAbs (A&E) | - Initiation - Persistence | Initiation: No. starting / No. prescribed  Non-persistent: gap not specified |  |
| 66 | Cancela Diez 2018 | 30 | Spain | Cohort study | Database | 01/01/2016 to 31/05/2017 | All patients who received evolocumab during 2016 and until May 2017 | moAbs (E) | - Persistence | Non-persistent: gap not specified |  |
| 67 | Chugh 2019 | 170 | United States | Cohort study | Outpatient specialty clinics | 07/01/2016 to 31/08/2018 | Lipid clinic patients that were identified as appropriate PCSK9i candidates with acquired insurance coverage from July 2016 to August 2018 | moAbs (A&E) | - Persistence | Non-persistent: gap not specified |  |
| 68 | Claramunt Garcia 2020 | 82 | Spain | Cohort study | Database | NA to 30/09/2019 | Patients treated with anti-PCSK9 who attended the pharmacy service of a third level hospital for a consultation | moAbs (A&E) | - Implementation | Adherence assessed via MPR  Adherent: MPR ≥ 80% |  |
| 69 | DallaValle 2020 | 33 | Italy | Cohort study | NA | NA | Patients treated with PCSK9i with HeFH or primary hypercholesterolaemia and with very high cardiovascular risk | moAbs (A&E) | - Persistence | Non-persistent: gap not specified |  |
| 70 | Desai 2023 | 562 | United States | Cohort study | Outpatient specialty clinics | 03/01/2022 to 31/11/2022 | Those who initiated inclisiran from March 1, 2022 to November 31, 2022 at MICs | siRNA (I) | - Implementation | Adherence assessed as proportion (%) of patients received doses at specific time points |  |
| 71 | Dominguez Bachiller 2020 | 46 | Spain | Cohort study | Database | 03/01/2016 to 31/09/2019 | Every patient treated with alirocumab and evolocumab between March 2016 and September 2019 | moAbs (A&E) | - Persistence | Non-persistent: gap not specified |  |
| 72 | Gao 2023 | 61 | China | Cohort study | NA | 23/12/2019 to 24/06/2022 | Chinese IS and TIA inpatients who initiated PCSK9mAb therapy between December 23, 2019, and June 24, 2022 | moAbs (A&E) | - Implementation - Persistence | Adherence assessed via PDC  Adherent: PDC ≥ 80% Non-persistent: gap not specified |  |
| 73 | Ghazi 2023 | 16588 | United States | Cohort study | Database | 07/01/2015 to 31/12/2019 | Adults initiating a PCSK9mAb or ezetimibe from Medicare and MarketScan commercial health insurance databases between 7/2015 and 12/2019 | moAbs (A&E) | - Implementation - Persistence | Adherence reported as %  Adherent: Adherence ≥ 80%  Non-persistent: gap ≥ 60 days |  |
| 74 | Gragnano 2018 | 39 | Italy | Cohort study | Database | 02/01/2016 to 31/07/2017 | Patients at high cardiovascular risk, admitted to the cardiology department of University of Campania, Luigi Vanvitelliâ, A.O. dei Colli Monaldi Hospital of between February 2016 and July 2017 | moAbs (A&E) | - Implementation | Adherence reported as %  Adherent: Adherence ≥ 80% |  |
| 75 | Han 2021 | 47 | United Kingdom | Cohort study | Outpatient specialty clinics | 10/01/2017 to 31/08/2019 | Patients initiated on PCSK9 inhibitor therapy in lipid clinic at Royal Lancaster Infirmary from October 2017 to August 2019 | moAbs (A&E) | - Implementation - Persistence | Adherent: Patients with LDL-C and total cholesterol data collected at lipid clinic after 3 months of PCSK9 inhibitor  Non-persistent: gap not specified |  |
| 76 | Heintjes 2019 | 268 | Netherlands | Cohort study | Database | 04/01/2016 to 31/03/2018 | Patients who received a first prescription for PCSK9-i (biweekly evolocumab 140mg, alirocumab 75mg or 150mg) between April 2016 and March 2018 | moAbs (A&E) | - Persistence | Non-persistent: gap not specified |  |
| 77 | Klebs 2022 | 334 | Germany | Cohort study | Database | 02/01/2021 to 31/10/2021 | For the adherence and persistence analyses, a subgroup of patients with a first inclisiran prescription recorded between February and June 2021 was selected, allowing for at least 4 months follow-up, up to October 2021 | siRNA (I) | - Implementation | Adherence assessed as proportion (%) of patients received doses at specific time points |  |
| 78 | Knickelbine 2018 | 196 | United States | Cohort study | Outpatient specialty clinics | 09/01/2015 to 31/05/2017 | Patients who got prescribed PCSK9i from Sept 2015 to May 2017 at Minneapolis Heart Institute Foundation at Abbott Northwestern Hospital | moAbs (A&E) | - Persistence | Non-persistent: gap not specified |  |
| 79 | LorenaMartin Polo 2019 | 74 | Spain | Cohort study | Database | 10/01/2016 to 31/05/2018 | Patients with LDL-C >100mg/dl in secondary prevention or with proven FH despite optimal therapy (maximum tolerated statin dose and ezetimibe) or proven statin intolerance | moAbs (A&E) | - Initiation - Persistence | Initiation: No. starting / No. prescribed  Non-persistent: gap not specified |  |
| 80 | MerinoMartin 2018 | 30 | Spain | Cohort study | Database | 05/01/2016 to 31/08/2017 | Patients with LDL-C >100 mg/dL and HeFH or CVD treated with high doses of atorvastatin or rosuvastatin or patients with statins intolerance, who received prescriptions of PCSK9i in a general hospital from May 2016 until August 2017 | moAbs (A&E) | - Initiation - Implementation | Initiation: No. starting / No. prescribed  Adherent: threshold not specified |  |
| 81 | Niu 2023 | 42826 | United States | Cohort study | Database | 01/01/2021 to 08/02/2023 | Patients were aged ≥18 years, had 12 months of continuous enrolment before the index date, 6 months CE after the index date and a first claim for inclisiran, alirocumab, or evolocumab in the identification period (1/1/22 - 10/31/22) | moAbs (A&E)  siRNA (I) | - Implementation | Adherence assessed via PDC  Adherent: PDC ≥ 80% |  |
| 82 | Pandey 2018 | 126 | Canada | Cohort study | NA | NA | Patients prescribed PCSK9 inhibitors in high-risk patients with stable CAD, post -MI or post-revascularization (ASCVD patients), who were not at target LDL levels | moAbs (E) | - Initiation | No. starting / No. prescribed |  |
| 83 | Popadic 2024 | 36901 | United States | Cohort study | Database | 01/01/2021 to 31/08/2023 | Patients were ≥18 years, had 12 months of continuous enrolment before and after the index date and a first claim for inclisiran, alirocumab, or evolocumab between 1/1/22 - 8/31/22 | moAbs (A&E)  siRNA (I) | - Implementation - Persistence | Adherence assessed via PDC  Non-persistent: gap ≥ 60 days for moAbs; ≥ 90 for inclisiran |  |
| 84 | Rodriguez 2020 | 33 | Spain | Cohort study | Database | NA to 09/01/2017 | All patients starting alirocumab before September 2017 in our institution and treated for at least 96 weeks were included | moAbs (A) | - Implementation - Persistence | Adherence assessed via MPR  Non-persistent: gap not specified |  |
| 85 | Sammour 2021 | 65 | United States | Cohort study | Database | 01/01/1999 to 30/04/2020 | Patients who received a heart transplant from 1999 to 2019 and were started on PCSK9i at Saint Luke’s Mid America Heart Institute | moAbs (A&E) | - Initiation - Persistence | Initiation: No. starting / No. prescribed  Non-persistent: gap not specified |  |
| 86 | Sathiyakumar 2017 | 35 | United States | Cohort study | Outpatient specialty clinics | 01/01/2015 to 31/12/2016 | All patients prescribed PCSK9i in a tertiary care lipid clinic from Jan 1, 2015 to Dec 31, 2016 identified through chart review | moAbs (A&E) | - Initiation - Implementation | Initiation: No. starting / No. prescribed  Adherent: threshold not specified |  |
| 87 | Sbrana 2020 | 122 | Italy | Cohort study | NA | NA | Patients affected by primary hypercholesterolemia | moAbs (A&E) | - Persistence | Non-persistent: gap not specified |  |
| 88 | Smith 2019 | 81 | United States | Cohort study | Outpatient specialty clinics | NA | Patients approved compared and those not approved for a PCSK9i prescription in a real-world setting | moAbs (A&E) | - Initiation - Persistence | Initiation: No. starting / No. approved  Non-persistent: gap not specified |  |
| 89 | Snel 2022 | 239 | Belgium | Cohort study | Database | NA | Patients taking anti-PCSK9-mAb treatment followed up at our Lipid Clinic | moAbs (A&E) | - Persistence | Non-persistent: gap not specified |  |
| 90 | Starner 2016 | 57 | United States | Cohort study | Database | 08/01/2015 to 31/12/2015 | Commercial members subject to utilization management who had at least one paid or rejected PCSK9i transaction 8/1/15 through 12/31/15 | moAbs (A&E) | - Persistence | Non-persistent: gap ≥ 28 days |  |
| 91 | Waldmann 2020 | 238 | Germany | Cohort study | Outpatient specialty clinics | 01/01/2015 to 31/12/2018 | Individuals who presented to our lipid clinic with the indication for treatment with PCSK-9-i and an initial prescription at our clinic between 2015 and 2018 | moAbs (A&E) | - Persistence | Non-persistent: gap not specified |  |
| 92 | Weber 2019 | 126 | Netherlands | Cohort study | Outpatient specialty clinics | 01/02/2010 to 31/03/2019 | All patients prescribed a PCSK9 inhibitor at the LUMC Lipid Clinic | moAbs (A&E) | - Initiation - Persistence - Reinitiation | Initiation: No. starting / No. prescribed  Non-persistent: gap not specified |  |
| 93 | Wiener 2020 | 11958 | United States | Cohort study | Database | 08/01/2015 to 31/03/2019 | Adults initiating a PCSK9i from August 2015 to March 2019, with 12 months of continuous enrolment prior to PCSK9i initiation and at least 3 months of follow-up data | moAbs (A&E) | - Persistence | Non-persistent: gap not specified |  |
| 94 | Xiao 2023a | 4848 | China | Cohort study | Database | 01/01/2022 to NA | Adult patients who initiated an anti-PCSK9 mAb from January 1, 2022 with at least a 6-month follow-up were included and grouped into ASCVD and ASCVD high-risk subgroups according to their history and risk levels of ASCVD during the 6-month pre-index period | moAbs (A&E) | - Implementation | Adherence assessed via PDC  Adherent: PDC ≥ 80% |  |
|  | Xiao 2023b | 2700 |  |  |  |  |  |  |  |  |  |

**Abbreviations:** MPR, medication possession ratio; NA, not appliable; No. number; PDC, proportion of days covered.

* moAbs (A&E) = Monoclonal antibodies (alirocumab and evolocumab)

moAbs (A) = Monoclonal antibodies (alirocumab) only
 moAbs (E) = Monoclonal antibodies (evolocumab) only
 siRNA (I) = Small interfering RNA (inclisiran) only
Note: Cohorts reported within a study are indicated by a lowercase letter following the publication year.

# Boxplots of Baseline Population Characteristics

**Abbreviations:** MPR, medication possession ratio; PDC, proportion of days covered.
**Figure S5A.** Proportion of female study participants (%)


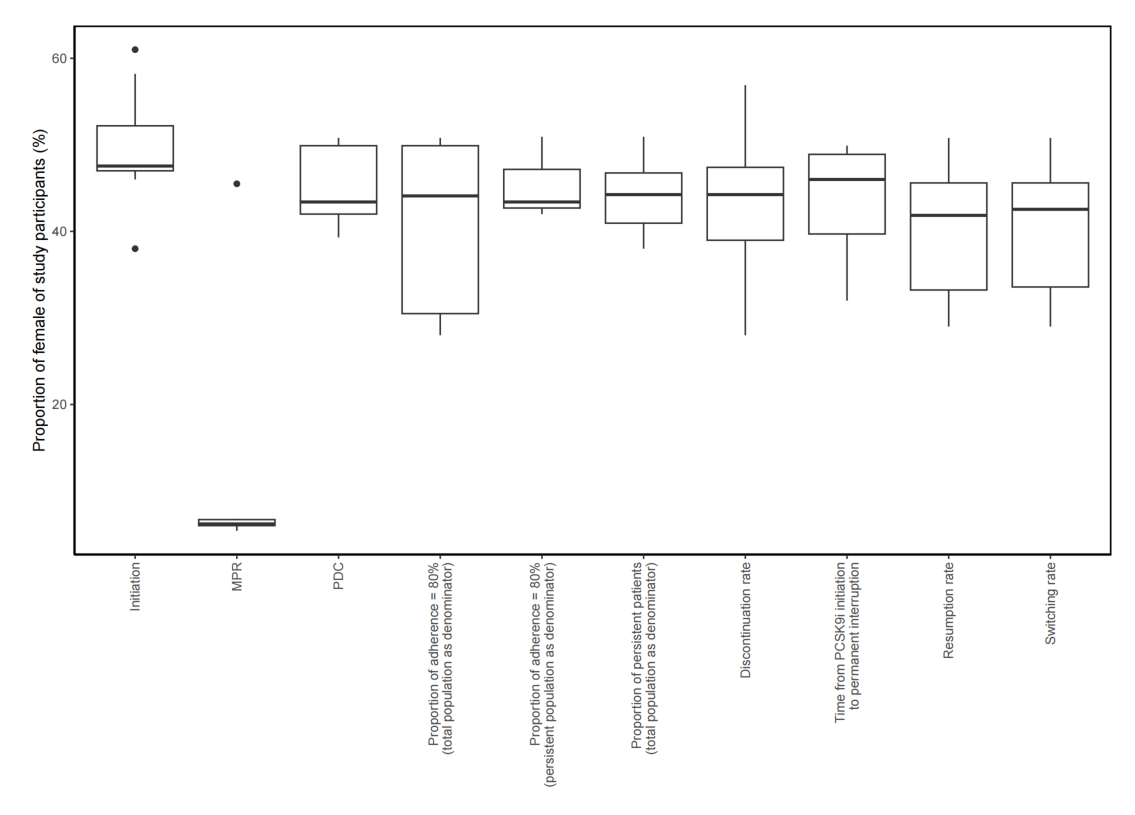


**Figure S5B.** Proportion of study participants with smoking history (%)


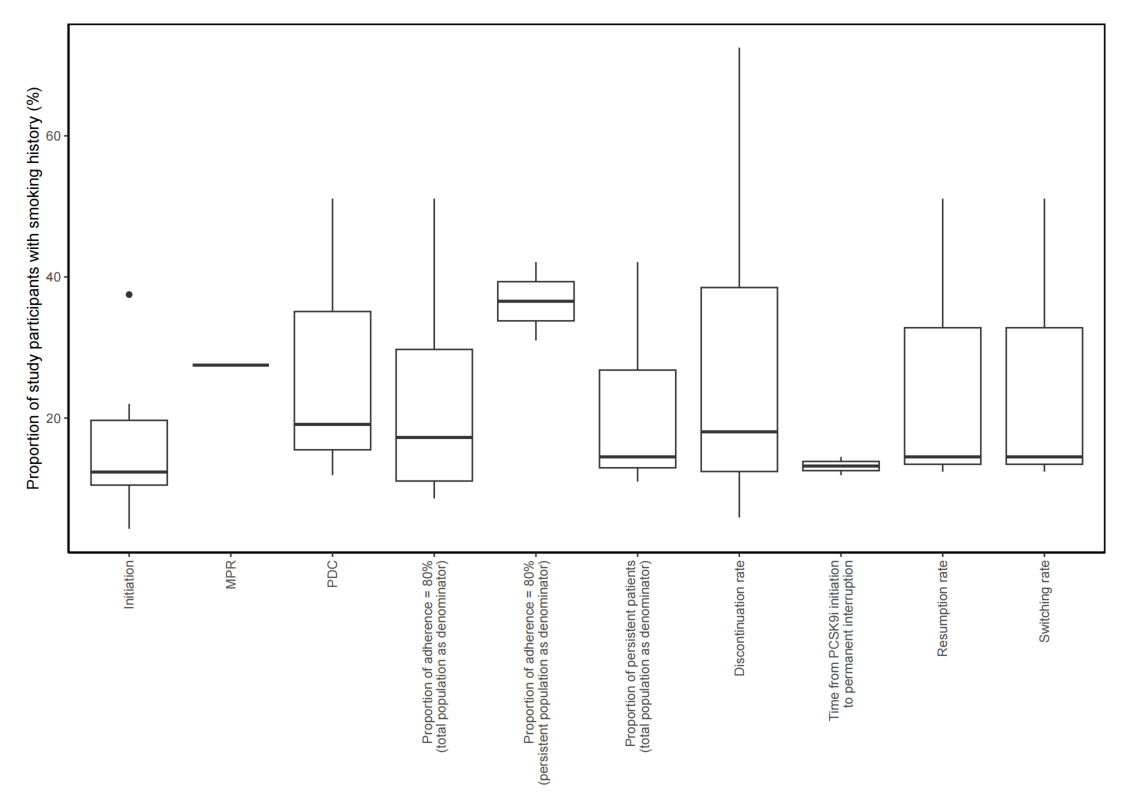


**Figure S5C.** Proportion of study participants with familial hypercholesterolaemia (%)


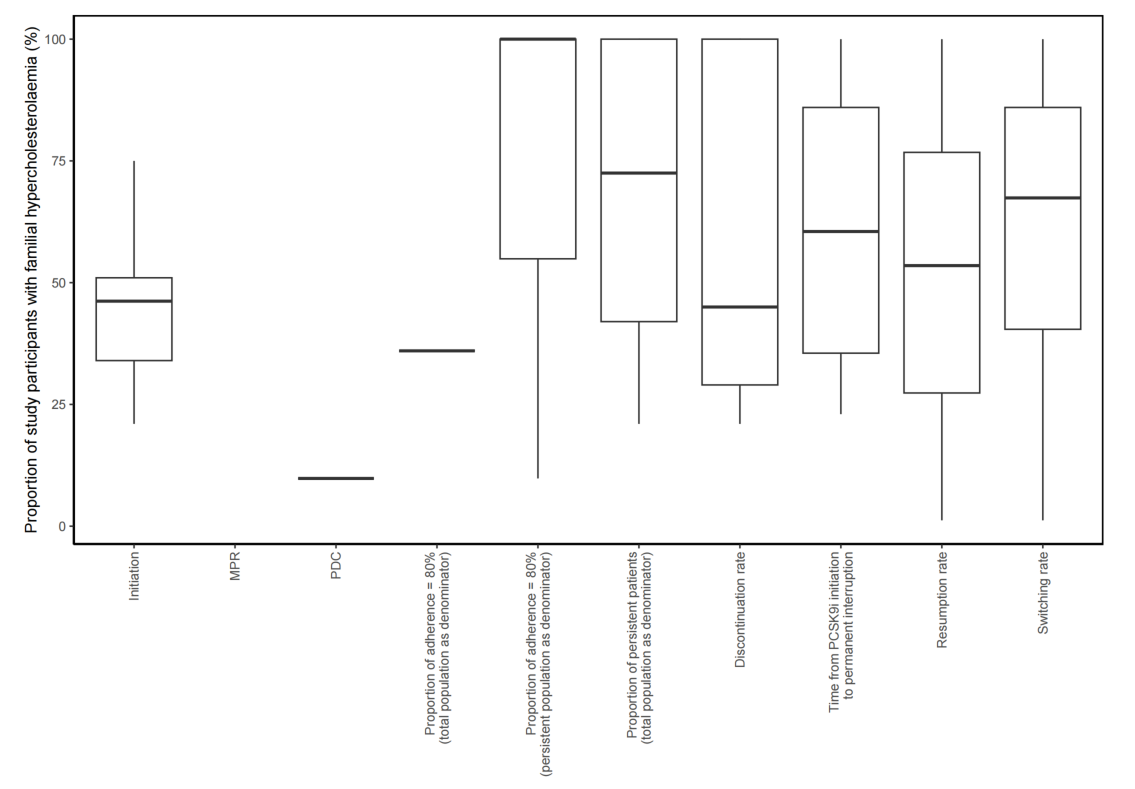


**Figure S5D.** Proportion of study participants with atherosclerotic cardiovascular disease (%)


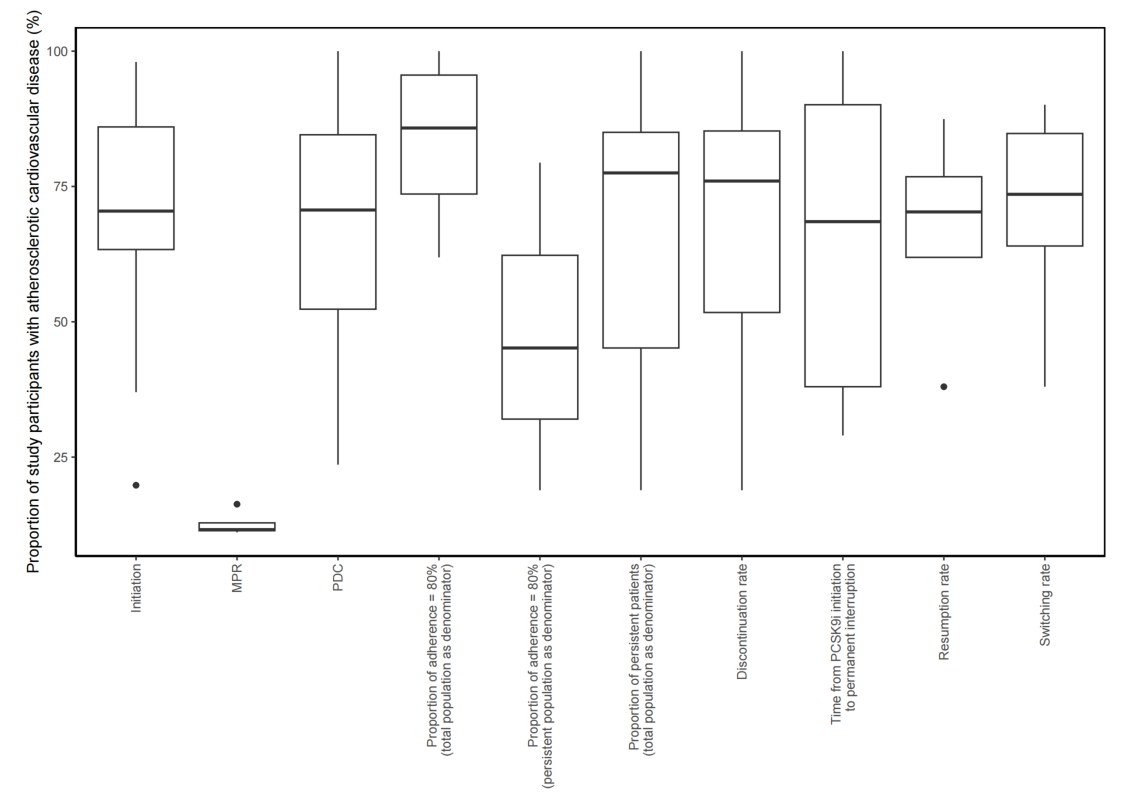


**Figure S5E.** Proportion of study participants with statin intolerance (%)


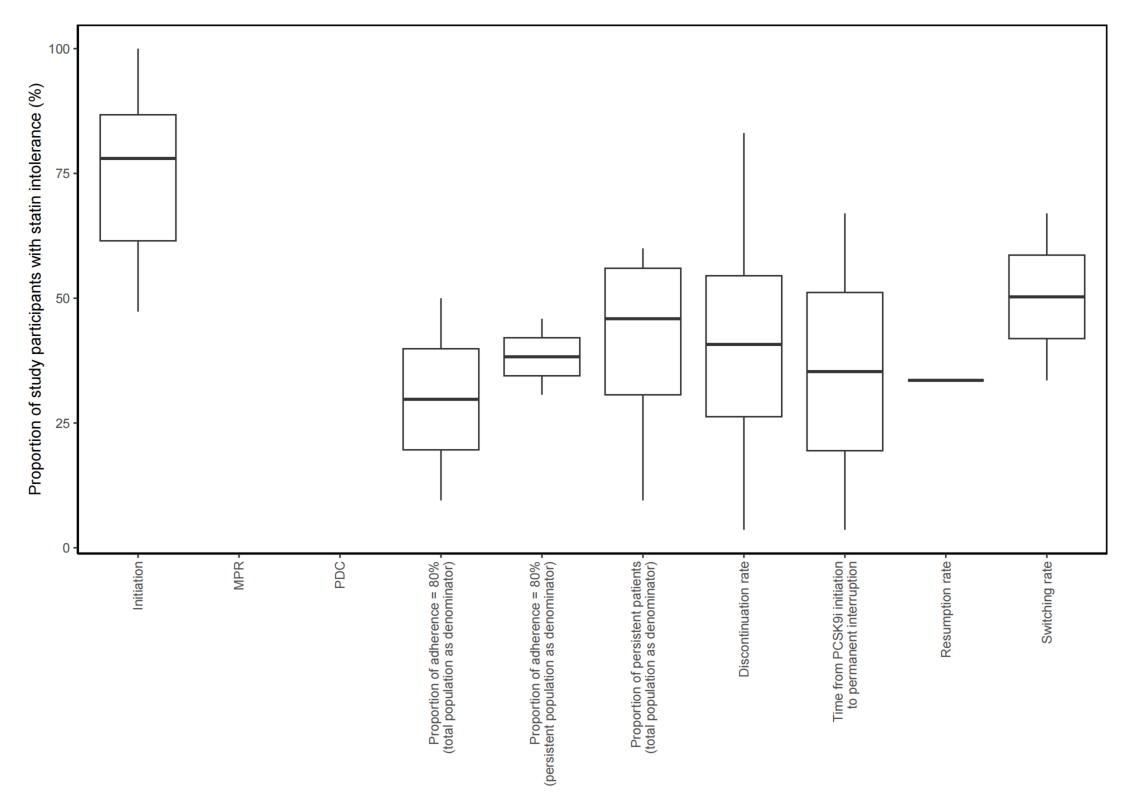


**Figure S5F.** Proportion of study participants with diabetes (%)


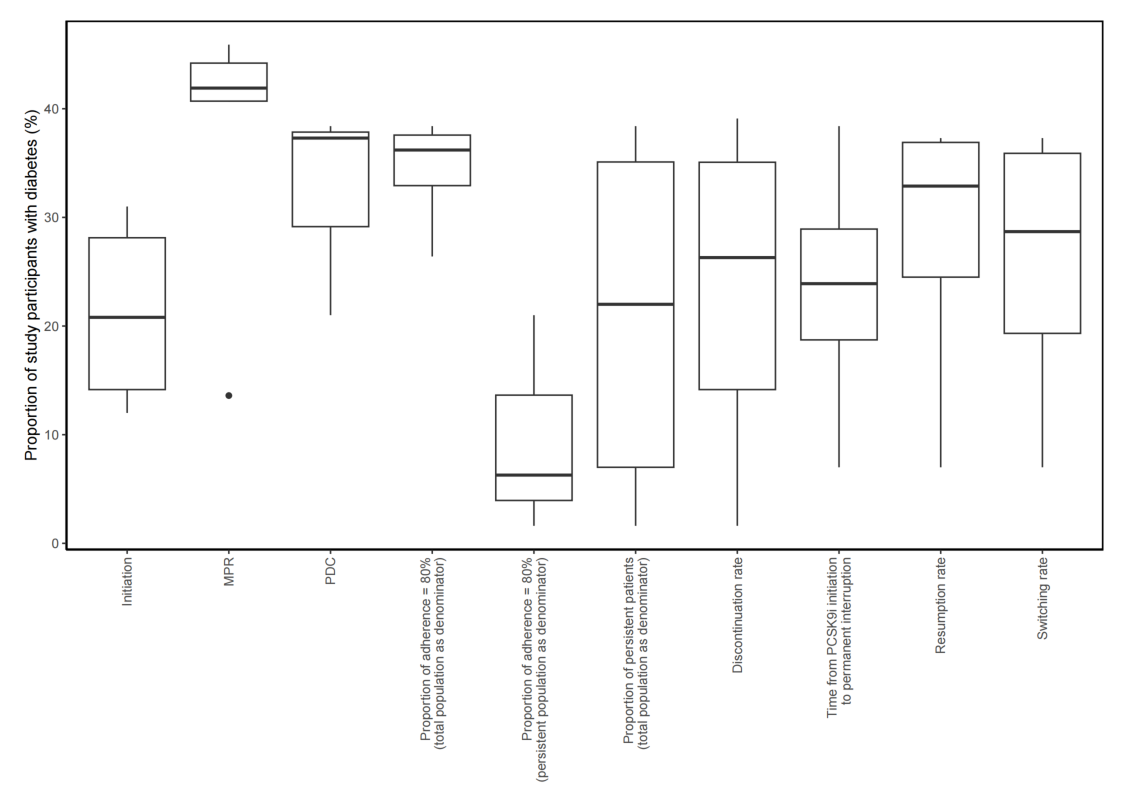


**Figure S5G.** Proportion of study participants with hypertension (%)


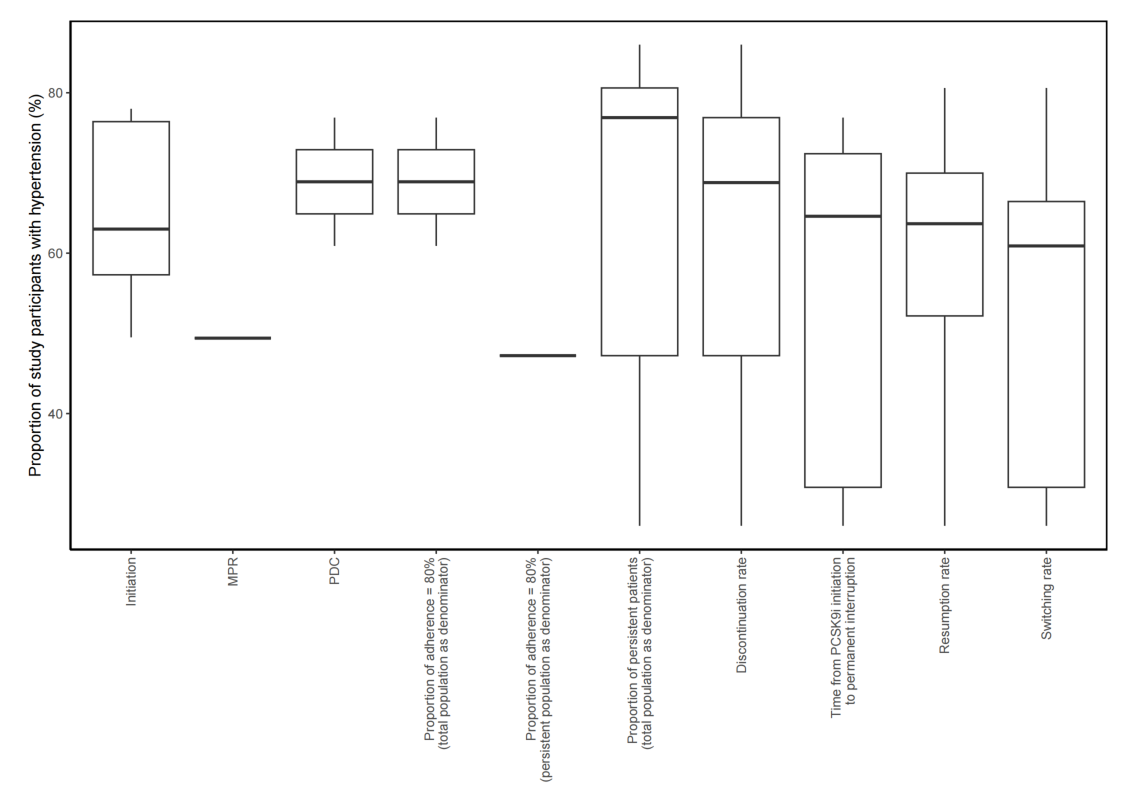


**Figure S5H.** Proportion of study participants with lipid-lowering therapies (%)


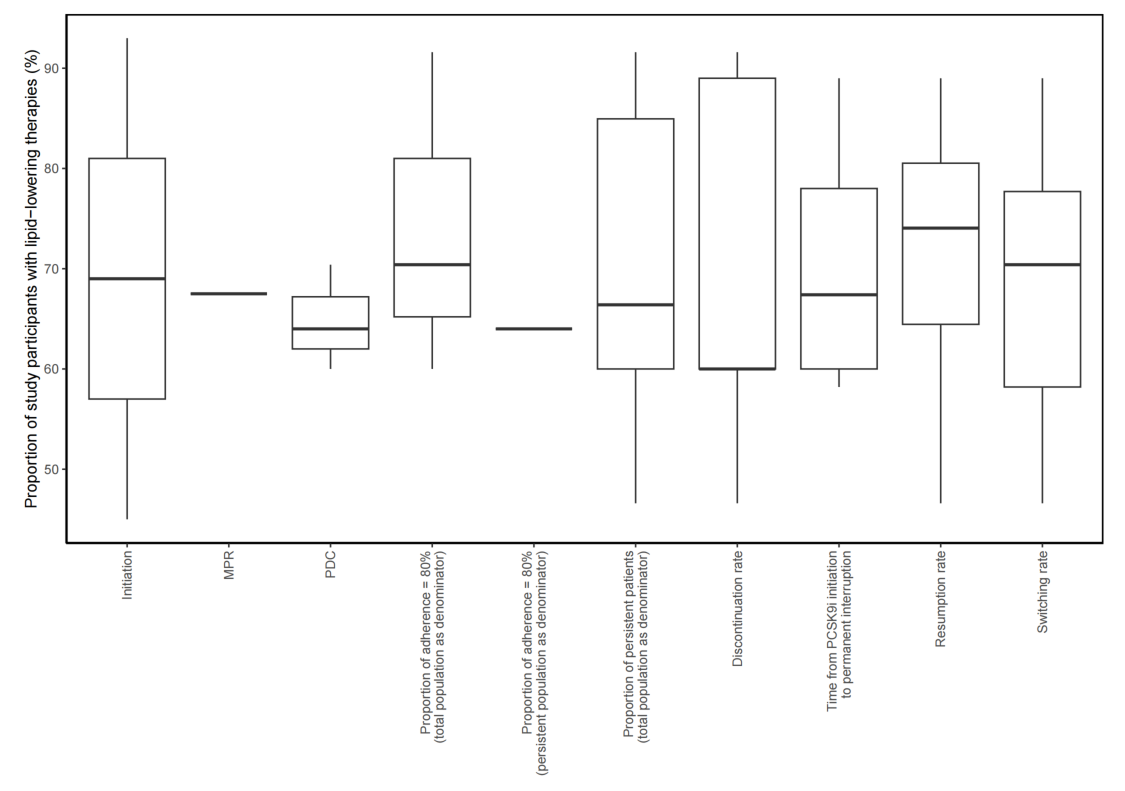


**Figure S5I.** Proportion of study participants with both statin and ezetimibe (%)


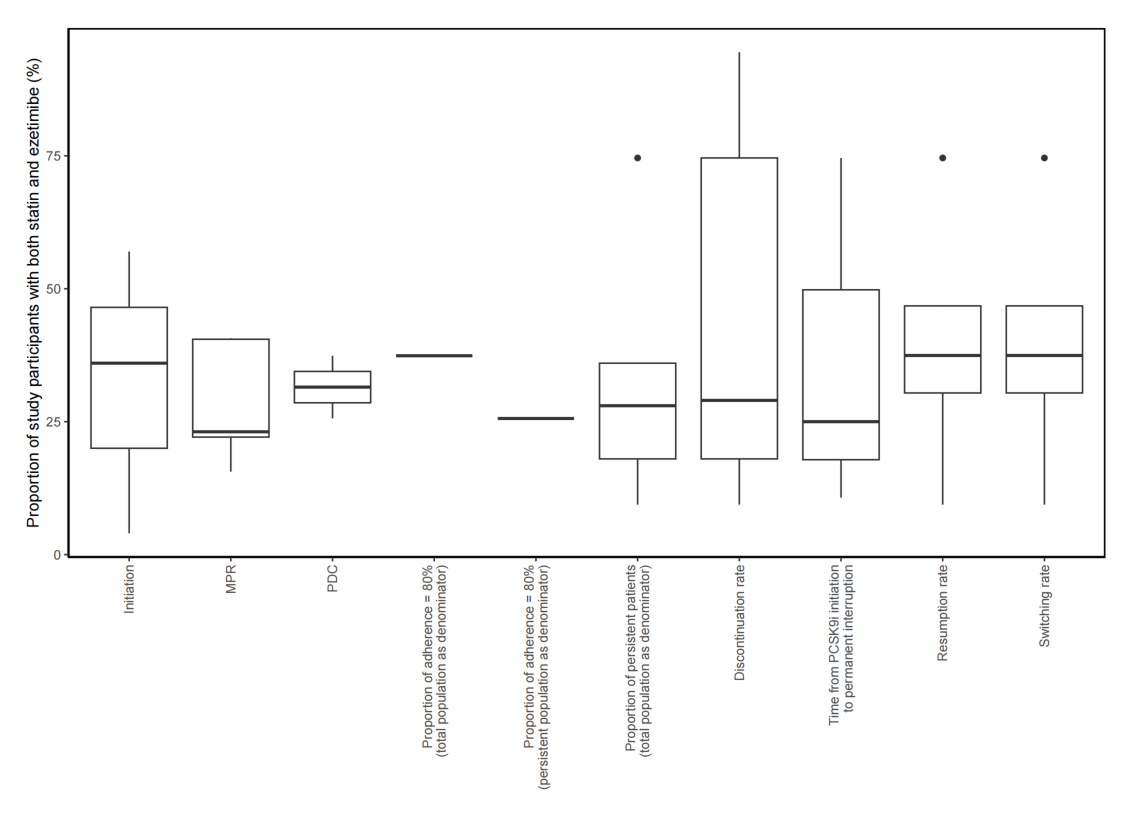


**Figure S5J.** Proportion of study participants with statin (%)


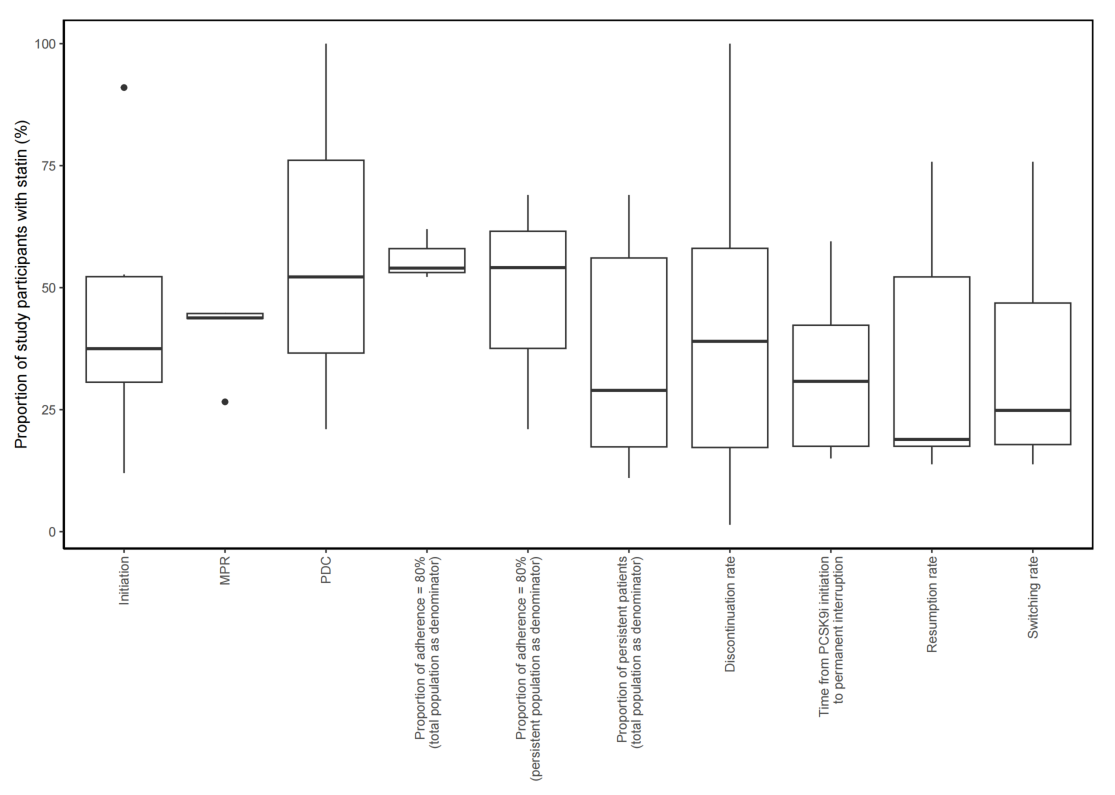


**Figure S5K.** Proportion of study participants with ezetimibe (%)


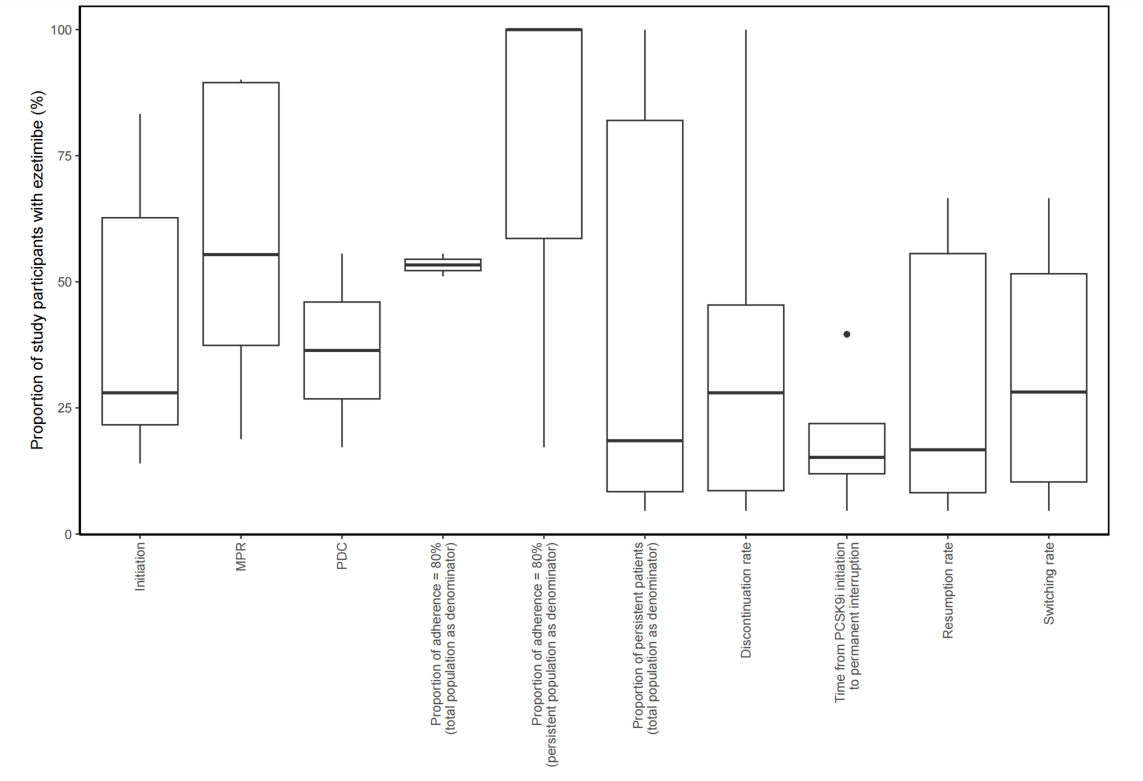


**Figure S5L.** Mean age (years)


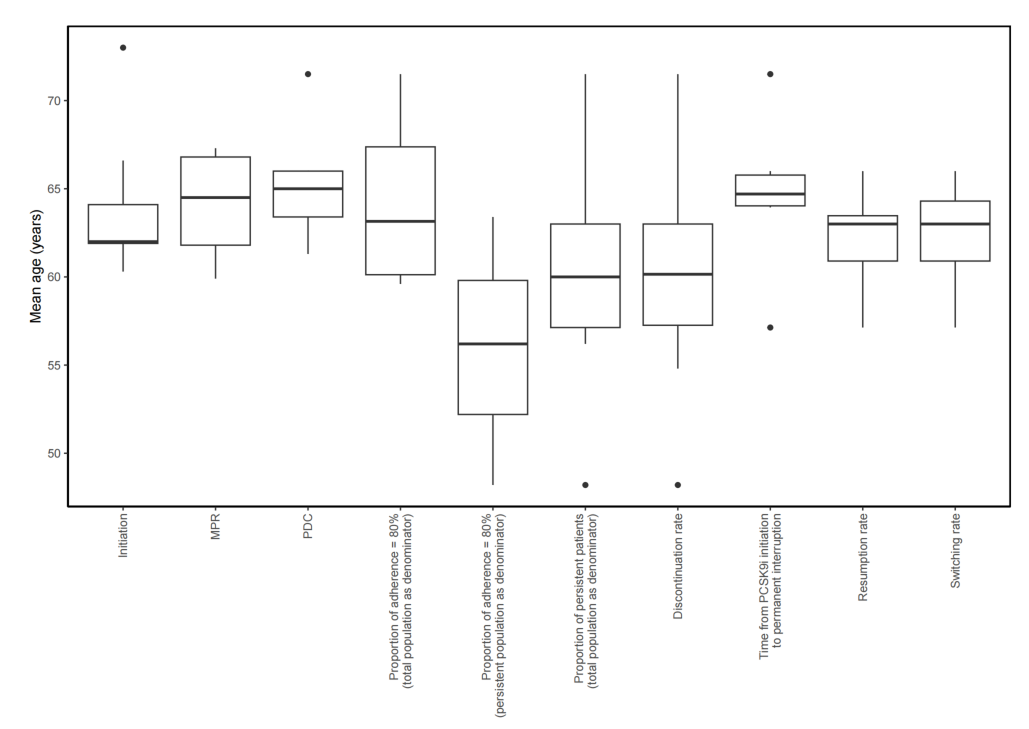


**Figure S5M.** Mean body mass index (BMI) (kg/m2)


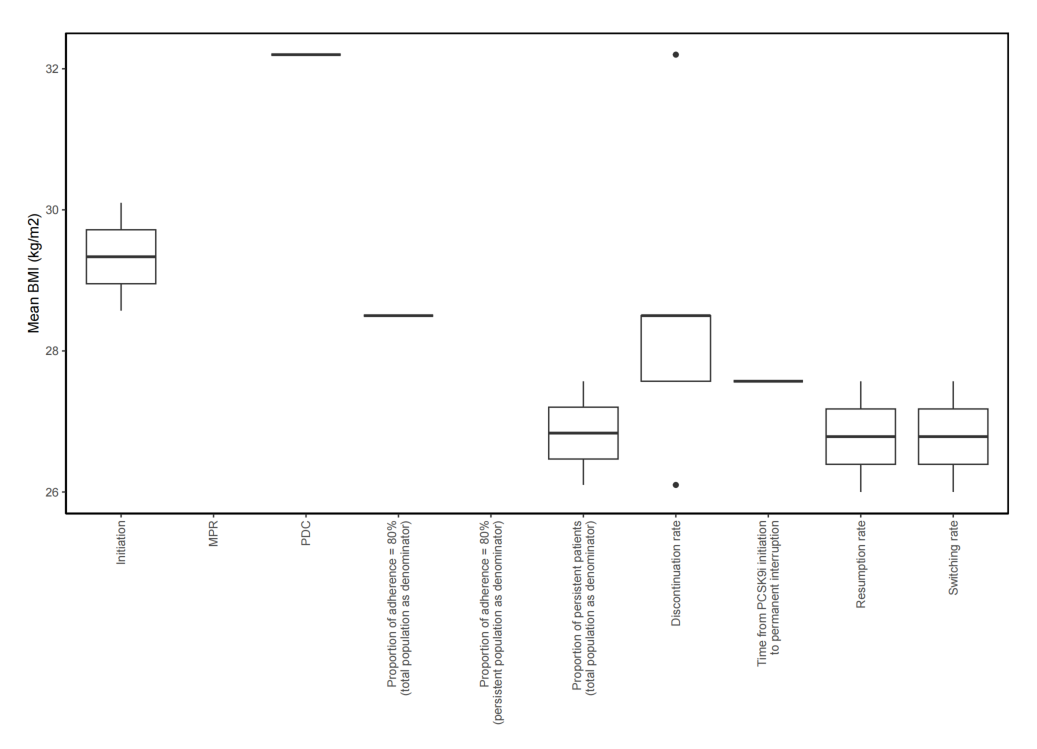


**Figure S5N.** Mean total cholesterol (TC) (mg/dL)


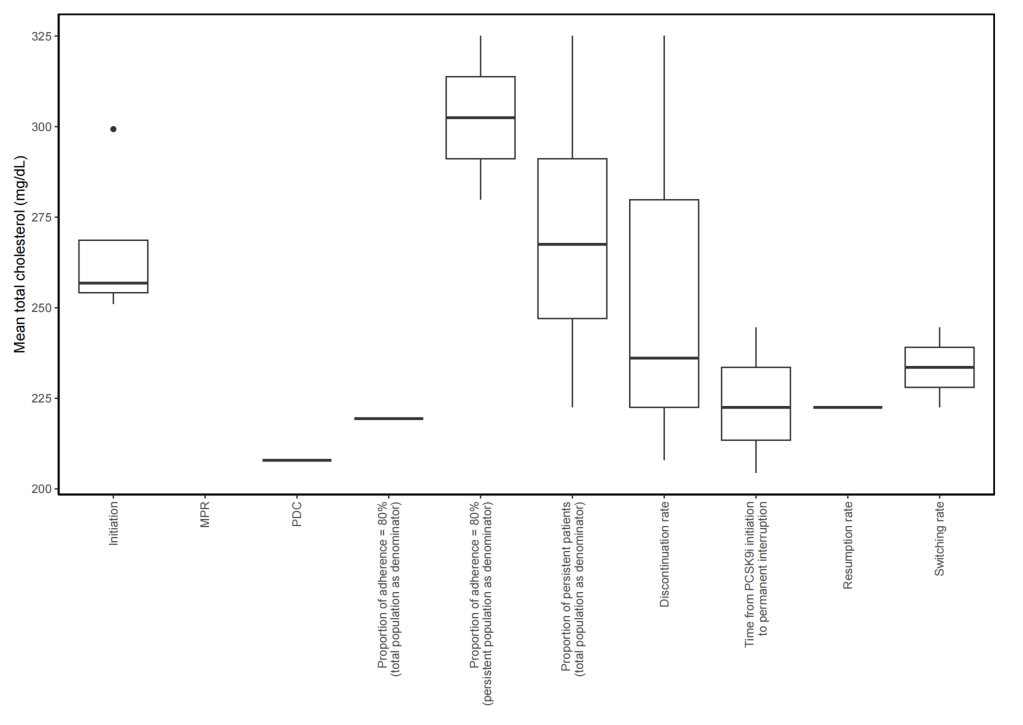


**Figure S5O.** Mean low-density lipoprotein cholesterol (LDL-C) (mg/dL)


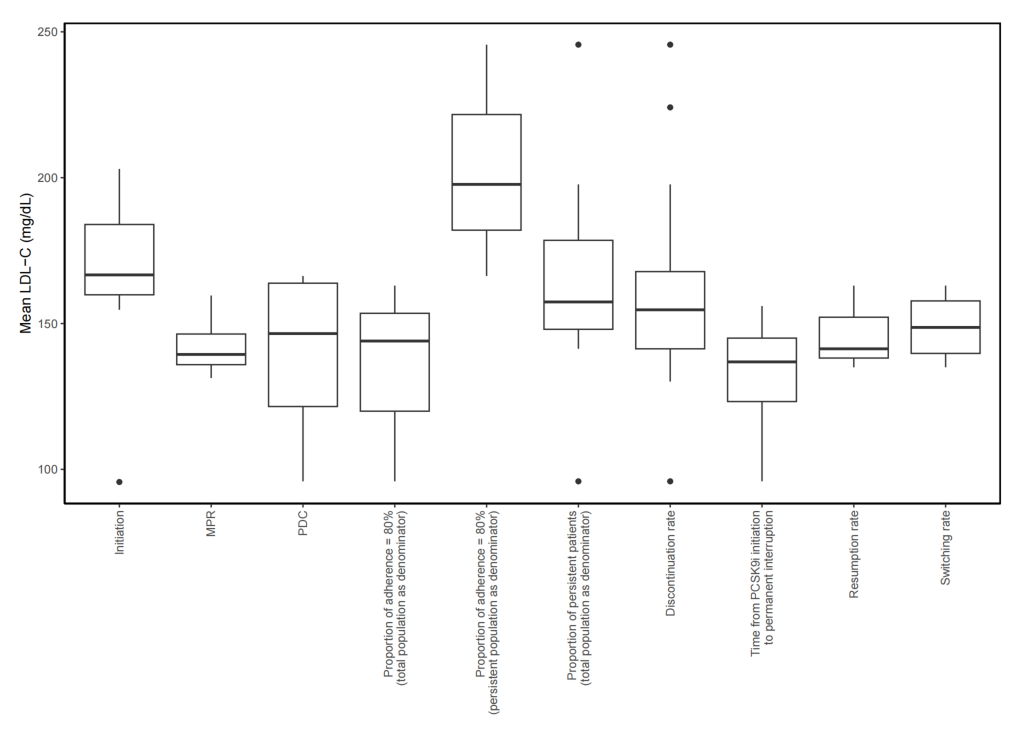


**Figure S5P.** Mean non-high-density lipoprotein cholesterol (non-HDL-C) (mg/dL)


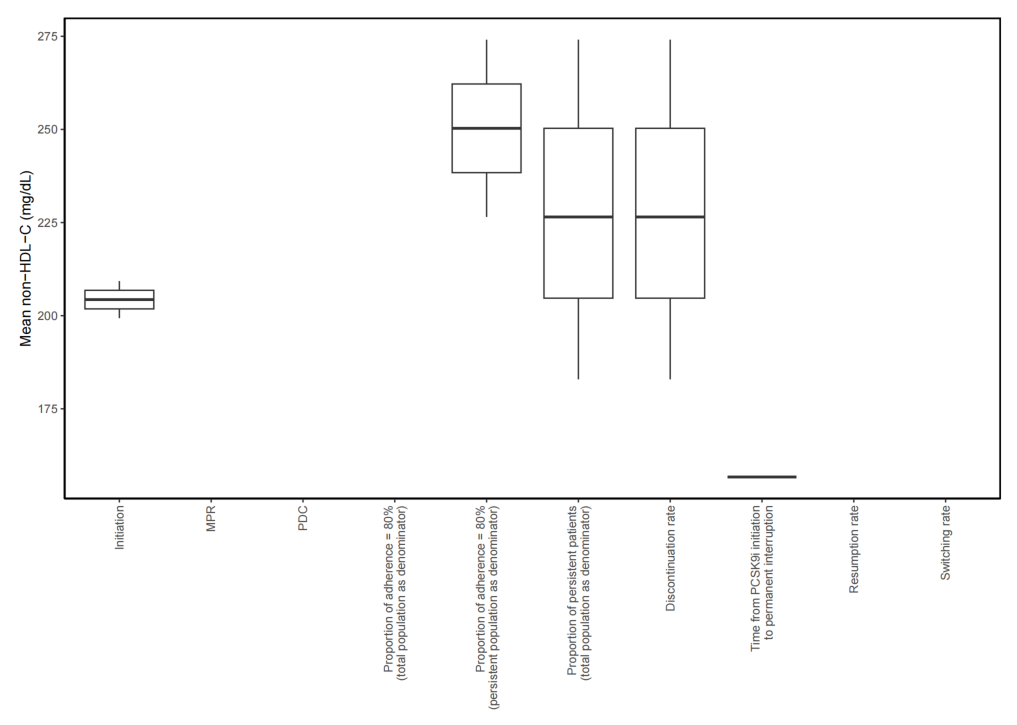


**Figure S5Q.** Mean high-density lipoprotein cholesterol (HDL-C) (mg/dL)


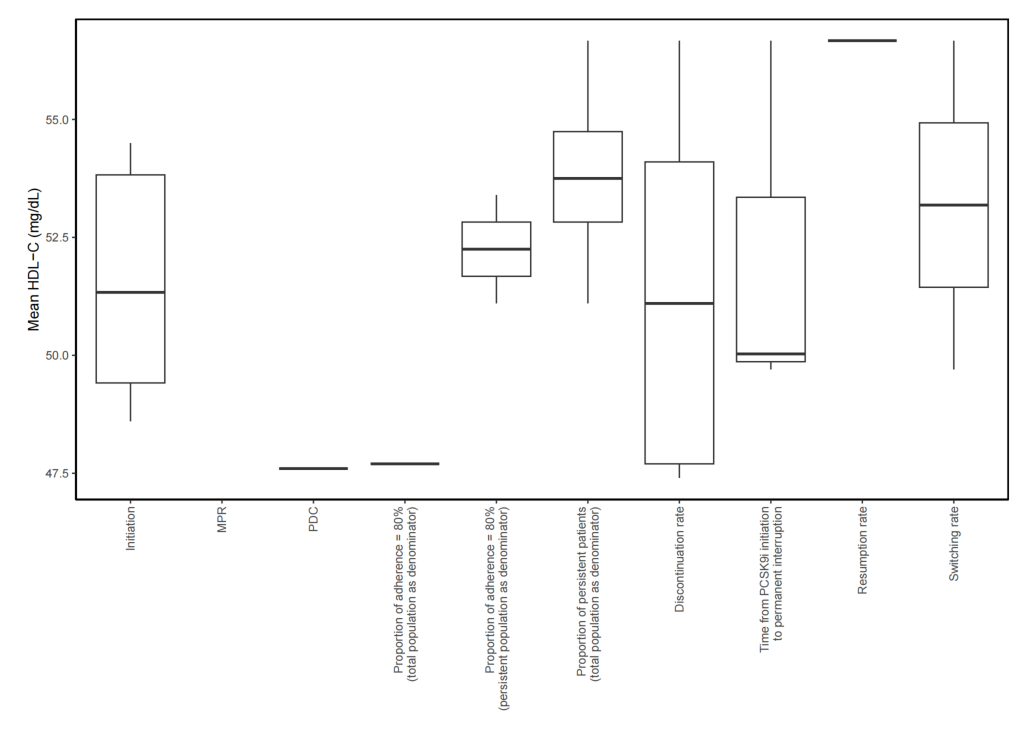


**Figure S5R.** Mean triglycerides (TG) (mg/dL)


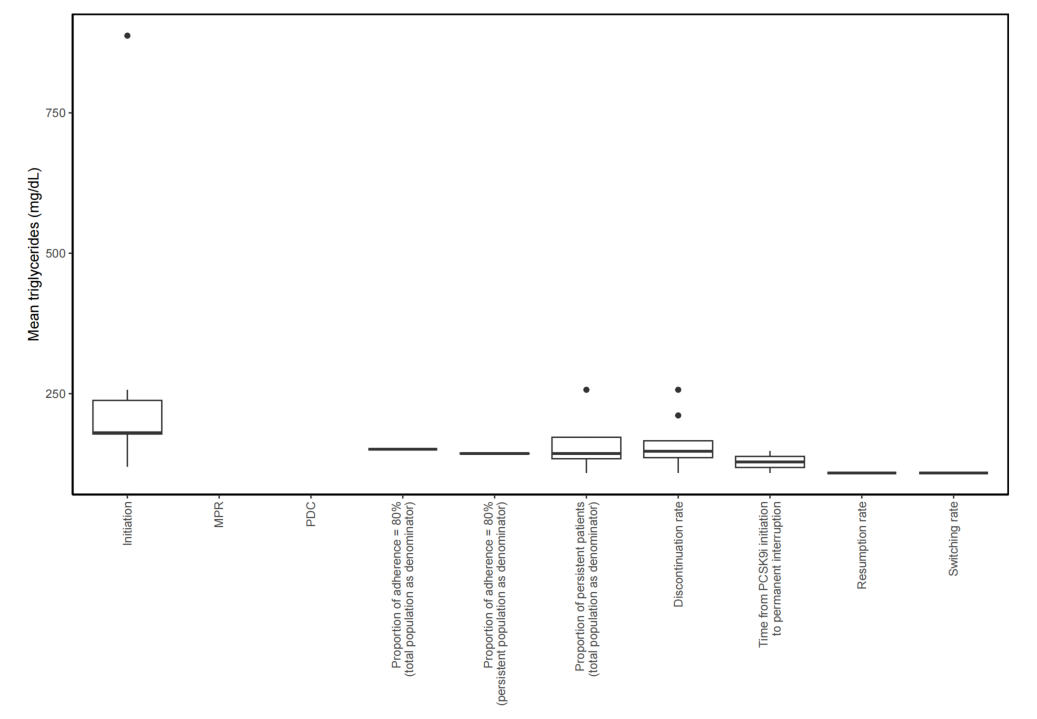


**Figure S5S.** Mean apolipoprotein B [ApoB] (mg/dL)


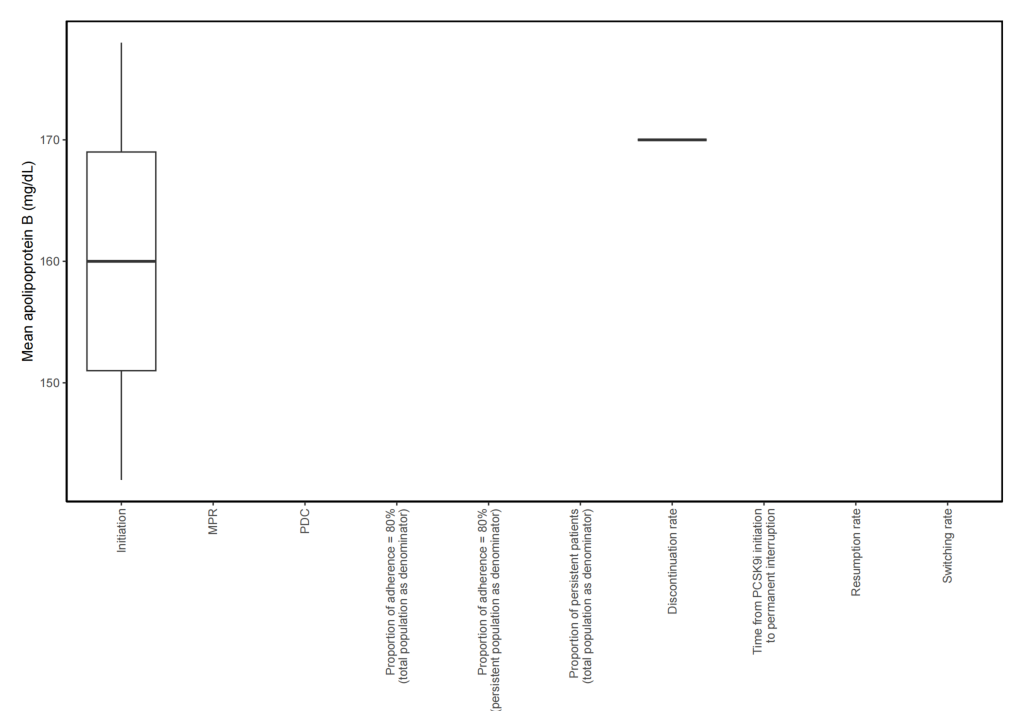


**Figure S5T.** Mean lipoprotein a [Lp(a)] (mg/dL)


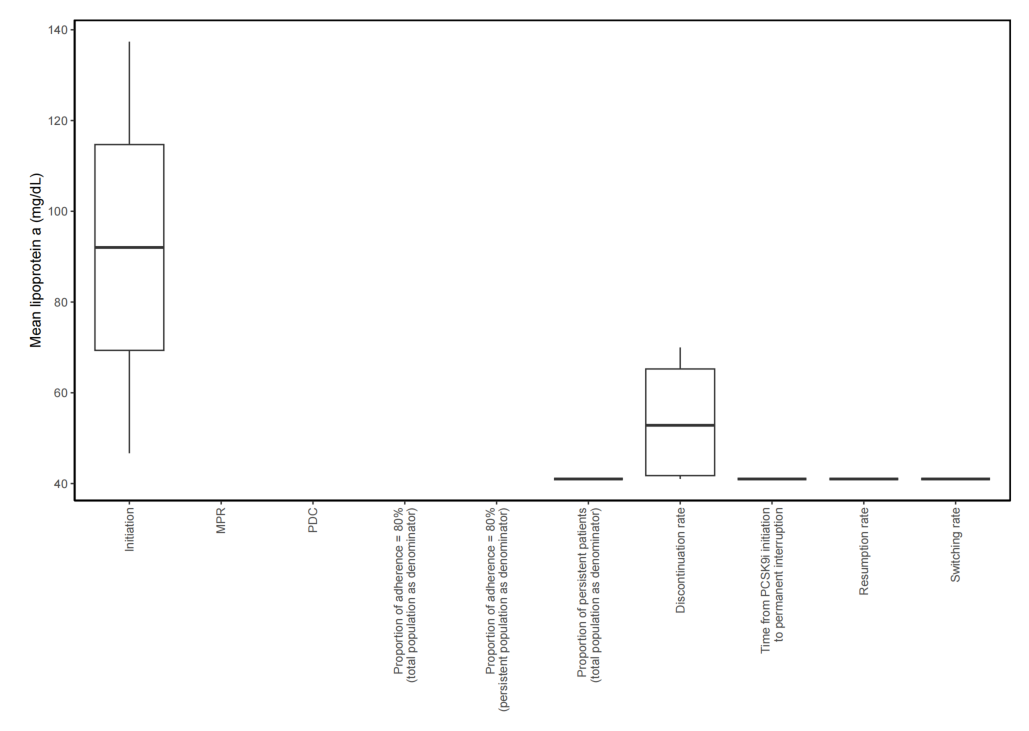


# Summary of results from siRNA PCKS9 inhibitor studies

**Section S6.** A summary of the pooled results from inclisiran studies for primary outcomes

Three studies have investigated the discontinuation rates associated with inclisiran therapy. Mulder 2023 (study #37) reported discontinuation rates of 4.6% (95% CI: 1.0-12.9) at 3 months and 15.4% (95% CI: 7.6-26.5) at the end of the study. Naoum 2024 (study #42) reported discontinuation rates of 6.6% (95% CI: 4.6-9.1) at 3 months and 8.9% (95% CI: 6.6-11.8) at the end of the study. Iqbal 2024 (study #26) reported a discontinuation rate of 3.4% (95% CI: 1.1-7.8) at the end of the study.

The overall discontinuation rate for inclisiran at 3 months and the pooled discontinuation rate was 6.3% (95% CI: 4.6-8.7) with low heterogeneity (I^2^ = 0.0%, P=0.547; **Figure S6A**), and the discontinuation rate for inclisiran for all studies at the end of the study was 8.0% (95%CI: 4.3-14.5, see Figure S6B) with significant heterogeneity (I^2^ = 75.7%, P = 0.016; **Figure S6A**).

**Figure S6A.** Forest plot for discontinuation rate for inclisiran at 3 months


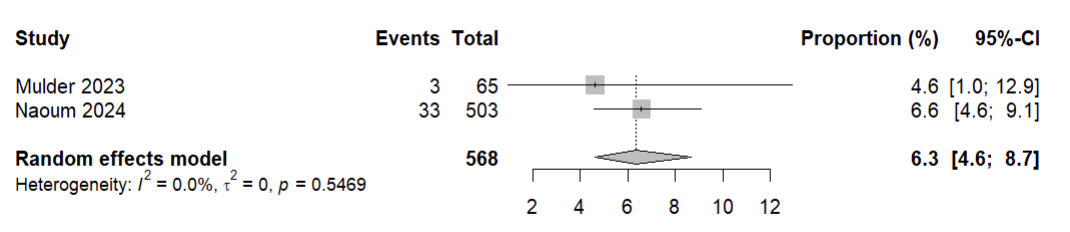


**Figure S6B.** Forest plot for discontinuation rate for inclisiran of all studies at the end of the study


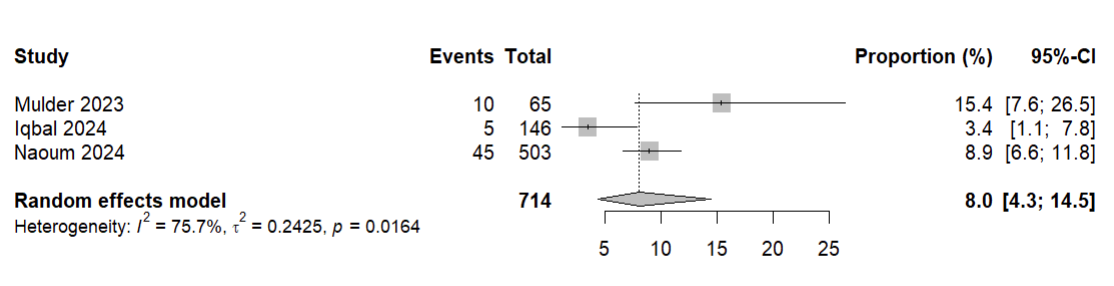


# Quality Assessment

**Figure S7.** Summary of the quality assessment of included studies for main analysis based on the Joanna Briggs Institute (JBI) critical appraisal checklist for prevalence studies


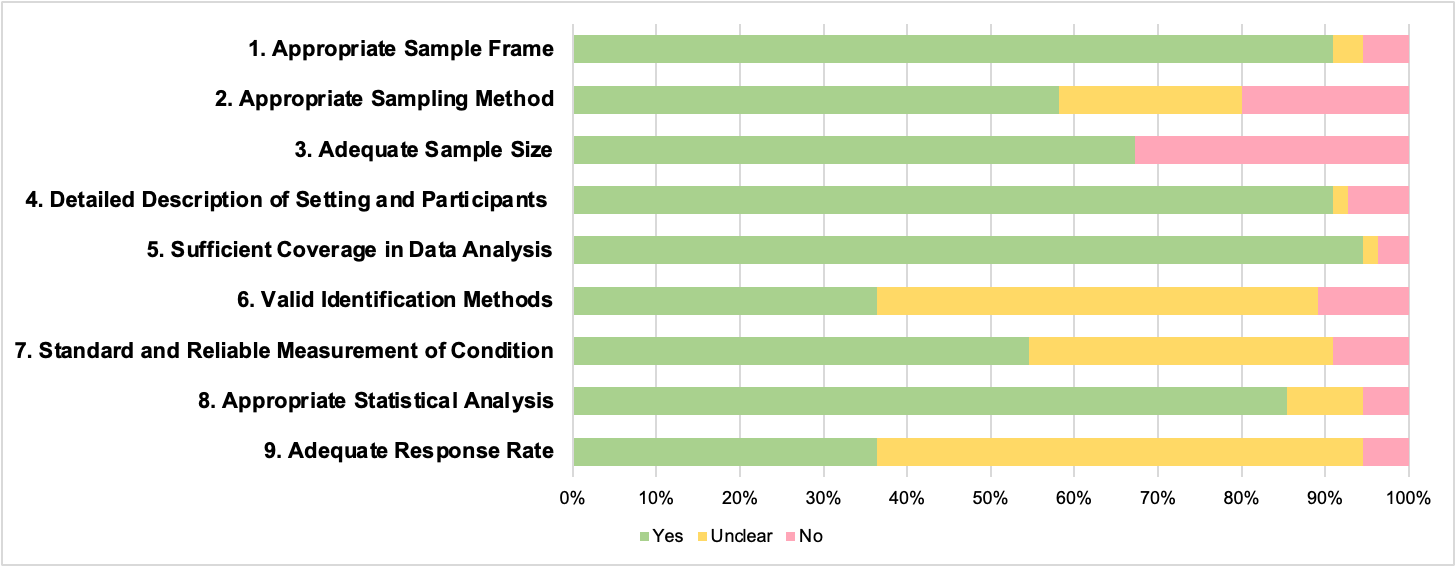


**Table S7.** Quality assessment judgement for each study domain / checklist item of JBI checklist on each study included in the main analysis

| **Study (Lead author, year)** | **1. Appropriate Sample Frame** | **2. Appropriate Sampling Method** | **3.  Adequate Sample Size** | **4.  Detailed Description of Setting and Participants** | **5.  Sufficient Coverage in Data Analysis** | **6.  Valid Identification Methods** | **7.  Standard and Reliable Measurement of Condition** | **8. Appropriate Statistical Analysis** | **9. Adequate Response Rate** | **Total score** |
| --- | --- | --- | --- | --- | --- | --- | --- | --- | --- | --- |
| Alonso 2023 | Yes | Unclear | Yes | Yes | Yes | Unclear | Unclear | Yes | Unclear | 5 |
| Arca 2023 | Yes | No | Yes | Yes | Yes | Yes | Yes | Yes | Unclear | 7 |
| Barrios 2020 | Yes | Yes | Yes | Yes | Yes | Unclear | Unclear | Yes | Unclear | 6 |
| Bosch 2024 | Yes | Yes | Yes | Yes | Yes | Unclear | Yes | Yes | Unclear | 7 |
| Bradley 2019 | Yes | No | Yes | Yes | Yes | No | No | Yes | No | 5 |
| Cannon 2021 | Yes | Yes | Yes | Yes | Yes | Yes | Yes | Yes | Yes | 9 |
| Chai 2023 | Yes | Unclear | No | No | Yes | Unclear | Unclear | Yes | Yes | 4 |
| Chlebus 2022 | Yes | Yes | No | Yes | Unclear | Unclear | Unclear | Yes | Unclear | 4 |
| Chng 2022 | Yes | Unclear | No | Unclear | Yes | Unclear | Yes | Yes | Unclear | 4 |
| Davidson 2020 | Yes | Yes | No | Yes | No | No | No | Yes | No | 4 |
| Davis 2020 | Yes | Unclear | No | Yes | Yes | Unclear | Yes | Yes | Yes | 6 |
| Donald 2022 | Yes | Yes | Yes | Yes | Yes | Yes | No | Yes | Unclear | 7 |
| Eloso 2023 | Yes | No | Yes | Yes | Yes | Yes | Yes | Yes | Yes | 8 |
| Engebretsen 2022 | Yes | Yes | Yes | No | No | Unclear | Yes | Yes | Unclear | 5 |
| Fairman 2017 | Unclear | Yes | Yes | Yes | Yes | Yes | Yes | Yes | Unclear | 7 |
| Fischer 2021 | Yes | No | Yes | Yes | Yes | Unclear | Unclear | No | Yes | 5 |
| Galema-Boers 2023 | Yes | Unclear | Yes | Yes | Yes | No | Unclear | Yes | Unclear | 5 |
| Garcia-Pena 2023 | Yes | Yes | Yes | Yes | Yes | Unclear | Unclear | Yes | Yes | 7 |
| Gargiulo 2024 | Yes | No | Yes | Yes | Yes | Unclear | Unclear | Yes | Unclear | 5 |
| Gayoso-Rey 2021 | Yes | No | Yes | Yes | Yes | Yes | Unclear | Yes | Unclear | 6 |
| Goicoechea 2022 | Yes | Yes | No | Yes | Yes | Unclear | Yes | Yes | Unclear | 6 |
| Gupta 2023 | Yes | Yes | Yes | Yes | Yes | Unclear | Unclear | Unclear | No | 5 |
| Gurgoze 2018 | Yes | Unclear | Yes | Yes | Yes | Unclear | Yes | Yes | Yes | 7 |
| Hines 2018 | Yes | Yes | Yes | Yes | Yes | Yes | Yes | Yes | Yes | 9 |
| Iqbal 2022 | Yes | Unclear | No | Yes | Yes | Unclear | Yes | No | Unclear | 4 |
| Iqbal 2024 | Yes | No | Yes | Yes | Yes | Unclear | Unclear | Yes | Yes | 6 |
| Kaufman 2019 | Yes | Yes | Yes | Yes | Yes | Unclear | Yes | Yes | Unclear | 7 |
| Khatib 2022 | No | No | No | Yes | Yes | Unclear | Yes | Yes | Yes | 5 |
| Kim 2023 | Yes | No | No | Yes | Yes | Unclear | Yes | Yes | Yes | 6 |
| Koenig 2024 | Yes | Yes | Yes | Yes | Yes | Yes | Yes | Yes | Unclear | 8 |
| Kohli 2017 | Yes | Yes | No | Yes | Yes | Unclear | Unclear | Yes | Unclear | 5 |
| Lafratte 2023 | Yes | No | Yes | No | Yes | Yes | Yes | Yes | Unclear | 6 |
| Lahoz 2024 | Yes | Unclear | Yes | Yes | Yes | Yes | Yes | Yes | Unclear | 7 |
| Leitner 2020 | Yes | Unclear | No | Yes | Yes | Unclear | Unclear | Yes | Unclear | 4 |
| Maciejko 2019 | Yes | Yes | No | Yes | Yes | Yes | Yes | Unclear | Yes | 7 |
| Mongiello 2023 | Yes | Yes | Yes | No | Yes | Yes | Yes | Yes | Unclear | 7 |
| Mulder 2023 | Yes | Yes | No | Yes | Yes | Unclear | Unclear | Yes | Unclear | 5 |
| Muntner 2024 | Yes | Yes | Yes | Yes | Yes | Yes | Yes | Yes | Unclear | 8 |
| Nanchen 2022 | No | Yes | No | Yes | Yes | No | No | Yes | Unclear | 4 |
| Naoum 2024 | Yes | Yes | Yes | Yes | Yes | Unclear | Yes | Unclear | Unclear | 6 |
| Oren 2019 | Yes | Yes | No | Yes | Yes | No | Unclear | No | Yes | 5 |
| Parhofer 2019 | Yes | Yes | Yes | Yes | Yes | Unclear | Unclear | Yes | Yes | 7 |
| Piccinni 2019 | Yes | Yes | Yes | Yes | Yes | Yes | Yes | Unclear | Yes | 8 |
| Rallidis 2020 | Yes | Unclear | Yes | Yes | Yes | Unclear | Unclear | Yes | Unclear | 5 |
| Ray 2023 | Yes | Unclear | Yes | Yes | Yes | Unclear | Unclear | Yes | Yes | 6 |
| Reynolds 2019 | Yes | Yes | Yes | Yes | Yes | Yes | Yes | Yes | Yes | 9 |
| Rymer 2020 | Yes | Yes | Yes | Yes | Yes | Yes | Yes | Yes | Unclear | 8 |
| Sheng 2024 | Yes | Yes | Yes | Yes | Yes | Yes | Yes | Yes | Unclear | 8 |
| Stoekenbroek 2017 | No | Yes | Yes | Yes | Yes | No | No | Yes | Unclear | 5 |
| Stummer 2023 | Yes | Yes | Yes | Yes | Yes | Yes | Yes | Yes | Unclear | 8 |
| Svensson 2024 | Yes | Yes | Yes | Yes | Yes | Yes | Yes | Yes | Unclear | 8 |
| Vicente-Valor 2021 | Yes | No | No | Yes | Yes | Unclear | Unclear | Yes | Yes | 5 |
| Warden 2021 | Yes | Yes | No | Yes | Yes | Unclear | Yes | Unclear | Yes | 6 |
| Zafrir 2018 | Yes | Yes | No | Yes | Yes | Unclear | Unclear | Yes | Yes | 6 |
| Zafrir 2020 | Unclear | Unclear | Yes | Yes | Yes | Yes | Yes | Yes | Unclear | 6 |

# Forest plots (random effects) of adherence outcomes

***Initiation phase:***

**Figure S8A.** Forest plot for proportion initiating


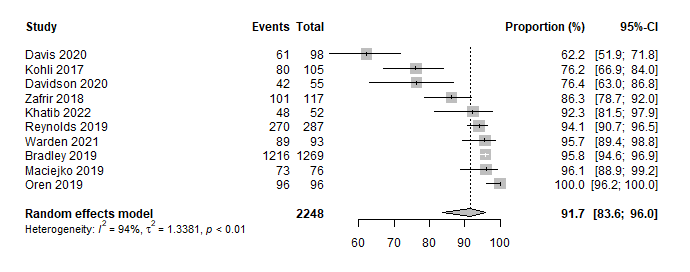


***Implementation phase:***

**Figure S8Bi.** Forest plot for MPR at 6 months (untransformed)


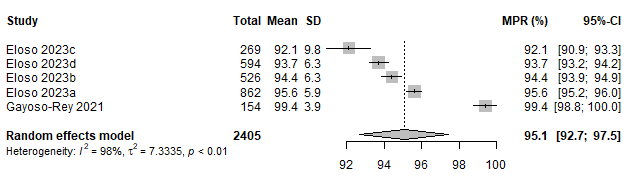


**Figure S8Bii.** Forest plot for medication possession ratio (MPR) at 24 months (untransformed)


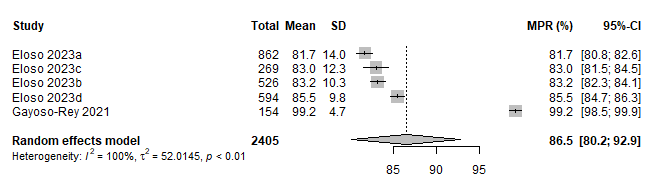


**Figure S8C.** Forest plot for proportion of days covered (PDC) at 12 months (untransformed**)**


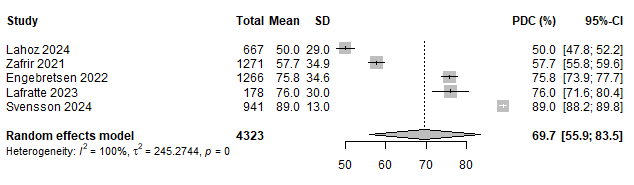


**Figure S8Di.** Forest plot for proportion of adherence ≥80% at 12 months (denominator = total population)


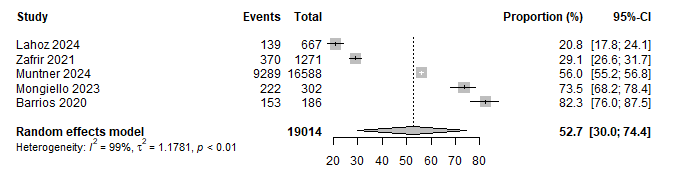


**Figure S8Dii.** Forest plot for proportion of adherence ≥80% at 12 months (denominator = persistent population)


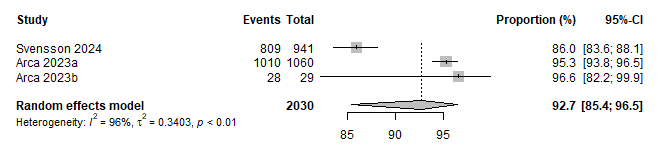


***Persistence phase:***

**Figure S8E.** Forest plot for proportion of persistent patients at 12 months


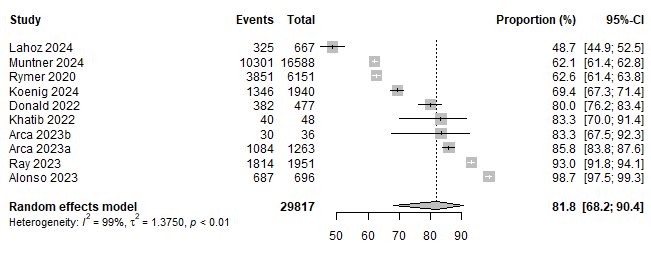


**Figure S8Fi.** Forest plot for discontinuation rate at 12 months


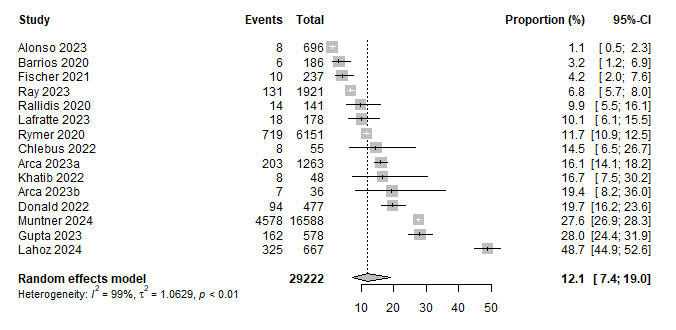


**Figure S8Fii.** Summary forest plot for proportion of discontinuation for different reasons


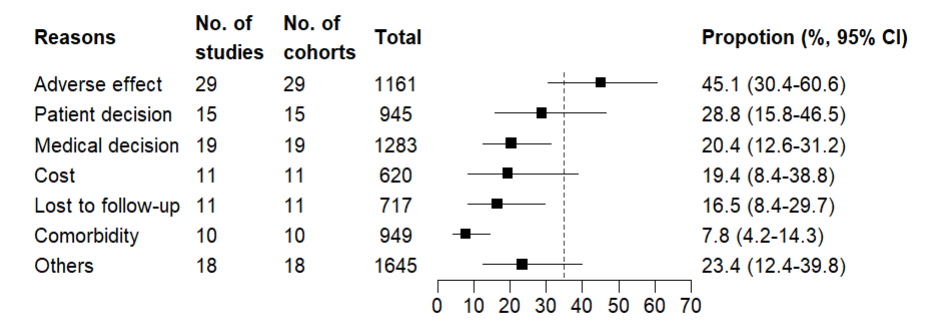


**Figure S8Fiii.** Forest plot for discontinuation due to **(a)** adverse events; **(b)** patient decision; **(c)** medical decision; **(d)** comorbidity; **(e)** cost; **(f)** lost to follow-up; **(g)** other/unknown reasons


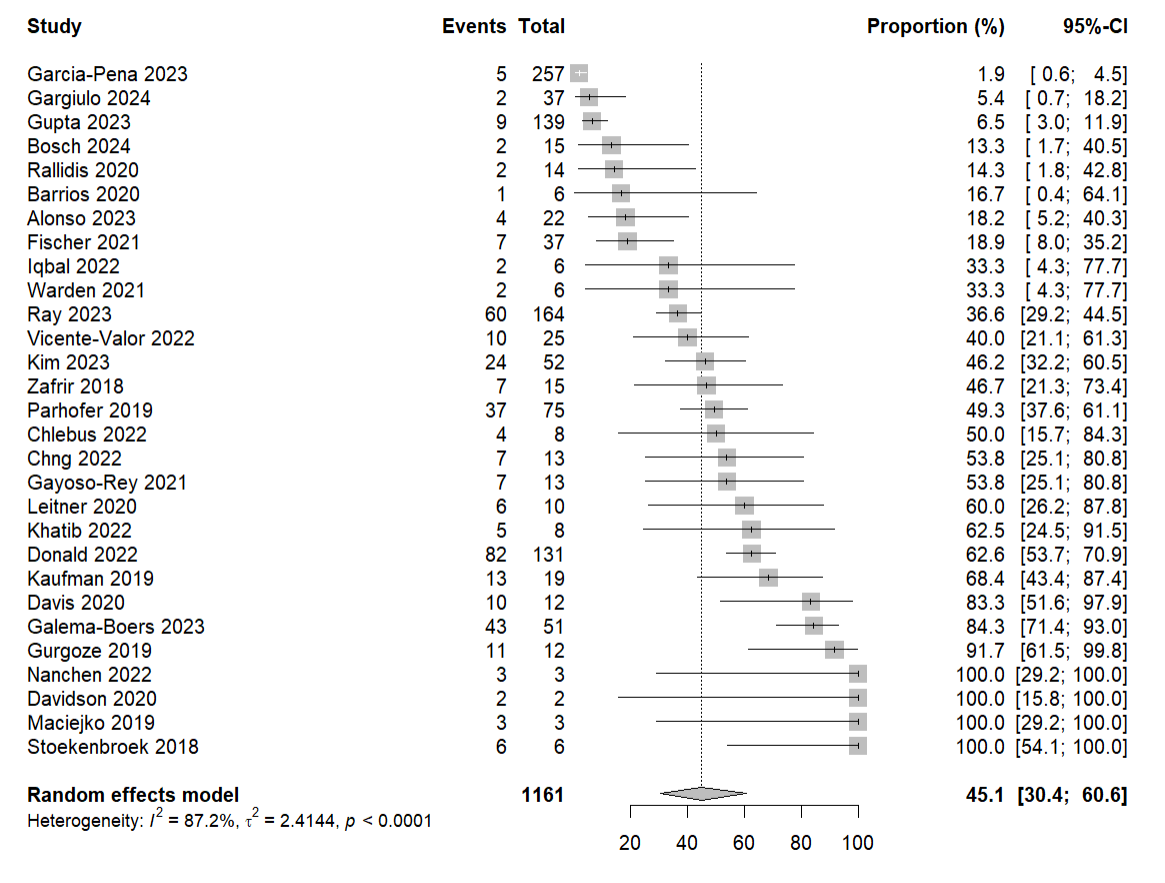


1. adverse events


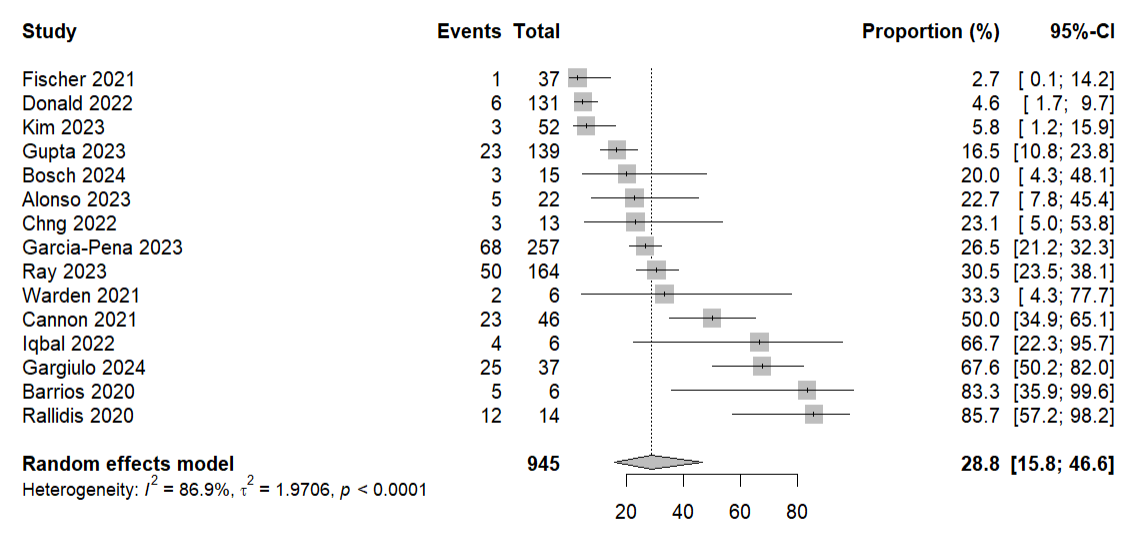


1. patient decision


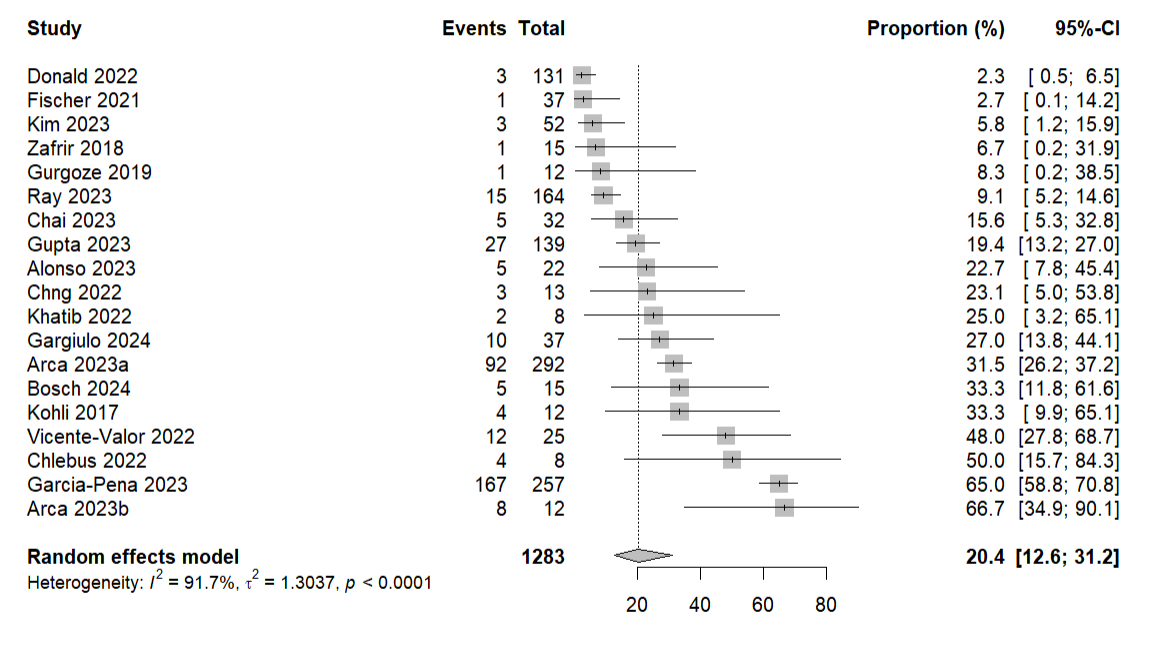


1. medical decision


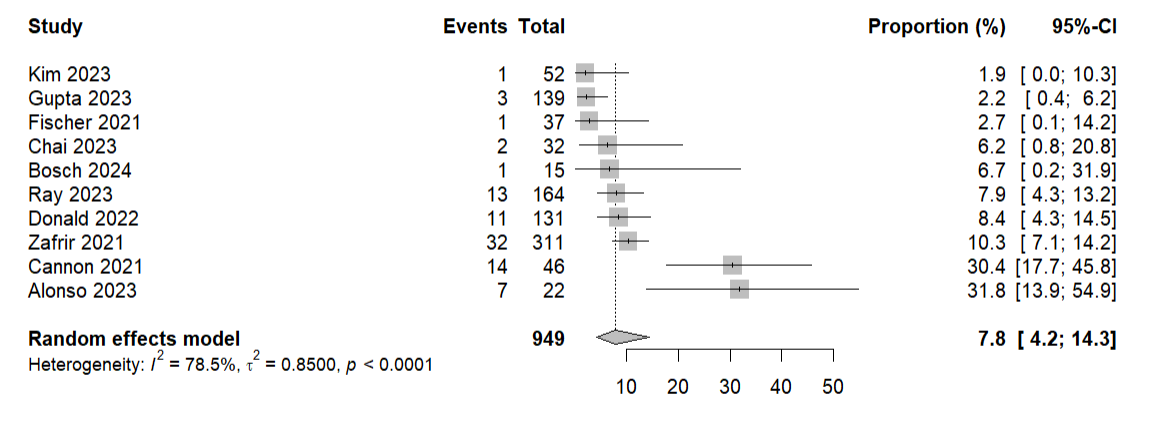


1. comorbidity


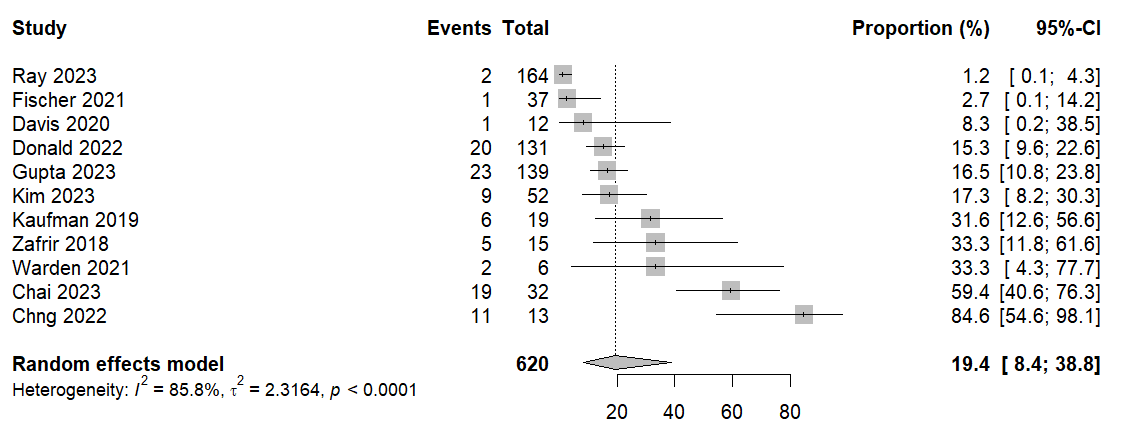


1. cost


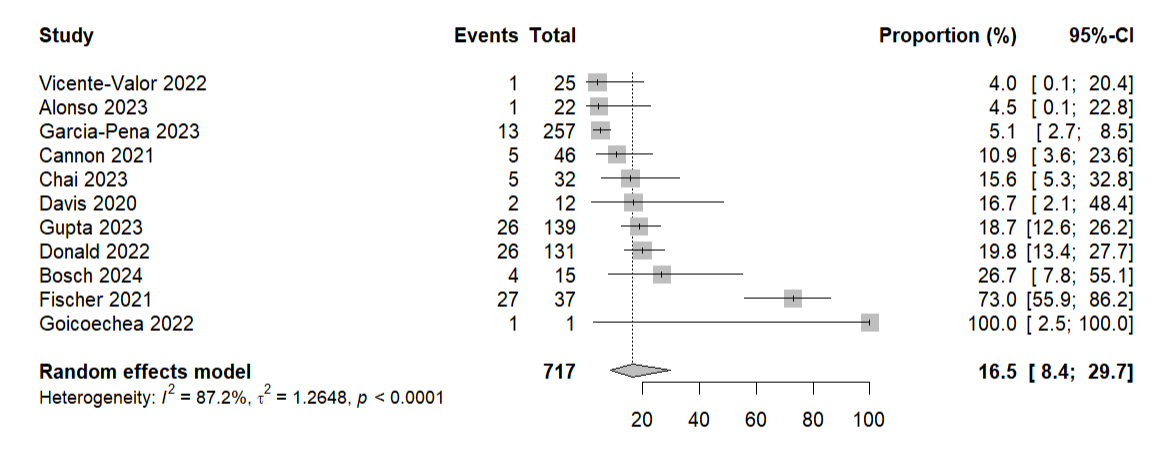


1. lost to follow-up


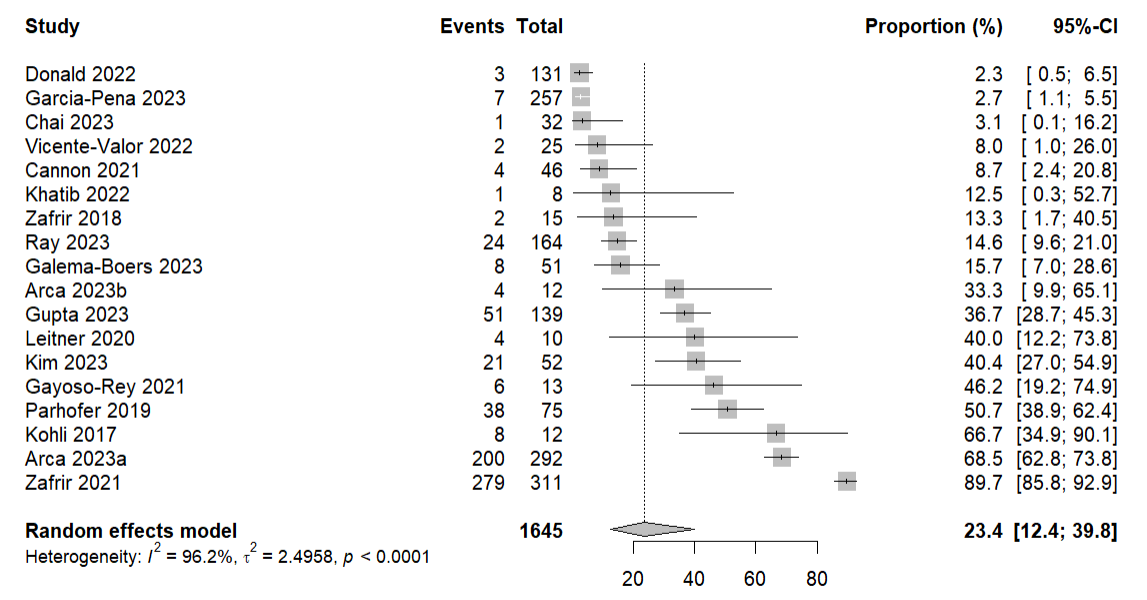


1. other/unknown reasons

**Figure S8G.** Forest plot for time from PCSK9i initiation to permanent discontinuation (log transformed)


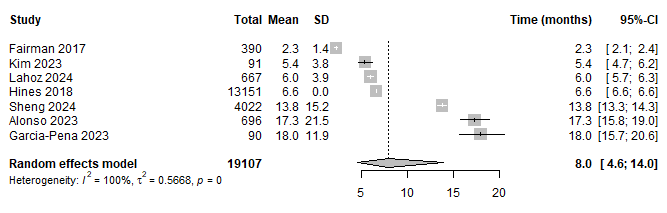


***Reinitiation phase:***

**Figure S8H.** Forest plot for proportion resuming


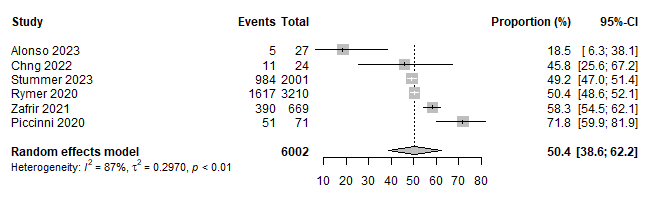


**Figure S8I.** Forest plot for proportion switching


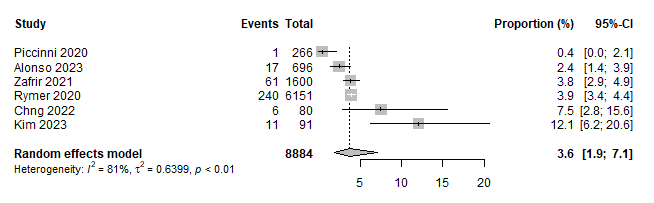


# Subgroup analysis

***Initiation phase:***

**Figure S9Ai.** Subgroup analysis for proportion initiating by region


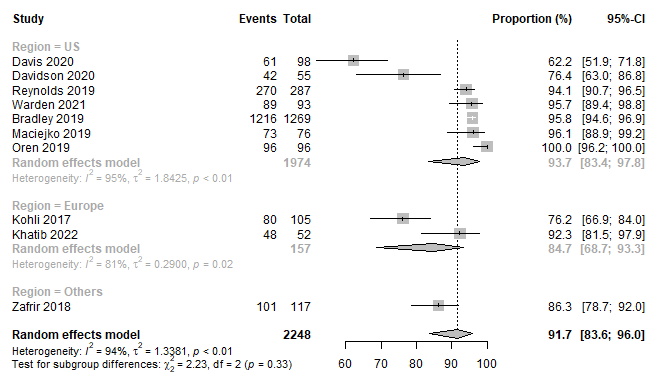


**Figure S9Aii.** Subgroup analysis for proportion initiating by data source


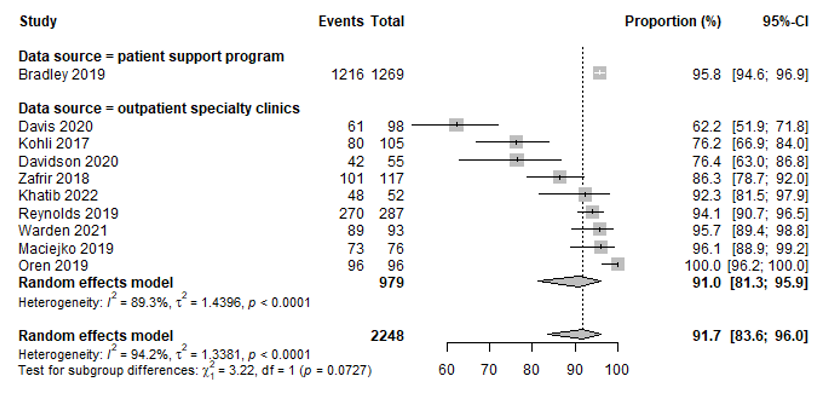


***Implementation phase:***

**Figure S9Bi.** Subgroup analysis for MPR at 6 months by region
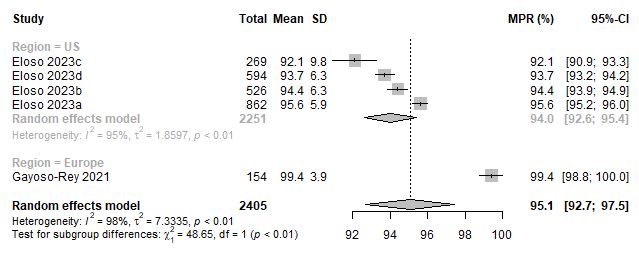


**Figure S9Bii.** Subgroup analysis for MPR at 6 months by data source


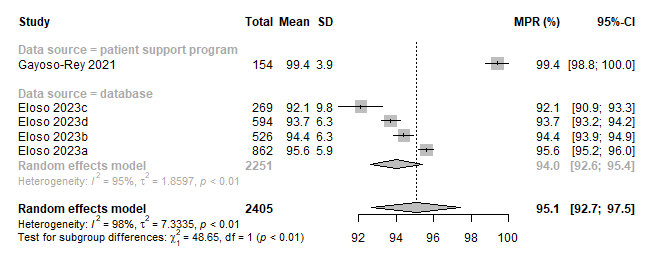


**Figure S9Biii.** Subgroup analysis for MPR at 24 months by region


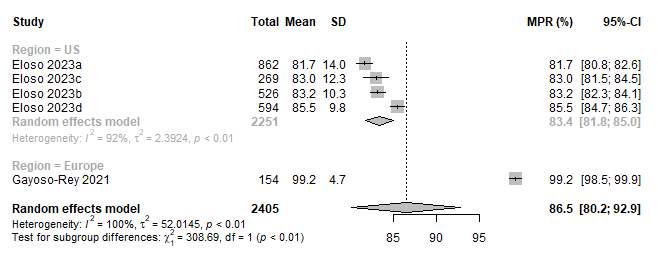


**Figure S9Biv.** Subgroup analysis for MPR at 24 months by data source


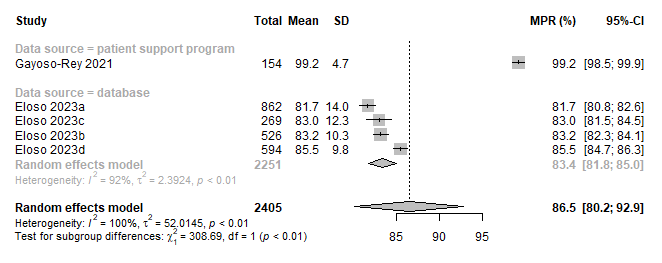


**Figure S9C.** Subgroup analysis for PDC at 12 months by region


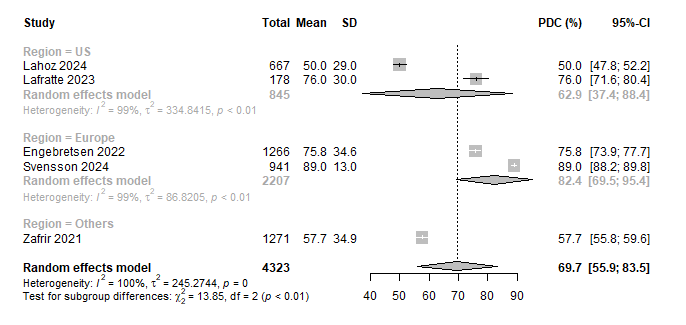


**Figure S9D.** Subgroup analysis for proportion of adherence ≥ 80% at 12 months by region (denominator = total population)


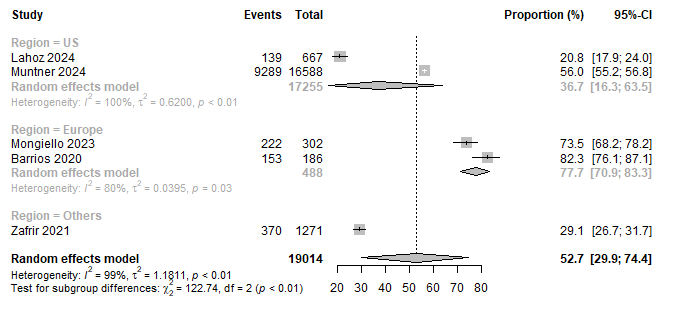


***Persistence phase:***

**Figure S9Ei.** Subgroup analysis for proportion of persistent patients at 12 months by region


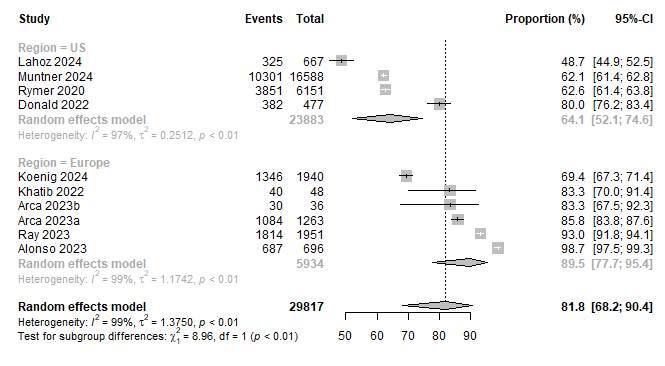


**Figure S9Eii.** Subgroup analysis for proportion of persistent patients at 12 months by data source


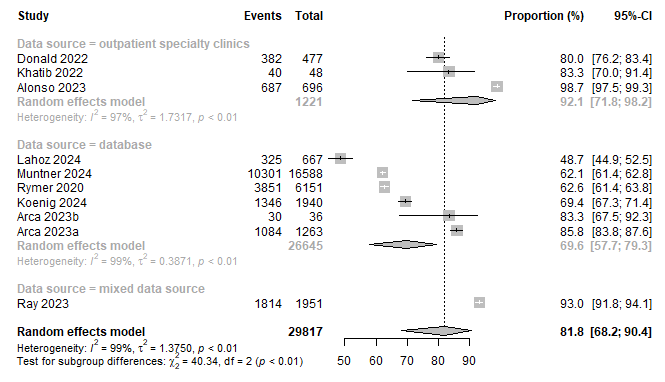


**Figure S9Fi.** Subgroup analysis for discontinuation rate at 12 months by region


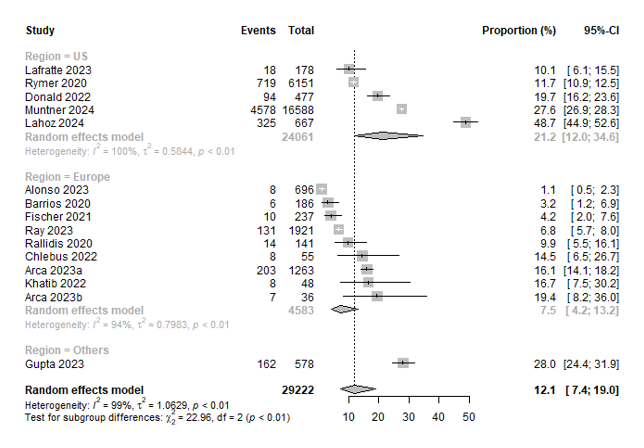


**Figure S9Fii.** Subgroup analysis for discontinuation rate at 12 months by data source


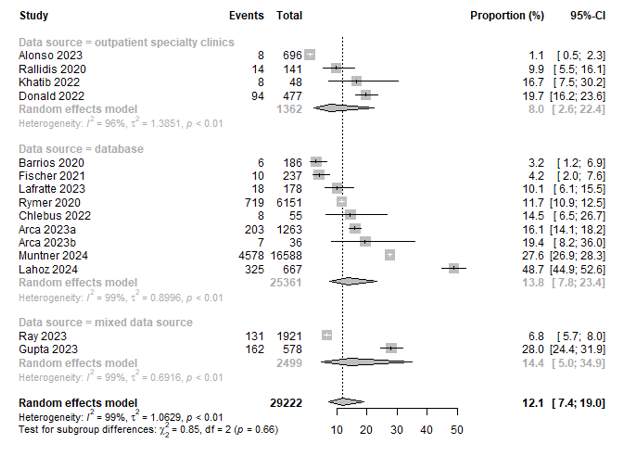


**Figure S9Gi.** Subgroup analysis for time from PCSK9i initiation to permanent discontinuation by region


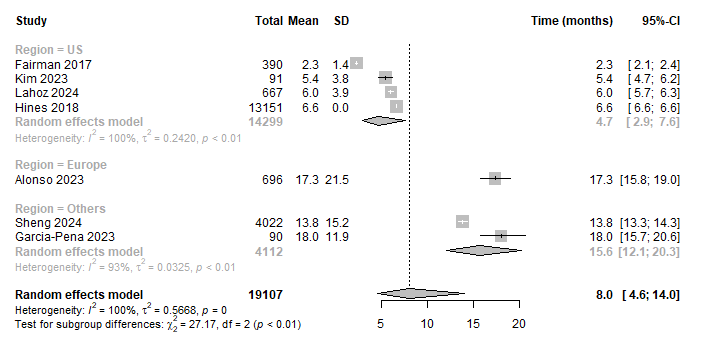


**Figure S9Gii.** Subgroup analysis for time from PCSK9i initiation to permanent discontinuation by data source


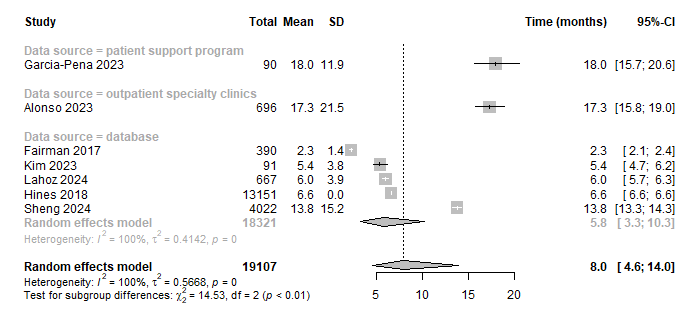


***Reinitiation phase:***

**Figure S9Hi.** Subgroup analysis for resumption rate by region


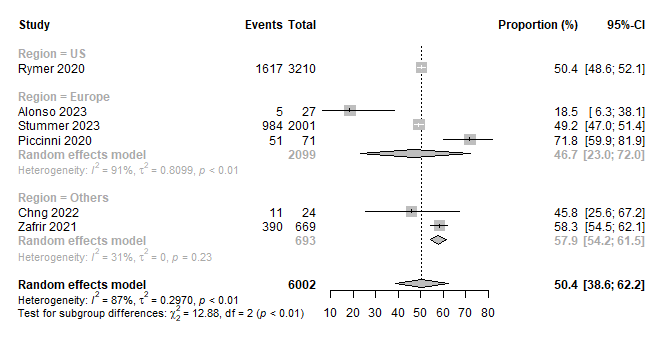


**Figure S9Hii.** Subgroup analysis for resumption rate by data source


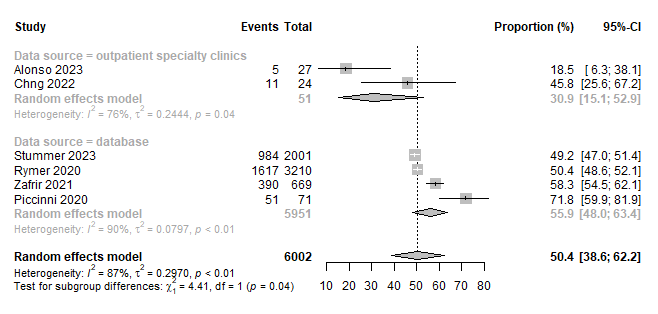


**Figure S9Ii.** Subgroup analysis for switching rate by region


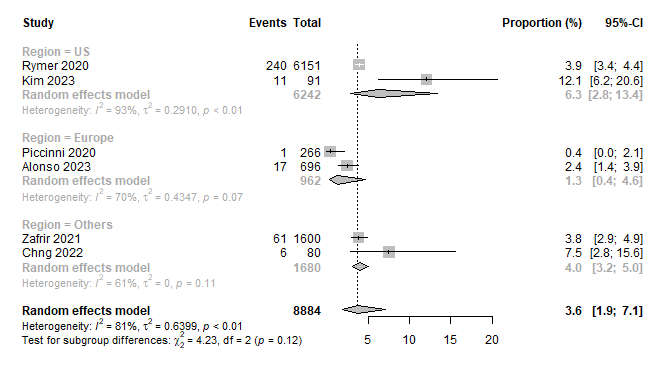


**Figure S9Iii.** Subgroup analysis for switching rate by data source


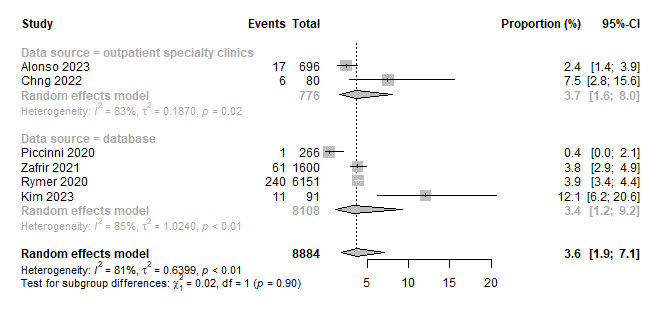


# Meta-regression analysis

**Table S10.** Meta-regression analysis of study-level characteristics and adherence measures

|  | Initiation rate | | | MPR at 6 months | | | MPR at 24 months | | | PDC at 12 months | | | Adherence ≥ 80%  (denominator = total population) | | | Adherence ≥ 80%  (denominator = persistent population) | | |  |
| --- | --- | --- | --- | --- | --- | --- | --- | --- | --- | --- | --- | --- | --- | --- | --- | --- | --- | --- | --- |
|  | **Mean ± SD** | ***β* (95%CI)** | ***P*** | **Mean ± SD** | ***β* (95%CI)** | ***P*** | **Mean ± SD** | ***β* (95%CI)** | ***P*** | **Mean ± SD** | ***β* (95%CI)** | ***P*** | **Mean ± SD** | ***β* (95%CI)** | ***P*** | **Mean ± SD** | ***β* (95%CI)** | ***P*** | |
| Female (%) | 49 ± 6.5 | -8.4  (-19, 2.1) | 0.116 | 14 ± 18 | 0.1  (0.1, 0.2) | <0.001 | 14 ± 18 | 0.4  (0.3, 0.5) | <0.001 | 45 ± 5 | -2.5  (-4.6, -0.4) | 0.023 | 41 ± 11 | -11  (-14, -7.7) | <0.001 | 45 ± 4.8 | 15  (11, 20) | <0.001 | |
| Smoking history (%) | 16 ± 12 | 8.9  (-3.2, 21) | 0.151 | - | - | - | - | - | - | 27 ± 21 | 0  (-1.3, 1.2) | 0.954 | 24 ± 19 | -2.8  (-8.7, 3) | 0.341 | - | - | - | |
| Familial hypercholesterolaemia (%) | 45 ± 19 | -4  (-6.7, -1.4) | 0.003 | - | - | - | - | - | - | - | - | - | - | - | - | 70 ± 52 | 1.3  (1, 1.7) | <0.001 | |
| Atherosclerotic cardiovascular disease (%) | 68 ± 24 | -2  (-5.6, 1.6) | 0.274 | 13 ± 2.4 | -0.5  (-1, -0.1) | 0.02 | 13 ± 2.4 | -0.1  (-1, 0.9) | 0.902 | 66 ± 32 | -0.2  (-0.9, 0.5) | 0.581 | 83 ± 17 | 1.3  (-5.8, 8.5) | 0.718 | 48 ± 30 | -2  (-2.5, -1.4) | <0.001 | |
| Statin intolerance (%) | 75 ± 19 | -2  (-7.6, 3.6) | 0.477 | - | - | - | - | - | - | - | - | - | - | - | - | - | - | - | |
| Diabetes (%) | 21 ± 8.3 | -4.5  (-19, 10) | 0.558 | 37 ± 13 | -0.2  (-0.3, -0.1) | 0.005 | 37 ± 13 | -0.5  (-0.7, -0.3) | <0.001 | 32 ± 9.7 | -2.1  (-2.6, -1.6) | <0.001 | 34 ± 5.4 | -23  (-31, -15) | <0.001 | 9.6 ± 10 | -8.1  (-10, -5.8) | <0.001 | |
| Hypertension (%) | 65 ± 12 | -5.7  (-16, 4.2) | 0.258 | - | - | - | - | - | - | - | - | - | - | - | - | - | - | - | |
| Lipid-lowering therapies (%) | - | - | - | - | - | - | - | - | - | 65 ± 5.2 | 0.2  (-7.5, 7.9) | 0.957 | 74 ± 16 | 5.2  (4.7, 5.6) | <0.001 | - | - | - | |
| Both statin and ezetimibe (%) | 32 ± 27 | 5.4  (3.4, 7.5) | <0.001 | 28 ± 12 | -0.1  (-0.4, 0.1) | 0.211 | 28 ± 12 | -0.1  (-0.8, 0.6) | 0.72 | - | - | - | - | - | - | - | - | - | |
| Statin (%) | 43 ± 23 | 3.8  (0.5, 7.2) | 0.024 | 41 ± 7.9 | -0.3  (-0.5, -0.2) | <0.001 | 41 ± 7.9 | -0.9  (-1.1, -0.7) | <0.001 | 58 ± 40 | -0.1  (-0.9, 0.6) | 0.751 | 56 ± 5.2 | 3.2  (-23, 29) | 0.805 | 48 ± 25 | 3.6  (2.6, 4.6) | <0.001 | |
| Ezetimibe (%) | 42 ± 27 | 4.3  (1, 7.6) | 0.012 | 58 ± 32 | -0.1  (-0.1, 0) | 0.006 | 58 ± 32 | -0.1  (-0.4, 0.1) | 0.177 |  |  |  |  |  |  | 72 ± 48 | 1.4  (1, 1.9) | <0.001 | |
| Age (year) | 64 ± 3.9 | -0.1  (-0.3, 0.1) | 0.318 | 64 ± 3.2 | -0.4  (-1.2, 0.5) | 0.413 | 64 ± 3.2 | -1.5  (-3.4, 0.3) | 0.104 | 65 ± 3.8 | -3.3  (-6.1, -0.6) | 0.017 | 64 ± 5.6 | -0.2  (-0.3, -0.1) | <0.001 | 56 ± 7.6 | -0.2  (-0.2, -0.1) | <0.001 | |
| BMI (kg/m2) | - | - | - | - | - | - | - | - | - | - | - | - | - | - | - | - | - | - | |
| TC (mg/dL) | 270 ± 22 | 0  (0, 0) | 0.99 | - | - | - | - | - | - | - | - | - | - | - | - | - | - | - | |
| LDL-C (mg/dL) | 160 ± 35 | 0  (0, 0) | 0.069 | 140 ± 11 | 0.2  (0.1, 0.4) | <0.001 | 140 ± 11 | 0.5  (0.1, 1) | 0.013 | 140 ± 33 | 0.3  (-0.3, 0.9) | 0.301 | 130 ± 35 | 0  (0, 0.1) | 0.478 | 200 ± 40 | 0  (0, 0) | <0.001 | |
| Non-HDL-C (mg/dL) | - | - | - | - | - | - | - | - | - | - | - | - | - | - | - | - | - | - | |
| HDL-C (mg/dL) | 52 ± 2.6 | 0.1  (-0.2, 0.5) | 0.478 | - | - | - | - | - | - | - | - | - | - | - | - | - | - | - | |
| TG (mg/dL) | 300 ± 290 | 0 (0, 0) | 0.404 | - | - | - | - | - | - | - | - | - | - | - | - | - | - | - | |
| ApoB (mg/dL) | - | - | - | - | - | - | - | - | - | - | - | - | - | - | - | - | - | - | |
| Lp(a) (mg/dL) | - | - | - | - | - | - | - | - | - | - | - | - | - | - | - | - | - | - | |
| Publication year | 2000 ± 1.4 | 0.1  (-0.5, 0.7) | 0.713 | 2000 ± 0.9 | -2.7  (-4.2, -1.2) | <0.001 | 2000 ± 0.9 | -7.9  (-9.7, -6.2) | <0.001 | 2000 ± 1.7 | 2.6  (-7.5, 13) | 0.61 | 2000 ± 2 | -0.2  (-0.7, 0.4) | 0.561 | 2000 ± 0.6 | -1.2  (-1.5, -0.9) | <0.001 | |

**Abbreviations:** ApoB, apolipoprotein B; BMI, body mass index; HDL-C, high-density lipoprotein cholesterol; LDL-C, low-density lipoprotein cholesterol; Lp(a), lipoprotein(a); MPR, medication possession ratio; PDC, proportion of days covered; TC, total cholesterol; TG, triglycerides

**Table S10.** Meta-regression analysis of study-level characteristics and adherence measures (cont.)

|  | Proportion of persistent patients | | | Time from initiation to permanent discontinuation | | | Discontinuation rate | | | | Resumption rate | | | | Switching rate | | |
| --- | --- | --- | --- | --- | --- | --- | --- | --- | --- | --- | --- | --- | --- | --- | --- | --- | --- |
|  | **Mean ± SD** | ***β* (95%CI)** | ***P*** | **Mean ± SD** | ***β* (95%CI)** | ***P*** | **Mean ± SD** | ***β* (95%CI)** | ***P*** | **Mean ± SD** | | ***β* (95%CI)** | ***P*** | **Mean ± SD** | | ***β* (95%CI)** | ***P*** |
| Female (%) | 44 ± 4.5 | -4.4  (-21, 13) | 0.613 | 44 ± 6.7 | 0  (-0.1, 0.1) | 0.744 | 42 ± 6.7 | 5  (-3, 13) | 0.219 | 40 ± 8.6 | | -3  (-8.8, 2.8) | 0.312 | 40 ± 8.6 | | 3  (-6.4, 12) | 0.535 |
| Smoking history (%) | 21 ± 12 | -0.5  (-9.5, 8.6) | 0.922 | - | - | - | 24 ± 19 | 1.9  (-1.5, 5.3) | 0.272 | 26 ± 22 | | 2.9  (1.3, 4.5) | <0.001 | 26 ± 22 | | 0.7  (-0.7, 2) | 0.318 |
| Familial hypercholesterolaemia (%) | 68 ± 36 | 1.2  (-1.1, 3.5) | 0.299 | 61 ± 36 | 0  (0, 0) | 0.677 | 61 ± 36 | -0.3  (-2.1, 1.4) | 0.712 | 52 ± 49 | | -1.2  (-3.9, 1.4) | 0.351 | 59 ± 43 | | -0.3  (-3.5, 2.9) | 0.837 |
| Atherosclerotic cardiovascular disease (%) | 67 ± 27 | -2.6  (-5.3, 0.1) | 0.055 | 65 ± 31 | 0  (0, 0) | 0.151 | 69 ± 26 | 0.7  (-1.4, 2.8) | 0.497 | 67 ± 19 | | 2.7  (-0.9, 6.3) | 0.136 | 71 ± 19 | | 2.6  (-0.2, 5.4) | 0.067 |
| Statin intolerance (%) | 40 ± 21 | 3.9  (3.6, 4.2) | <0.001 |  |  |  | 40 ± 24 | -2.3  (-4, -0.6) | 0.009 | - | | - | - | - | | - | - |
| Diabetes (%) | 22 ± 15 | -6.5  (-11, -2.3) | 0.002 | 23 ± 11 | 0  (-0.1, 0) | 0.603 | 23 ± 13 | 4.1  (-0.1, 8.3) | 0.054 | 28 ± 13 | | 4.1  (-1, 9.1) | 0.112 | 26 ± 12 | | 0.8  (-5.2, 6.8) | 0.791 |
| Hypertension (%) | 63 ± 26 | -5.6  (-8.6, -2.6) | <0.001 | 54 ± 24 | 0  (0, 0) | 0.296 | 62 ± 24 | 3.3  (0.2, 6.4) | 0.037 | 58 ± 23 | | 2.5  (-0.1, 5) | 0.062 | 53 ± 24 | | -0.2  (-2.4, 2) | 0.88 |
| Lipid-lowering therapies (%) | 70 ± 18 | 0.8  (-1.1, 2.6) | 0.424 | 71 ± 13 | 0  (0, 0.1) | <0.001 | 69 ± 20 | 0.7  (-1.3, 2.7) | 0.492 | - | | - | - | - | | - | - |
| Both statin and ezetimibe (%) | 33 ± 25 | 5.5  (3.6, 7.5) | <0.001 | 37 ± 34 | 0  (0, 0) | 0.149 | 46 ± 32 | -1.3  (-3.4, 0.7) | 0.207 | 40 ± 27 | | -2.2  (-5.2, 0.9) | 0.163 | 40 ± 27 | | -0.7  (-1.3, -0.1) | 0.034 |
| Statin (%) | 36 ± 23 | -2  (-5.5, 1.4) | 0.248 | 33 ± 18 | 0  (-0.1, 0) | <0.001 | 39 ± 30 | 1  (-0.6, 2.6) | 0.199 | 36 ± 27 | | 0  (-2.6, 2.7) | 0.98 | 35 ± 24 | | 2  (-0.7, 4.8) | 0.147 |
| Ezetimibe (%) | 42 ± 46 | -0.4  (-2.6, 1.9) | 0.749 | 19 ± 15 | 0  (-0.1, 0) | 0.075 | 36 ± 37 | 0.5  (-1.4, 2.3) | 0.614 | 30 ± 29 | | 0.7  (-1.7, 3.1) | 0.579 | 32 ± 26 | | 1.8  (-0.6, 4.3) | 0.14 |
| Age (year) | 60 ± 6.3 | -0.1  (-0.2, 0) | 0.084 | 65 ± 4.6 | -0.1  (-0.2, 0) | 0.113 | 60 ± 5.2 | 0.1  (-0.1, 0.2) | 0.332 | 62 ± 3.3 | | 0.1  (0.1, 0.2) | <0.001 | 62 ± 3.4 | | 0.1  (-0.1, 0.2) | 0.275 |
| BMI (kg/m2) | - | - | - | - | - | - | 29 ± 2.3 | 0.2  (-0.2, 0.5) | 0.41 | - | | - | - | - | | - | - |
| TC (mg/dL) | 270 ± 43 | 0  (0, 0) | 0.016 | 220 ± 20 | 0  (-0.1, 0) | 0.192 | 250 ± 43 | 0  (0, 0) | 0.011 | - | | - | - | - | | - | - |
| LDL-C (mg/dL) | 160 ± 47 | 0  (0, 0) | 0.648 | 130 ± 26 | 0  (0, 0) | 0.775 | 160 ± 40 | 0  (0, 0) | 0.938 | 150 ± 15 | | 0  (0, 0.1) | <0.001 | 150 ± 13 | | 0  (-0.1, 0.1) | 0.817 |
| Non-HDL-C (mg/dL) | 230 ± 46 | 0  (0, 0) | <0.001 | - | - | - | 230 ± 46 | 0  (0, 0) | <0.001 | - | | - | - | - | | - | - |
| HDL-C (mg/dL) | 54 ± 2.3 | 0.5  (0.2, 0.8) | 0.001 | 52 ± 3.9 | 0.1 (-0.1, 0.3) | 0.353 | 51 ± 3.6 | -0.1  (-0.3, 0.1) | 0.282 | - | | - | - | - | | - | - |
| TG (mg/dL) | 160 ± 65 | 0  (0, 0) | 0.15 |  |  |  | 160 ± 50 | 0  (0, 0) | 0.108 | - | | - | - | - | | - | - |
| ApoB (mg/dL) | - | - | - | - | - | - | - | - | - | - | | - | - | - | | - | - |
| Lp(a) (mg/dL) | - | - | - | - | - | - | 54 ± 15 | 0  (0, 0.1) | 0.25 | - | | - | - | - | | - | - |
| Publication year | 2000 ± 1.2 | 0  (-0.7, 0.6) | 0.944 | 2000 ± 2.9 | 0.2  (0, 0.3) | <0.001 | 2000 ± 1.4 | 0.4  (0, 0.7) | 0.058 | 2000 ± 1.7 | | -0.3  (-0.5, 0) | 0.024 | 2000 ± 1.7 | | 0.3  (-0.1, 0.7) | 0.104 |

**Abbreviations:** ApoB, apolipoprotein B; BMI, body mass index; HDL-C, high-density lipoprotein cholesterol; LDL-C, low-density lipoprotein cholesterol; Lp(a), lipoprotein(a); MPR, medication possession ratio; PDC, proportion of days covered; TC, total cholesterol; TG, triglycerides

# Additional results on adherence outcomes

**Table S11A.** Meta-analyses of other time points

|  | **Monoclonal antibodies** | | | | ***I*^2^** |
| --- | --- | --- | --- | --- | --- |
|  | **No. of studies** | **No. of cohorts** | **Sample size** | **Pooled results (95% CI)** |  |
| **MPR, %** | | | | | |
| 12 months | 1 | 4 | 2251 | 87.7 (86.1-89.3) | 92.9% |
| 24+ months | 1 | 4 | 2251 | 79.7 (78.9-80.5) | 58.7% |
| **PDC, %** | | | | | |
| 6 months | 3 | 3 | 17775 | 79.7 (63.1-96.3) | 100.0% |
| **Proportion of adherence ≥80% (total population as denominator), %** | | | | | |
| 6 months | 2 | 2 | 29739 | 55.7 (46.3-64.6) | 99.8% |
| **Proportion of adherence ≥80% (persistent population as denominator), %** | | | | | |
| 6 months | 2 | 3 | 2596 | 90.9 (89.2-92.4) | 63.8% |
| 18 months | 1 | 2 | 1059 | 96.0 (94.7-97.1) | 0.0% |
| 24 months | 2 | 3 | 1395 | 95.0 (79.5-98.9) | 97.5% |
| **Proportion of persistent patients, %** | | | | | |
| 3 months | 3 | 3 | 6689 | 86.9 (75.8-93.4) | 96.6% |
| 6 months | 6 | 7 | 25163 | 88.2 (70.8-95.9) | 96.8% |
| 18 months | 1 | 2 | 1299 | 82.8 (80.6-84.7) | 34.8% |
| 24 months | 4 | 5 | 4270 | 76.7 (64.9-85.4) | 98.4% |
| **Discontinuation rate, %** | | | | | |
| 3 months | 5 | 5 | 890 | 5.9 (2.7-12.3) | 80.5% |
| 6 months | 9 | 10 | 32216 | 17.1 (10.0-27.7) | 99.5% |
| 18 months | 1 | 2 | 1299 | 18.5 (16.5-20.7) | 3.2% |
| 24 months | 5 | 6 | 3180 | 13.0 (5.7-26.9) | 97.4% |

**Abbreviations:** MPR, medication possession ratio; PDC, proportion of days covered

**Table S11B.** Summary of the between-study, within-study and sampling error variance for moderation analyses in the three-level random effects models

|  | **MPR (%)** | **Proportion of adherence ≥80% (%)** | **Proportion of persistent patients (%)** | **Discontinuation rate (%)** |
| --- | --- | --- | --- | --- |
| **Between-study variance *I*^2^** | 39.4 | 70.2 | 47.3 | 92.7 |
| **Within-study variance *I*^2^** | 60.3 | 26.2 | 52.3 | 7.0 |
| **Sampling error variance** | 0.3 | 3.6 | 0.4 | 0.3 |

**Abbreviations:** MPR, medication possession ratio

# Sensitivity analysis

**Table S12.** Sensitivity analyses for meta-analyses at specific time points. N.B. pooled estimates in bold indicate identical results to the main analysis, as all included studies had a JBI score ≥6 and were judged to be at low risk of bias.

|  | **Sensitivity analysis 1*** | | | **Sensitivity analysis 2*** | | |
| --- | --- | --- | --- | --- | --- | --- |
|  | **No. of studies** | **No. of cohorts** | **Pooled estimate (95% CI)** | **No. of studies** | **No. of cohorts** | **Pooled estimate (95% CI)** |
| **Initiation rate, %** | 5 | 5 | 90.5 (78.9-96.1) | 10 | 10 | 90.0 (81.9-95.9) |
| **MPR at 6 months, %** | **2** | **5** | **95.1 (92.7-97.5)** | 2 | 5 | 95.0 (92.7-97.4) |
| **MPR at 24 months, %** | **2** | **5** | **86.5 (80.2-92.9)** | 2 | 5 | 86.3 (80.5-92.6) |
| **PDC at 12 months, %** | 4 | 4 | 68.2 (50.8-85.5) | 5 | 5 | 68.2 (55.6-83.8) |
| **Proportion of adherence ≥ 80% (total population as denominator) at 12 months, %** | **5** | **5** | **52.7 (30.0-74.4)** | 5 | 5 | 52.4 (28.2-76.0) |
| **Proportion of adherence ≥ 80% (persistent population as denominator) at 12 months, %** | **2** | **3** | **92.7 (85.4-96.5)** | 2 | 3 | 92.6 (84.9-97.8) |
| **Proportion of persistent patients at 12 months, %** | 7 | 8 | 75.7 (63.8-84.6) | 9 | 10 | 78.7 (67.3-88.2) |
| **Discontinuation rate at 12 months, %** | 8 | 9 | 15.0 (8.9-24.2) | 14 | 15 | 14.1 (8.5-20.8) |
| **Time from PCSK9i initiation to permanent discontinuation, months** | 6 | 6 | 7.0 (3.9-12.7) | NA | NA | NA |
| **Resumption rate, %** | 4 | 4 | 55.9 (48.0-63.4) | 6 | 6 | 50.2 (37.2-63.1) |
| **Switching rate, %** | 4 | 4 | 3.4 (1.2-9.2) | 6 | 6 | 3.9 (1.5-7.3) |

**Abbreviations:** MPR, medication possession ratio; NA, not applicable; PDC, proportion of days covered.
*****Sensitivity analysis 1 includes only studies with a JBI score ≥6 (low risk of bias); Sensitivity analysis 2 was double arcsine transformation for proportions or log transformation for continuous variables, except for the time from PCSK9i initiation to permanent interruption

***Initiation phase:***

**Figure S12Ai.** Sensitivity analysis for proportion initiating (JBI checklist total score ≥ 6)


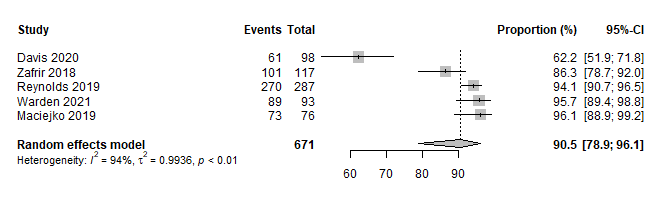


**Figure S12Aii.** Sensitivity analysis for initiation rate (Freeman-Tukey double arcsine transformation)


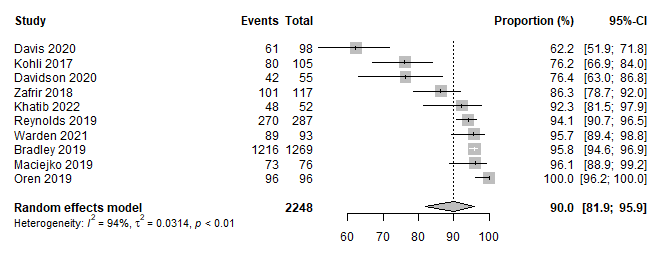


***Implementation phase:***

**Figure S12Bi.** Sensitivity analysis for MPR at 6 months (JBI checklist total score ≥ 6)


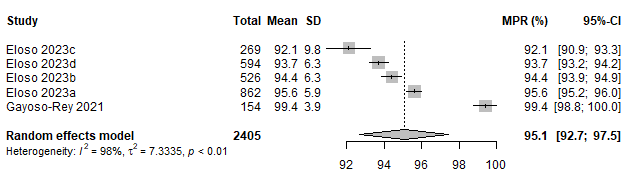


**Figure S12Bii.** Sensitivity analysis for MPR at 6 months (log transformation)


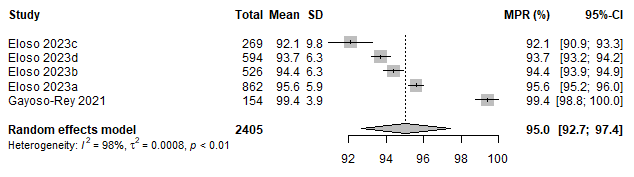


**Figure S12Biii.** Sensitivity analysis for MPR at 24 months (JBI checklist total score ≥ 6)


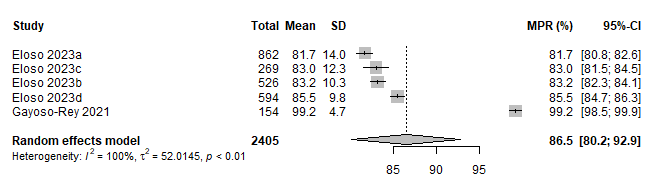


**Figure S12Biv.** Sensitivity analysis for MPR at 24 months (log transformation)


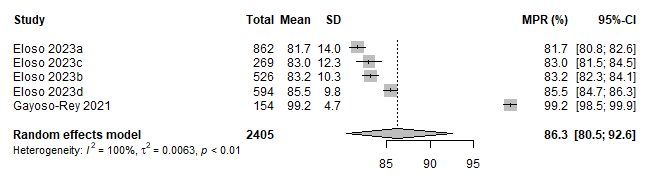


**Figure S12Ci.** Sensitivity analysis for PDC at 12 months (JBI checklist total score ≥ 6)


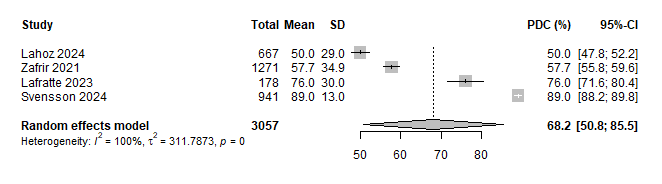


**Figure S12Cii.** Sensitivity analysis for PDC at 12 months (log transformation)


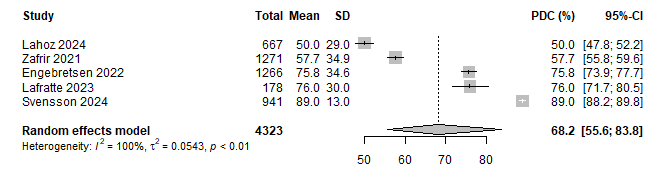


**Figure S12Di.** Sensitivity analysis for proportion of adherence ≥ 80% at 12 months (denominator = total population, JBI checklist total score ≥ 6)


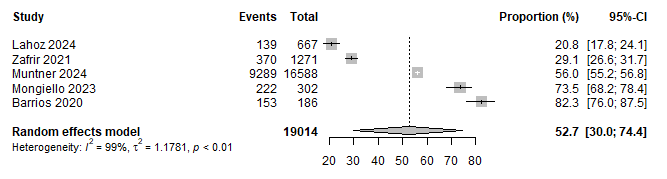


**Figure S12Dii.** Sensitivity analysis for proportion of adherence ≥ 80% at 12 months (denominator = total population, Freeman-Tukey double arcsine transformation)


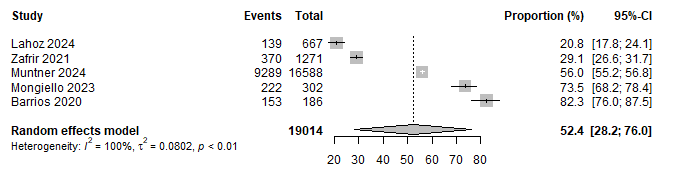


**Figure S12Diii.** Sensitivity analysis for proportion of adherence ≥ 80% at 12 months (denominator = persistent population, JBI checklist total score ≥ 6)


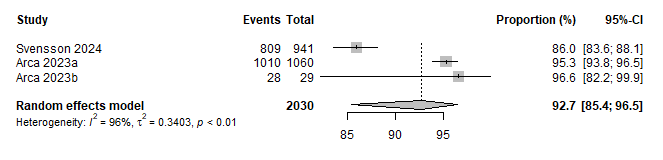


**Figure S12Div.** Sensitivity analysis for proportion of adherence ≥ 80% at 12 months (denominator = persistent population, Freeman-Tukey double arcsine transformation)


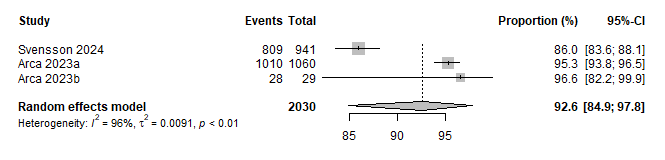


**Figure S12Ei.** Sensitivity analysis for proportion of persistent patients at 12 months (JBI checklist total score ≥ 6)


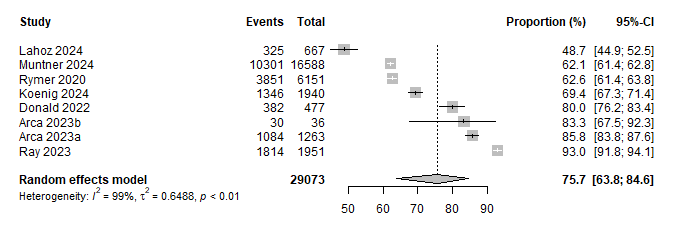


**Figure S12Eii.** Sensitivity analysis for proportion of persistent patients at 12 months (Freeman-Tukey double arcsine transformation)


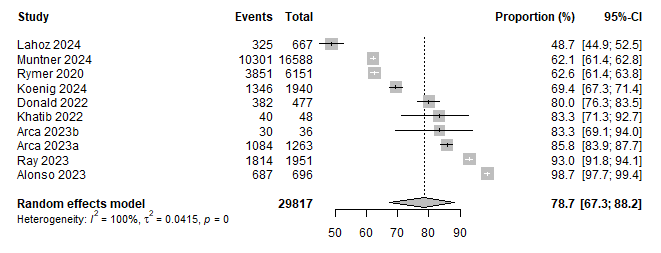


**Figure S12Fi.** Sensitivity analysis for discontinuation rate at 12 months (JBI checklist total score ≥ 6)


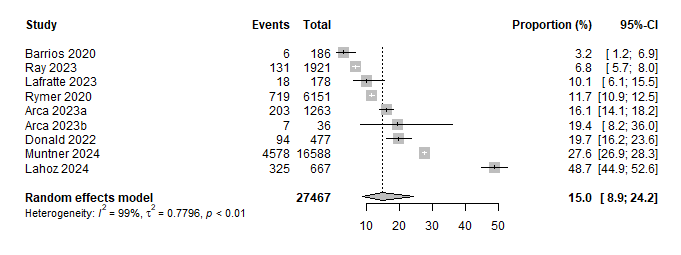


**Figure S12Fii.** Sensitivity analysis for discontinuation rate at 12 months (Freeman-Tukey double arcsine transformation)


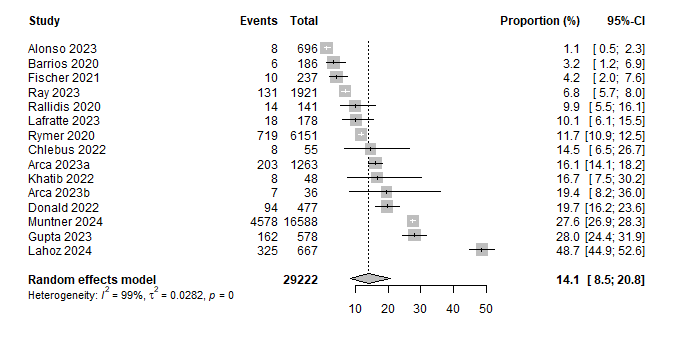


**Figure S12G.** Sensitivity analysis for time from PCSK9i initiation to permanent discontinuation (JBI checklist total score ≥ 6)


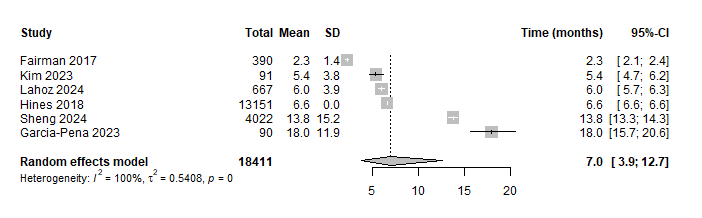


**Figure S12Hi.** Sensitivity analysis for resumption rate (JBI checklist total score ≥ 6)


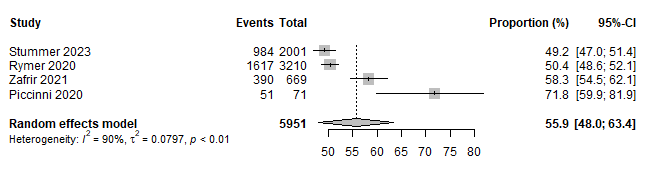


**Figure S12Hii.** Sensitivity analysis for resumption rate (Freeman-Tukey double arcsine transformation)


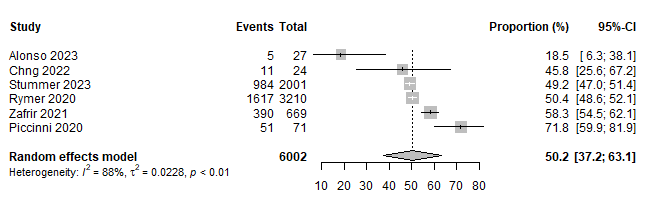


**Figure S12Ii.** Sensitivity analysis for switching rate (JBI checklist total score ≥ 6)


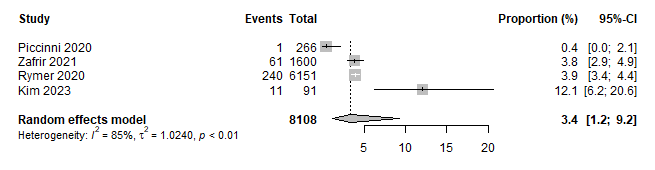


**Figure S12Iii.** Sensitivity analysis for switching rate (Freeman-Tukey double arcsine transformation)


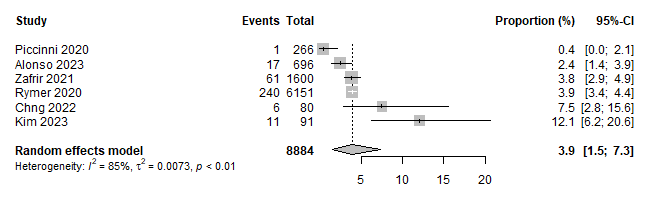


# Assessment of publication bias

**Table S13.** Egger’s tests for meta-analyses at specific time points

| **Outcome** | **Egger’s test**  **(p-value)** |
| --- | --- |
|  |  |
| **Initiation rate, %** | 0.983 |
| **MPR at 6 months, %** | 0.835 |
| **MPR at 24 months, %** | 0.386 |
| **PDC at 12 months, %** | 0.161 |
| **Proportion of adherence ≥ 80% (total population as denominator) at 12 months, %** | 0.601 |
| **Proportion of adherence ≥ 80% (persistent population as denominator) at 12 months, %** | 0.601 |
| **Proportion of persistent patients at 12 months, %** | 0.050 |
| **Discontinuation rate at 12 months, %** | 0.104 |
| **Time from PCSK9i initiation to permanent discontinuation, months** | 0.614 |
| **Resumption rate, %** | 0.791 |
| **Switching rate, %** | 0.909 |

**Abbreviations:** MPR, medication possession ratio; PDC, proportion of days covered.

**Figure S13A.** Funnel plot for proportion initiating

**
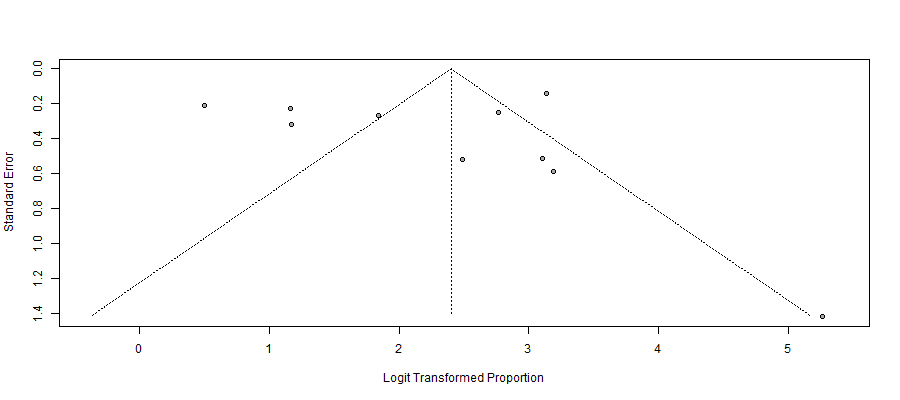
**

**Figure S13B.** Funnel plot for proportion of persistent patients at 12 months

**
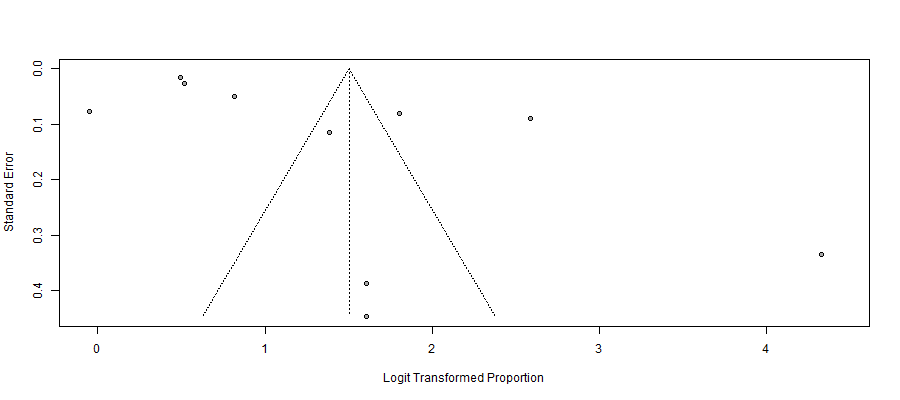
**

**Figure S13Ci.** Funnel plot for discontinuation rate at 12 months

**
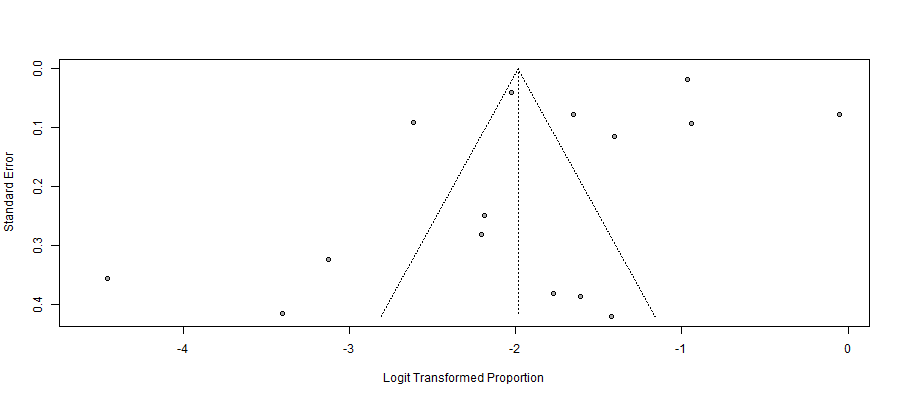
**

**Figure S13Cii.** Funnel plot for discontinuation due to **(a)** adverse events; **(b)** patient decision; **(c)** medical decision; **(d)** comorbidity; **(e)** cost; **(f)** lost to follow-up; **(g)** other/unknown reasons


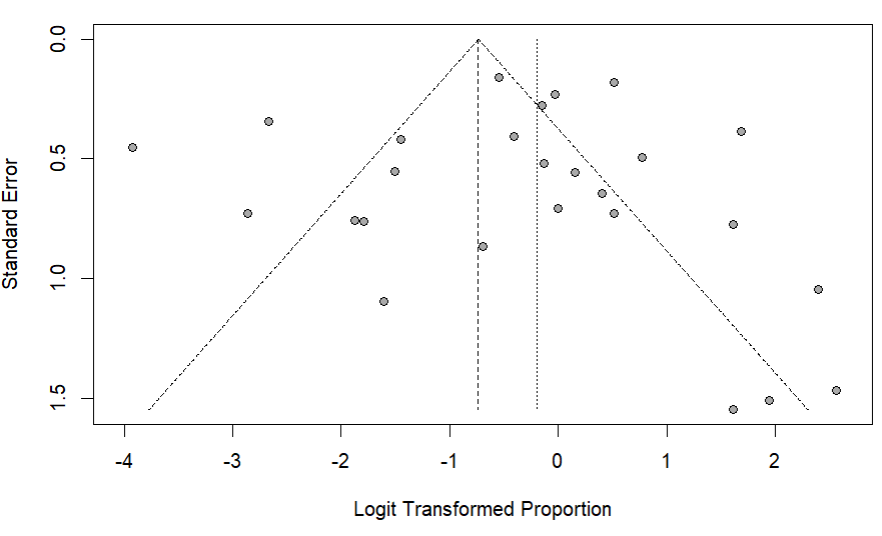


- - 1. adverse events


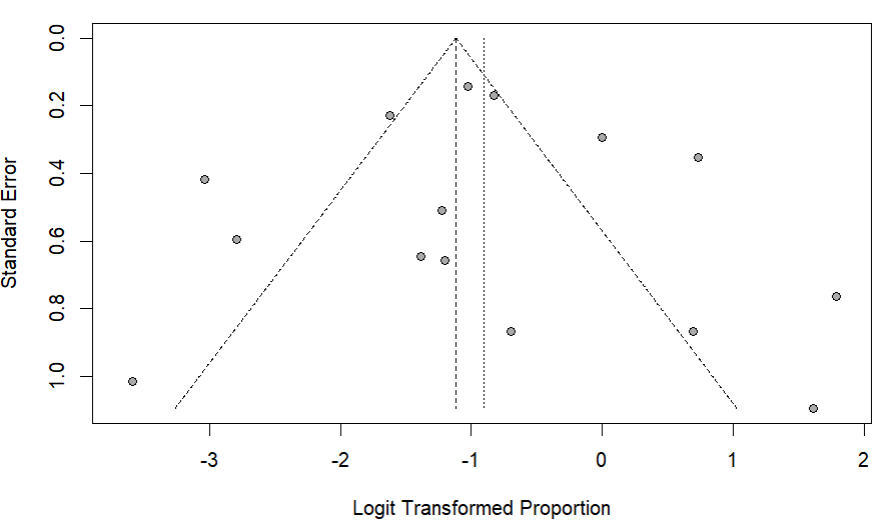


- - 1. patient decision


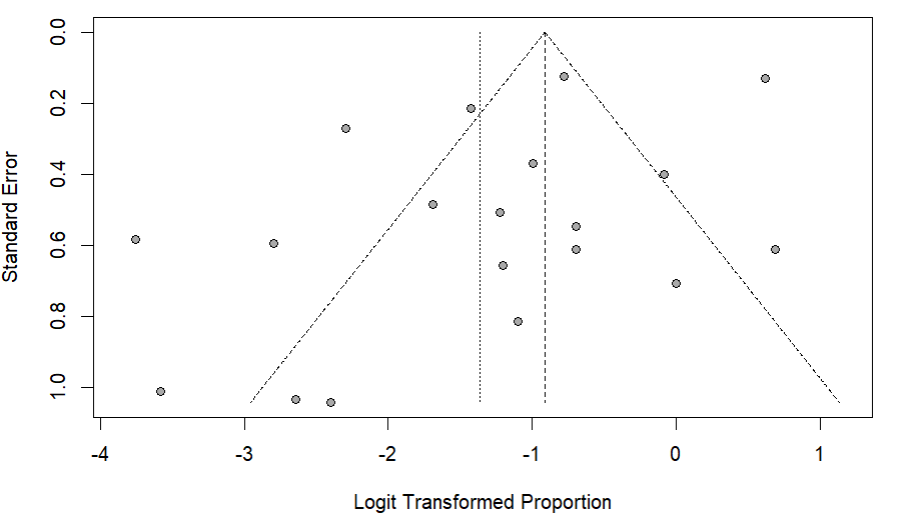


- - 1. medical decision


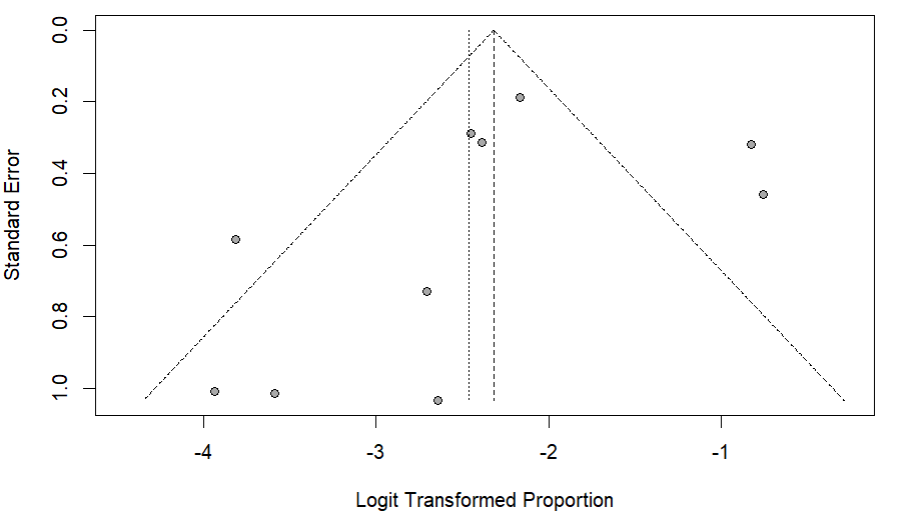


- - 1. comorbidity


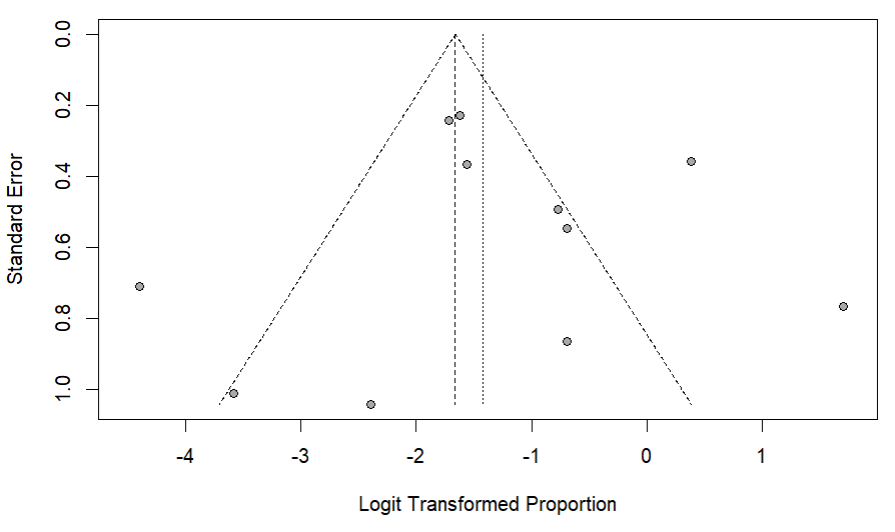


- - 1. cost


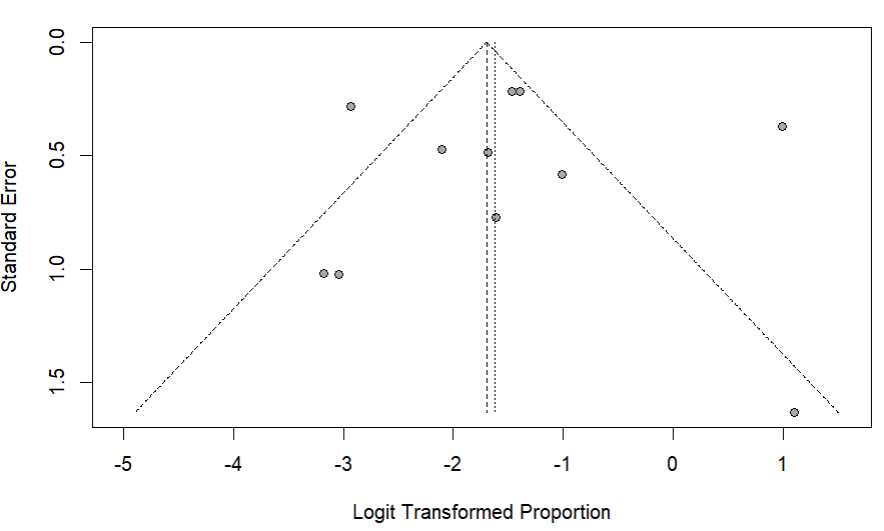


- - 1. lost to follow-up


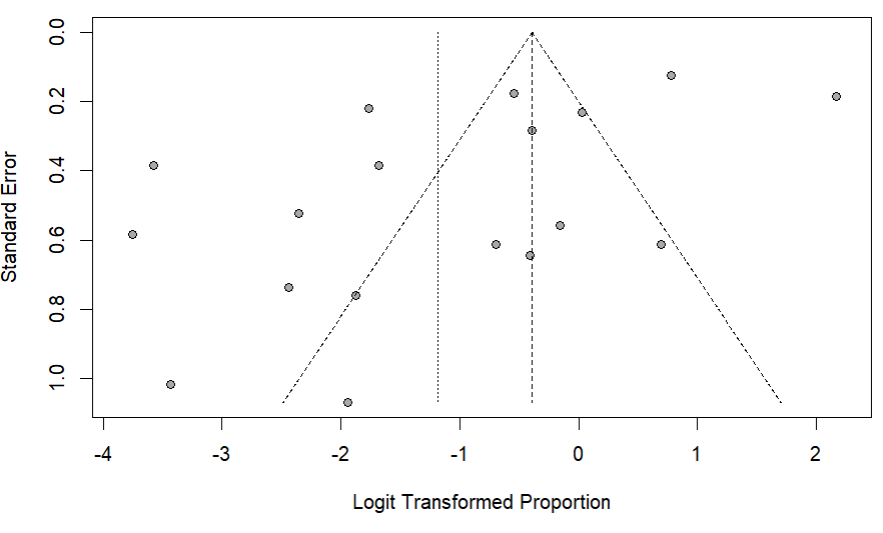


- - 1. other/unknown reasons

# Reference list of all included studies

1. **Ali 2024**

Ali A, Shaat M, Popescue F, Ali-Qureshi S. Lipid lowering injectable clinic-novel idea for improving compliance with twice yearly dosing of inclisiran. Journal of Clinical Lipidology. 2024;18(4 Supplement):e495.

1. **Alonso 2023**
2. Alonso R, Arroyo-Olivares R, Muniz-Grijalvo O, Diaz-Diaz JL, Munoz-Torrero JS, Romero MJ, et al. Persistence with long-term PCSK9 inhibitor treatment and its effectiveness in familial hypercholesterolaemia: data from the SAFEHEART study. European Journal of Preventive Cardiology. 2023;30(4):320-8.
3. Alonso R, Arroyo-Olivares R, Muniz-Grijalvo O, Diaz-Diaz JL, Sanchez Munoz-Torrero J, Zambon D, et al. Persistence to long-term PCSK9 inhibitors treatment and its effectiveness in familial hypercholesterolemia: Data from the SAFEHEART study. European Respiratory Journal. 2022;60(Supplement 66):2372.
4. **Arca 2023**

Arca M, Celant S, Olimpieri PP, Colatrella A, Tomassini L, D'Erasmo L, et al. Real-world effectiveness of PCSK9 inhibitors in reducing LDL-C in patients with familial hypercholesterolemia in Italy: A retrospective cohort study based on the AIFA monitoring registries. Journal of the American Heart Association. 2023;12(21):e026550.

1. **Bajaj 2018**

Bajaj H, Garg V, Pandey A, Verma S. PCSK 9 inhibition: Real world application and challenges to achieve unmet cholesterol targets in canadian atherosclerotic heart disease, the practical study. Journal of the American College of Cardiology. 2018;71(11 Supplement 1).

1. **Barrios 2020**
2. Barrios V, Escobar C, Arrarte V, Garcia E, Fernandez MR, Rincon LM, et al. First national registry on the effectiveness and safety of evolocumab in clinical practice in patients attended in cardiology in Spain. The RETOSS-CARDIO study. Clinica e Investigacion en Arteriosclerosis. 2020;32(6):231-41.
3. Barrios V, Antorrena Miranda MI, Bonanad Lozano C, Cosin Sales J, Diaz De Castro O, Fernandez Del Olmo MR, et al. Clinical profile of patients initiating evolocumab in Spanish Cardiology Units: A RETrospective, Observational Study of Real-World Clinical Practice (RETOSS-Cardio). European Heart Journal. 2019;40(Supplement 1):980.
4. **Bartsch 2024**

Bartsch K, Davidson E, Bagnola A, Mehta L, Larry J, Milks W, et al. Real world experience with inclisiran in an academic medical center. Journal of Clinical Lipidology. 2024;18(4 Supplement):e551-e2.

1. **Bosch 2024**

Bosch M, Danes I, Ballarin E, Marrero P, Vancells G, Ortiz-Zuniga A, et al. Effectiveness and safety of alirocumab and evolocumab for hypercholesterolemia in a population with high cardiovascular risk. Medicina Clinica. 2024((Bosch, Danes, Ballarin, Filippi-Arriaga, Agusti) Clinical Pharmacology Department, Vall d'Hebron Hospital Universitari, Vall d'Hebron Barcelona Hospital Campus, Barcelona, Spain(Bosch, Danes, Agusti) Department of Pharmacology, Therapeutics and Toxicolog).

1. **Bradley 2019**
2. Bradley CK, Shrader P, Sanchez RJ, Peterson ED, Navar AM. The patient journey with proprotein convertase subtilisin/kexin type 9 inhibitors in community practice. Journal of Clinical Lipidology. 2019;13(5):725-34.
3. Navar AM, Sanchez RJ, Peterson ED. PCSK9 inhibitors: Patient-reported barriers to medication initiation and persistence. Circulation. 2017;136(Supplement 1).
4. **Buckley 2019**

Buckley A, O'Connor C, Cahill C, Khullar N, Arockiam S, Ahern C, et al. Effect of PCSK9 inhibitors on clinical outcomes and patient empowerment: A single-centre experience. Heart. 2019;105(Supplement 7):A26-A7.

1. **Cadenas Chamorro 2018**

Cadenas Chamorro R, Paredes B, Garcia Yubero C, Cano R, Casanova C, Prieto E, et al. Anti-PCSK9 in everyday clinical practice. European Journal of Preventive Cardiology. 2018;25(2 Supplement 1):S36-S7.

1. **Cancela Diez 2018**

Cancela Diez B, Munoz C, Claramunt R, Guijarro S, Barbero MJ, Horno F. Efficacy and safety of evolocumab in hypercholesterolaemia and mixed dyslipidaemia. European Journal of Hospital Pharmacy. 2018;25(Supplement 1):A176.

1. **Cannon 2021**
2. Cannon CP, De Lemos JA, Rosenson RS, Ballantyne CM, Liu Y, Gao Q, et al. Use of lipid-lowering therapies over 2 years in GOULD, a registry of patients with atherosclerotic cardiovascular disease in the US. JAMA Cardiology. 2021;6(9):1060-8.
3. Shaik A, Kosiborod M, de Lemos JA, Gao Q, Mues KE, Alam S, et al. Use of lipid-lowering therapies in patients with chronic kidney disease and atherosclerotic cardiovascular disease: 2-year results from getting to an improved understanding of low-density lipoprotein cholesterol and dyslipidemia management (GOULD). Clinical Cardiology. 2022;45(12):1303-10.
4. **Caso 2022**

Caso VM, Sperlongano S, Liccardo B, Romeo E, Padula S, Arenga F, et al. The impact of the COVID-19 outbreak on patients' adherence to PCSK9 inhibitors therapy. Journal of Clinical Medicine. 2022;11(3):475.

1. **Chai 2023**

Chai M, Zhang H, Yang L, Liang J, Han H, Liu X, et al. Lipid lowering effects and safety of evolocumab in Chinese patients at very high cardiovascular risk: A single-center study. Chinese Medical Journal. 2023;136(11):1358-60.

1. **Chlebus 2022**

Chlebus K, Cybulska B, Dobrowolski P, Romanowska-Kocejko M, Zarczynska-Buchowiecka M, Gilis-Malinowska N, et al. Effectiveness and safety of PCSK9 inhibitor therapy in patients with familial hypercholesterolemia within a therapeutic program in Poland: Preliminary multicenter data. Cardiology Journal. 2022;29(1):62-71.

1. **Chng 2022**

Chng BLK, Heng WMP, Soon YM, Hon JS, Lau YH, Tan RS, et al. Safety, adherence and efficacy of PCSK9 inhibitors: A retrospective real-world study. Proceedings of Singapore Healthcare. 2022;31.

1. **Chugh 2019**

Chugh AR, Dobariya V, Austin C, Kioussopoulos K, Rao V, Kovacich D. Real-world utilization and discontinuation patterns of PCSK9 inhibitor use at a large, community-based lipid clinic. Journal of the American College of Cardiology. 2019;73(9 Supplement 1):1781.

1. **Claramunt Garcia 2020**

Claramunt Garcia R, Munoz Cid CL, Sanchez Ruiz A, Lopez AM, Perez Cano E, Jimenez Lopez Y, et al. Use, efficacy and adherence to treatment with PCKS9 inhibitors in real clinical practice. European Journal of Hospital Pharmacy. 2020;27(SUPPL 1):A56.

1. **Dalla Valle 2020**

Dalla Valle C, Paolini C, Lobascio I, Tenaglia R, Cavedon S, Dolci G, et al. Side effects of treatment with PCSK9i, expected or unexpected? European Heart Journal, Supplement. 2020;22(SUPPL G):G34.

1. **Davidson 2020**

Davidson ER, Snider MJ, Bartsch K, Hirsch A, Li J, Larry J. Tolerance of proprotein convertase subtilisin/kexin type 9 (PCSK9) inhibitors in patients with self-reported statin intolerance. Journal of Pharmacy Practice. 2020;33(3):276-82.

1. **Davis 2020**

Davis LE, Pogge EK. A retrospective chart review evaluating efficacy, tolerability, and cost of proprotein convertase subtilisin/kexin type 9 inhibitors (PCSK9i) in older adults. High Blood Pressure and Cardiovascular Prevention. 2020;27(4):331-8.

1. **Desai 2023**

Desai NR, Van Hise N, Niu C, Ghera E, McElligott S. The adherence and LDL-C lowering effect of inclisiran among patients who received treatment at outpatient clinics. Circulation. 2023;148(Supplement 1).

1. **Dominguez Bachiller 2020**

Dominguez Bachiller M, Barcia Martin MI, Gomez Pedrero AM, Perez Encinas M. Alirocumab and evolocumab: Results in clinical practice. European Journal of Hospital Pharmacy. 2020;27(SUPPL 1):A56-A7.

1. **Donald 2022**

Donald DR, Reynolds VW, Hall N, DeClercq J, Choi L. Exploring rates of PCSK9 inhibitor persistence and reasons for treatment non-persistence in an integrated specialty pharmacy model. Journal of Clinical Lipidology. 2022;16(3):315-24.

1. **Elis 2023**

Elis A, Melzer Cohen C, Chodick G. Real-world use of alirocumab: Experience from a large healthcare provider. Journal of Clinical Medicine. 2023;12(3):1084.

1. **Eloso 2023**

Eloso J, Awad A, Zhao X, Cunningham FE, Zhang R, Dong D, et al. PCSK9 inhibitor use and outcomes using concomitant lipid-lowering therapies in the Veterans Health Administration. American Journal of Medicine Open. 2023;9((Eloso, Cunningham, Zhang, Dong, Aspinall) VA Center for Medication Safety/Pharmacy Benefits Management Services, Hines, Ill, United States(Awad) Jesse Brown VA Medical Center, Chicago, Ill, United States(Zhao, Aspinall) VA Center for Health Equity Resear):100035.

1. **Engebretsen 2022**

Engebretsen I, Munkhaugen J, Bugge C, Halvorsen S, Odegaard KM, Stovring H, et al. Gaps and discontinuation of statin treatment in Norway: Potential for optimizing management of lipid lowering drugs. European heart journal open. 2022;2(6):oeac070.

1. **Fairman 2017**
2. Fairman KA, Davis LE, Sclar DA. Real-world use of PCSK-9 inhibitors by early adopters: Cardiovascular risk factors, statin co-treatment, and short-term adherence in routine clinical practice. Therapeutics and Clinical Risk Management. 2017;13((Fairman, Davis, Sclar) Department of Pharmacy Practice, College of Pharmacy, Midwestern University-Glendale, Glendale, AZ, United States):957-65.
3. Fairman KA, Davis LE, Sclar DA. Real-world use of PCSK-9 inhibitors by early adopters: Cardiovascular risk factors, statin co-treatment, and short-term adherence in routine clinical practice. Pharmacotherapy. 2017;37(12):e141.
4. **Fischer 2021**

Fischer LT, Hochfellner DA, Knoll L, Pottler T, Mader JK, Aberer F. Real-world data on metabolic effects of PCSK9 inhibitors in a tertiary care center in patients with and without diabetes mellitus. Cardiovascular Diabetology. 2021;20(1):89.

1. **Galema-Boers 2023**
2. Galema-Boers AMH, Mulder JWCM, Steward K, Roeters van Lennep JE. Sex differences in efficacy and safety of PCSK9 monoclonal antibodies: A real-world registry. Atherosclerosis. 2023;384((Galema-Boers, Mulder, Steward, Roeters van Lennep) Department of Internal Medicine, Erasmus University Medical Center, Rotterdam, Netherlands):117108.
3. Galema-Boers AMH, Lenzen MJ, Sijbrands EJ, Roeters van Lennep JE. Proprotein convertase subtilisin/kexin 9 inhibition in patients with familial hypercholesterolemia: Initial clinical experience. Journal of Clinical Lipidology. 2017;11(3):674-81.
4. Galema-Boers AMH, Steward K, Mulder JWCM, Roeters Van Lennep JE. Sex differences in efficacy and side effects of proprotein convertase subtilisin / kexin 9 (PCSK9) inhibitors in real world data. Atherosclerosis. 2022;355((Galema-Boers, Steward, Mulder, Roeters Van Lennep) Internal Medicine, Erasmus MC, Rotterdam, Netherlands(Steward) Department Of Internal Medicine, Erasmus Mc, University Medical Centre Rotterdam, Rotterdam, Netherlands):155.
5. Galema-Boers JMH, Steward K, Engelkes SR, Sijbrands EJ, Roeters Van Lennep JE. Real world experience of efficacy and safety using proprotein convertase subtilisin/kexin 9 (PCSK9) inhibition in patients with familial hypercholesterolemia. European Journal of Cardiovascular Nursing. 2017;16(SUPPL 1):S44.
6. Galema-Boers JMH, Steward K, Engelkes SR, Sijbrands EJ, Roeters Van Lennep JE. Real world experience of efficacy and safety using proprotein convertase subtilisin/kexin 9 (PCSK9) inhibition in patients with familial hypercholesterolemia. European Journal of Preventive Cardiology. 2017;24(1 Supplement 1):S136.
7. **Gao 2023**

Gao Y, Xu J, Cao S, Xu Y. A retrospective analysis of adherence in Chinese ischemic stroke and transient ischemic attack patients treated with PCSK9 monoclonal antibody for 6 months. European Stroke Journal. 2023;8(2 Supplement):558.

1. **Garcia-Pena 2023**

Garcia-Pena AA, Pineda-Posada M, Paez-Canro C, Cruz C, Samaca-Samaca D. Analysis of the evolocumab (Repatha) patient support program for patients with cardiovascular disease in Colombia. Clinica e Investigacion en Arteriosclerosis. 2023;35(6):280-9.

1. **Gargiulo 2024**
2. Gargiulo P, Basile C, Cesaro A, Marzano F, Buonocore D, Asile G, et al. Efficacy, safety, adherence and persistence of PCSK9 inhibitors in clinical practice: A single country, multicenter, observational study (AT-TARGET-IT). Atherosclerosis. 2023;366((Gargiulo, Basile, Buonocore, Asile, Abbate, Vicidomini, Paolillo, Spaccarotella, Perrone-Filardi) Department of Advanced Biomedical Sciences, University of Naples Federico II, Naples, Italy(Cesaro, Calabro) Department of Translational Medical Sciences, U):32-9.
3. Gargiulo P, Basile C, Galasso G, Bellino M, D'Elia D, Patti G, et al. Strike early-strike strong lipid-lowering strategy with PCSK9i in ACS patients. Real-world evidence from AT-TARGET-IT registry. European journal of preventive cardiology. 2024((Gargiulo, Basile, Marzano, Fontanarosa, Buonocore, Parlati, Nardi, Prastaro, Paolillo, Perrone-Filardi) Department of Advanced Biomedical Sciences, Federico II University, Naples, Italy(Galasso, Bellino, D'Elia) Department of Medicine, Surgery and Dentis).
4. Marzano F, Basile C, Paolillo S, Cesaro A, Buonocore D, Asile G, et al. Adherence and persistence of PCSK9 inhibitors in clinical practice: The real-world Italian experience (AT-TARGET-IT study). European Heart Journal, Supplement. 2022;24(Supplement K):K250.
5. Perrone-Filardi P, Basile C, Asile G, Abbate C, Catalano A, Merlini PA, et al. PCSK9 inhibitors: Effectiveness of treatment and changes in background lipid-lowering therapy in a real world Italian population. The AT-TARGET-IT study. European Respiratory Journal. 2022;60(Supplement 66):2378.
6. **Gayoso-Rey 2021**

Gayoso-Rey M, Diaz-Trastoy O, Romero-Ventosa EY, Garcia-Beloso N, Gonzalez-Freire L, Lorenzo-Lorenzo K, et al. Effectiveness, safety, and adherence to treatment of proprotein convertase subtilisin/kexin type 9 inhibitors in real practice. Clinical Therapeutics. 2021;43(4):e111-e21.

1. **Ghazi 2023**

Ghazi L, Jones J, Colantonio L, Poudel B, Wen Y, Wang Z, et al. Persistence and adherence to proprotein convertase subtilisin/kexin type 9 monoclonal antibodies and ezetimibe in real-world settings. Circulation. 2023;148(Supplement 1).

1. **Goicoechea 2022**
2. Goicoechea M, Alvarez V, Segarra A, Polaina M, Martin-Reyes G, Robles NR, et al. Lipid profile of patients treated with evolocumab in Spanish hospital nephrology units (RETOSS NEFRO). Nefrologia. 2022;42(3):301-10.
3. Goicoechea M, Alvarez V, Segarra A, Polaina M, Martin G, Robles-Perez NR, et al. Clinical profile of patients initiating evolocumab in Spanish nephrology units: A restrospective, observational study in clinical practice (retossnephro). Nephrology Dialysis Transplantation. 2020;35(SUPPL 3):iii566.
4. **Gragnano 2018**
5. Gragnano F, Natale F, Concilio C, Fimiani F, Cesaro A, Sperlongano S, et al. Adherence to proprotein convertase subtilisin/kexin 9 inhibitors in high cardiovascular risk patients: An Italian single-center experience. Journal of Cardiovascular Medicine. 2018;19(2):75-7.
6. Gragnano F, Concilio C, Cesaro A, Crisci M, Sperlongano S, Fimiani F, et al. Adherence to PCSK9 inhibitors in high cardiovascular risk patients in real-world setting: Results from a single-center experience and comparison with statin therapy. European Heart Journal. 2017;38(Supplement 1):314.
7. **Gupta 2023**
8. Gupta M, Wani RJ, Al Faraidy K, Bergeron J, Contreras E, Pena AAG, et al. Real-world insights into evolocumab use in patients with hyperlipidemia across five countries: Analysis from the ZERBINI study. Cardiology and Therapy. 2023;12(4):703-22.
9. Al Faraidy K, Akbar M, Shehri M, Aljarallah M, Hussein GA, Dashti R, et al. Multizonal observational study conducted by clinical practitioners on evolocumab use in subjects with hyperlipidemia in Saudi Arabia and Kuwait: Results from the ZERBINI study. PLoS ONE. 2023;18(1 January):e0278821.
10. Gupta M, Mancini GBJ, Wani RJ, Ahooja V, Bergeron J, Manjoo P, et al. Real-World insights into evolocumab use in patients with hyperlipidemia: Canadian analysis from the ZERBINI study. CJC Open. 2022;4(6):558-67.
11. Roncancio HM, Lugo-Pena JR, Garcia AA, Leal J, Hoyos CA, Beltran JA, et al. Multizonal observational study conducted by clinical practitioners on Repatha use in patients with hyperlipidemia (ZERBINI): Colombian results. Clinica e Investigacion en Arteriosclerosis. 2024;36(1):22-32.
12. **Gurgoze 2018**

Gurgoze MT, Muller-Hansma AHG, Schreuder MM, Galema-Boers AMH, Boersma E, Roeters van Lennep JE. Adverse events associated with PCSK9 inhibitors: A real-world experience. Clinical Pharmacology and Therapeutics. 2019;105(2):496-504.

1. **Han 2021**

Han J, Bilgrami S, Ross S, Broadhead H, Attar N. Real-world lipid lowering effects of proprotein convertase subtilisin/kexin type 9 (PCSK9) inhibitors: A single-centre study. International Journal of Cardiology. 2021;322((Han, Bilgrami, Ross, Attar) Department of Cardiology, Royal Lancaster Infirmary, Lancaster, United Kingdom(Broadhead) University of Lancaster, United Kingdom):240-4.

1. **Heintjes 2019**

Heintjes E, Hartgers ML, Bezemer I, Hovingh GK, Stroes ES, Beest FPV, et al. Real-world use of proprotein convertase subtilisin/kexin type 9 inhibitor (PCSK9-i) antibodies in the Netherlands. Atherosclerosis. 2019;287((Heintjes, Bezemer, Beest) PHARMO Institute for Drug Outcomes Research, N/a, Utrecht, Netherlands(Hartgers, Hovingh, Stroes) Amsterdam University Medical Centers, University of Amsterdam, Department of Vascular Medicine, Amsterdam, Netherlands(Kutikova, V):e199-e200.

1. **Hines 2018**
2. Hines DM, Rane P, Patel J, Harrison DJ, Wade RL. Treatment patterns and patient characteristics among early initiators of PCSK9 inhibitors. Vascular Health and Risk Management. 2018;14((Hines, Wade) Health Economics and Outcomes Research, IQVIA, Plymouth Meeting, PA, United States(Rane, Patel, Harrison) Global Health Economics, Amgen Inc, Thousand Oaks, CA, United States):409-18.
3. Hines D, Rane P, Patel J, Harrison D, Wade R. Persistence and adherence with proprotein convertase subtilisin/kexin type 9 inhibitors in clinical practice. Journal of the American College of Cardiology. 2017;69(11 Supplement 1):159.
4. Hines D, Rane P, Patel J, Harrison D, Wade R. Treatment patterns among early initiators of evolocumab and alirocumab. Journal of Managed Care and Specialty Pharmacy. 2017;23(3-A SUPPL.):S70.
5. **Iqbal 2022**

Iqbal S, Sabbour HM, Siddiqui MS, Tikriti AA, Santos RD, Buckley A. The first report of a real-world experience with a PCSK9 inhibitor in a large familial hyperlipidemia and very-high-risk Middle Eastern population. Clinical Therapeutics. 2022;44(10):1297-309.

1. **Iqbal 2024**

Iqbal S, Sabbour HM, Ashraf T, Santos RD, Buckley A. First report of inclisiran utilization for hypercholesterolemia treatment in real-world clinical settings in a Middle East population. Clinical Therapeutics. 2024;46(3):186-93.

1. **Kaufman 2019**

Kaufman TM, Warden BA, Minnier J, Miles JR, Duell PB, Purnell JQ, et al. Application of PCSK9 inhibitors in practice: Part 2: The patient experience. Circulation Research. 2019;124(1):32-7.

1. **Khatib 2022**

Khatib R, Khan M, Barrowcliff A, Ikongo E, Burton C, Mansfield M, et al. Innovative, centralised, multidisciplinary medicines optimisation clinic for PCSK9 inhibitors. Open Heart. 2022;9(1):e001931.

1. **Kim 2023**

Kim OM, Givens TK, Tang EG, Schimmer JJ, Ramsey T, Boyd K, et al. Real-world outcomes of proprotein convertase subtilisin kexin-9 inhibitor use. Journal of Cardiovascular Pharmacology. 2023;81(5):339-47.

1. **Klebs 2022**

Klebs S, Lecocq J, Mann C, Zingel R, Seshagiri D, Lahoz R. Characterization of inclisiran use in real world: Prescription data from Germany. Journal of Clinical Lipidology. 2022;16(3 Supplement):e73-e4.

1. **Knickelbine 2018**

Knickelbine T, Garberich R, White S, Oberembt S, Wills S, Miedema M, et al. PCSK9 inhibitors in patients with familial hypercholesterolemia and/or CVD: The impact of a systematic, team-based approach to prescription on rates of insurance approval and lipid lowering. Journal of the American College of Cardiology. 2018;71(11 Supplement 1).

1. **Koenig 2024**

Koenig W, Lorenz ES, Beier L, Gouni-Berthold I. Retrospective real-world analysis of adherence and persistence to lipid-lowering therapy in Germany. Clinical Research in Cardiology. 2024;113(6):812-21.

1. **Kohli 2017**
2. Kohli M, Patel K, MacMahon Z, Ramachandran R, Crook MA, Reynolds TM, et al. Pro-protein subtilisin kexin-9 (PCSK9) inhibition in practice: lipid clinic experience in 2 contrasting UK centres. International Journal of Clinical Practice. 2017;71(11):e13032.
3. Kohli M, McMahon Z, Ramachandran R, Crook MA, Reynolds TM, Wierzbicki AS. An audit of the effectiveness of PCSK9 inhibition in reducing cholesterol in patients attending lipid clinics. Atherosclerosis Supplements. 2017;28((Kohli, McMahon, Ramachandran, Crook, Wierzbicki) Dept Metabolic Medicine, Guy's and St Thomas' Hospitals, London, United Kingdom(Reynolds) Dept Chemical Pathology, Queen's Hospital, Burton-on-Trent, United Kingdom):e11.
4. **Lafratte 2023**

Lafratte C, Peasah SK, Huang Y, Hall D, Patel U, Good CB. Association of PCSK9 inhibitor initiation on statin adherence and discontinuation. Journal of the American Heart Association. 2023;12(18):e029707.

1. **Lahoz 2024**

Lahoz R, Seshagiri D, Electricwala B, Achouba A, Ding Y, Heo JH, et al. Clinical characteristics and treatment patterns in patients with atherosclerotic cardiovascular disease with hypercholesterolemia: a retrospective analysis of a large US real-world database cohort. Current Medical Research and Opinion. 2024;40(1):15-25.

1. **Leitner 2020**

Leitner DR, Toplak H, Kedenko L, Steinmaurer T, Graff V, Metzner T, et al. Efficacy and tolerability of alirocumab in Austrian clinical practice-results of the non-interventional PEARL-AT study. Current Medical Research and Opinion. 2020((Leitner, Toplak) Division of Endocrinology and Diabetology, Department of Internal Medicine, Medical University Graz, Graz, Austria(Kedenko) First Department of Internal Medicine, Paracelsus Medical University/Salzburger Landeskliniken, Salzburg, Austria):1-7.

1. **Lorena Martin Polo 2019**

Lorena Martin Polo L, Marco Clement I, Dalmau Gonzalez-Gallarza R, Merino Argos C, Rivas Perez A, Velez Salas A, et al. PCSK9 inhibitors use in clinical practice: A Tertiary Hospital experience. European Journal of Preventive Cardiology. 2019;26(Supplement 1):S109.

1. **Maciejko 2019**

Maciejko JJ, Jamoua R, Anne P. Assessment and management of patients with hyperlipidemia referred for initiation of PCSK9 inhibitor therapy: A lipid clinic experience. American Journal of Cardiovascular Drugs. 2019;19(6):553-9.

1. **Merino Martin 2018**

Merino Martin V, Ortega-Garcia MP, Blasco-Segura P, Sanfeliu Garcia J, Lopez Carrasco A, Del Rio San Cristobal R, et al. Effectiveness and safety of monoclonal antibody PCSK9 inhibitors. European Journal of Hospital Pharmacy. 2018;25(Supplement 1):A57.

1. **Mongiello 2023**

Mongiello P, Petti R, Ciaccia A, Morgese MG, Lombardi R. Analysis of adherence to anti-PCSK9 antibody therapy among patients from Italy. Cardiovascular and Hematological Disorders - Drug Targets. 2023;23(2):111-21.

1. **Mulder 2023**

Mulder JWCM, Galema-Boers AMH, Roeters van Lennep JE. First clinical experiences with inclisiran in a real-world setting. Journal of Clinical Lipidology. 2023;17(6):818-27.

1. **Muntner 2024**

Muntner P, Ghazi L, Jones J, Dhalwani N, Poudel B, Wen Y, et al. Persistence and adherence to PCSK9 inhibitor monoclonal antibodies versus ezetimibe in real-world settings. Advances in Therapy. 2024;41(6):2399-413.

1. **Nanchen 2022**
2. Nanchen D, Carballo D, Bilz S, Rickli H, Koskinas KC, Mach F, et al. Effectiveness, adherence, and safety of evolocumab in a Swiss multicenter prospective observational study. Advances in Therapy. 2022;39(1):504-17.
3. Nanchen D, Carballo D, Reichert N, Sudano I. Effectiveness, adherence and safety of evolocumab in a Swiss multicenter prospective observational study. European Heart Journal. 2021;42(SUPPL 1):2582.
4. Nanchen D, Wilhelm M, Carballo D, Reichert N, Sudano I. Use of evolocumab in patients at high cardiovascular risk: the Swiss multicenter prospective observational ECARA study. Kardiovaskulare Medizin. 2020;23(SUPPL 28):46S-7S.
5. **Naoum 2024**

Naoum I, Saliba W, Aker A, Zafrir B. Lipid-lowering therapy with inclisiran in the real-world setting: Initial data from a national health care service. Journal of Clinical Lipidology. 2024((Naoum, Aker, Zafrir) Department of Cardiology, Lady Davis Carmel Medical Center, Haifa, Israel(Saliba) Community Medicine and Epidemiology, Lady Davis Carmel Medical Center, Haifa, Israel(Saliba, Zafrir) Ruth and Bruce Rappaport Faculty of Medicine, Tech).

1. **Niu 2023**

Niu C, Parlapalli A, Neenan J, Ma X, Osei-Wusu A, Park J, et al. Six-month adherence among early inclisiran initiators vs. Anti-PCSK9 mAbs users: A retrospective analysis of US claims databases. Circulation. 2023;148(Supplement 1).

1. **Oren 2019**

Oren O, Kludtke EL, Kopecky SL. Characteristics and outcomes of patients treated with proprotein convertase subtilisin/kexin type 9 inhibitors (the Mayo Clinic experience). American Journal of Cardiology. 2019;124(11):1669-73.

1. **Pandey 2018**

Pandey AK, Bajaj H, Garg V, Pandey AS, Verma S. Efficacy and barriers to evolocumab therapy in real-world, high risk secondary prevention patients not at LDL targets. Circulation. 2017;136(Supplement 1).

1. **Parhofer 2019**
2. Parhofer KG, von Stritzky B, Pietschmann N, Dorn C, Paar WD. PEARL: A non-interventional study of real-world alirocumab use in German clinical practice. Drugs - Real World Outcomes. 2019;6(3):115-23.
3. Parhofer KG, Von Stritzky B, Paar WD. PEARL, a non-interventional study on real-world use of alirocumab in German clinical practice: Final study and cardiovascular subgroup data. European Heart Journal. 2018;39(Supplement 1):1104.
4. Von Stritzky B, Paar WD, Parhofer KG. PEARL-alirocumab real-world use in German clinical practice. Vasa - European Journal of Vascular Medicine. 2018;47(Supplement 99):15.
5. **Piccinni 2019**
6. Piccinni C, Antonazzo IC, Maggioni AP, Pedrini A, Calabria S, Ronconi G, et al. PCSK9 inhibitors' new users: Analysis of prescription patterns and patients' characteristics from an Italian real-world study. Clinical Drug Investigation. 2020;40(2):173-81.
7. Piccinni C, Antonazzo IC, Maggioni AP, Pedrini A, Calabria S, Ronconi G, et al. Utilization of PCSK9 inhibitors in an Italian region during the first year of access to national healthcare service reimbursement. Pharmacoepidemiology and Drug Safety. 2019;28(Supplement 2):473.
8. **Popadic 2024**
9. Popadic L, Ma X, Ali Y, Kumparatana P, Wei Y, McElligott S, et al. Treatment patterns among early inclisiran vs anti-PCSK9 mAbs users: A retrospective analysis of US claims databases. Journal of Clinical Lipidology. 2024;18(4 Supplement):e515-e6.
10. Lan N. Treatment patterns among early inclisiran vs anti-PCSK9 mAbs users: A retrospective analysis of US claims databases. Heart Lung and Circulation. 2024;33(Supplement 4):S542.
11. **Rallidis 2020**

Rallidis LS, Skoumas I, Liberopoulos EN, Vlachopoulos C, Kiouri E, Koutagiar I, et al. PCSK9 inhibitors in clinical practice: Novel directions and new experiences. Hellenic Journal of Cardiology. 2020;61(4):241-5.

1. **Ray 2023**
2. Ray KK, Bruckert E, Peronne-Filardi P, Ebenbichler C, Vogt A, Bridges I, et al. Long-term persistence with evolocumab treatment and sustained reductions in LDL-cholesterol levels over 30 months: Final results from the European observational HEYMANS study. Atherosclerosis. 2023;366((Ray) Imperial Centre for Cardiovascular Disease Prevention and Imperial Clinical Trials Unit, Imperial College London, London, United Kingdom(Bruckert) Department of Medicine, Sorbonne University of Paris, Paris, France(Peronne-Filardi) Department of Adv):14-21.
3. Blaha V, Margoczy R, Petrov I, Postadzhiyan A, Raslova K, Rosolova H, et al. Evolocumab is initiated in Central and Eastern Europe at much higher LDL-C levels than recommended in guidelines: Results from the observational HEYMANS study. Journal of Cardiovascular Pharmacology and Therapeutics. 2023;28((Blaha) 3rd Department of Internal Medicine - Metabolic Care and Gerontology, University Hospital Hradec Kralove and Charles University, Hradec Kralove, Czechia(Margoczy) Department of General Cardiology, Middle Slovak Institute of Cardiovascular Diseases).
4. Blaha V, Margoczy R, Petrov I, Postadzhiyan A, Raslova K, Rosolova H, et al. Evolocumab is initiated in Central and Eastern Europe at much higher LDL-C levels than recommended in guidelines: Results from the observational HEYMANS study. European cardiology. 2023;18(101574780):e36.
5. Blanco Echevarria A, Garcia Diaz JDD, Caixas A, Plana Gil N, Rico Corral MA, Bridges I, et al. Long-term treatment persistence and maintained reduction of LDL-cholesterol levels with evolocumab over 30 months: Results from the Spanish cohort of the European prospective HEYMANS study. Clinica e Investigacion en Arteriosclerosis. 2023;35(6):263-71.
6. Ebenbichler C, Drexel H, Hanusch U, Toplak H, Dhalwani NN, Bridges I, et al. Evolocumab effectiveness in the real-world setting: Austrian data from the pan-European observational HEYMANS study. Wiener Klinische Wochenschrift. 2024;136(3-4):77-86.
7. Filardi PP, Calabro P, Galasso G, Paloscia L, Ignone G, Bridges I, et al. Evolocumab use in clinical practice in Italy: Final data of the HEYMANS study. European Heart Journal, Supplement. 2022;24(Supplement K):K54.
8. Lehrke M, Vogt A, Schettler V, Girndt M, Fraass U, Tabbert-Zitzler A, et al. Evolocumab-based LDL-C management in high and very high cardiovascular risk patients in German clinical practice: The HEYMANS study. Advances in Therapy. 2024;41(3):1184-200.
9. Ray K, Bruckert E, Annemans L, Van Hout B, Schoonen M, Bridges I. Characteristics of patients prescribed evolocumab in Europe. Does clinical use match clinical guidelines? European Heart Journal. 2018;39(Supplement 1):343.
10. Ray K, Perrone-Filardi P, Ebenbichler C, Vogt A, Bridges I, Sibartie M, et al. Safety of very low LDL-C levels with evolocumab: An analysis from the pan-European observational HEYMANS study. Atherosclerosis. 2023;379(Supplement 1):S43.
11. Ray KK, Bruckert E, Filardi P, Ebenbichler C, Vogt A, Bridges I, et al. Evolocumab use in Europe: Clinical guidelines vs. reimbursement thresholds-results from the HEYMANS study. European Heart Journal. 2021;42(SUPPL 1):2940.
12. Ray KK, Bruckert E, Perrone-Filardi P, Ebenbichler C, Vogt A, Bridges I, et al. Final results from the pan-European observational heymans study suggest a mismatch between guidelines and PCSK9i reimbursement criteria. Atherosclerosis. 2022;355((Ray) Department Of Primary Care And Public Health And Ictu-global, Imperial College London, London, United Kingdom(Bruckert) Department Of Medicine, University of Paris, Paris, France(Perrone-Filardi) Department Of Advanced Biomedical Sciences, Universit):14.
13. Ray KK, Dhalwani N, Sibartie M, Bridges I, Ebenbichler C, Perrone-Filardi P, et al. Low-density lipoprotein cholesterol levels exceed the recommended European threshold for PCSK9i initiation: lessons from the HEYMANS study. European Heart Journal - Quality of Care and Clinical Outcomes. 2022;8(4):447-60.
14. Ray KK, Perrone-Filardi P, Ebenbichler C, Vogt A, Bridges I, Sibartie M, et al. Evolocumab treatment is associated with early and sustained reductions in low-density cholesterol (LDL-C) over 30 months: Final results from the pan-European observational HEYMANS registry. European Respiratory Journal. 2022;60(Supplement 66):2667.
15. Ray KK, Perrone-Filardi P, Ebenbichler C, Vogt A, Bridges I, Sibartie M, et al. High long-term persistence to evolocumab treatment regimens in European clinical practice: Analysis of the HEYMANS registry. European Respiratory Journal. 2022;60(Supplement 66):2666.
16. Ray KK, Schoonen M, Annemans L, Van Hout BA, Sibartie M, Bridges I, et al. Effectiveness of evolocumab for patients with familial hypercholesteraemia (FH) in European clinical practice. European Heart Journal. 2019;40(Supplement 1):263.
17. Sibartie M, Dhalwani N, Bridges I, Ebenbichler C, Filardi PP, Vogt A, et al. Evolocumab use and LDL-C lowering in a cohort of European patients with familial hypercholesterolemia (FH) - results from the HEYMANS study. Atherosclerosis. 2021;331((Sibartie) Amgen (Europe) GmbH, Global Medical, Rotkreuz, Switzerland(Dhalwani) Amgen Ltd, Center For Observational Research, Uxbridge, United Kingdom(Bridges) Amgen Ltd, International Biostatistics, Uxbridge, United Kingdom(Ebenbichler) Medizinische Univ):e253.
18. Sudano I, Krahenbuhl S, Anstett A, Dhalwani N, Bridges I, Sibartie M, et al. Evolocumab use in clinical practice in Switzerland: Final data of the HEYMANS study. Swiss Medical Weekly. 2022;152(Supplement 260):71S.
19. Sudano I, Krahenbuhl S, Mach F, Anstett A, Dhalwani N, Bridges I, et al. Evolocumab use in clinical practice in Switzerland: Final data of the observational HEYMANS cohort study. Therapeutic Advances in Cardiovascular Disease. 2024;18((Sudano) Department of Cardiology, University Hospital Zurich, University Heart Center, University of Zurich, Ramistrasse 100, Zurich 8091, Switzerland(Krahenbuhl) Department of Clinical Pharmacology, University Hospital Basel, Basel, Switzerland(Mach) De).
20. Vlachopoulos C, Massia D, Kochiadakis G, Kolovou G, Patsilinakos S, Bridges I, et al. Evolocumab use in Greece is associated with early and sustainable reductions in low-density cholesterol (LDL-C) and high persistence to therapy: Results from the Greek cohort analysis of the observational HEYMANS study. Hellenic Journal of Cardiology. 2023;74((Vlachopoulos) 1st Department of Cardiology, Medical School, National and Kapodistrian, University of Athens, Hippokration Hospital, Athens, Greece(Massia) Amgen Hellas, Athens, Greece(Kochiadakis) Cardiology Department, Heraklion University Hospital, Cre):74-6.
21. **Reynolds 2019**

Reynolds VW, Chinn ME, Jolly JA, Kelley TN, Peter ME, Choi L, et al. Integrated specialty pharmacy yields high PCSK9 inhibitor access and initiation rates. Journal of Clinical Lipidology. 2019;13(2):254-64.

1. **Rodriguez 2020**

Rodriguez A, Lopez C, Gonzalez-Colominas E, Luque S, Recasens L, Pedro-Botet J, et al. Long term efficacy, safety and adherence to alirocumab in patients with dyslipidaemia from a tertiary hospital cohort. European Journal of Hospital Pharmacy. 2020;27(SUPPL 1):A57-A8.

1. **Rymer 2020**

Rymer JA, Mues KE, Monda KL, Bratton EW, Wirtz HS, Okerson T, et al. Use of low-density lipoprotein-lowering therapies before and after PCSK9 inhibitor initiation. Journal of the American Heart Association. 2020;9(9):e014347.

1. **Saborowski 2018**

Saborowski M, Dolle M, Manns MP, Leitolf H, Zender S. Lipid-lowering therapy with pcsk9-inhibitors in the management of cardiovascular high-risk patients: Effectiveness, therapy adherence and safety in a real world cohort. Cardiology Journal. 2018;25(1):32-41.

1. **Sammour 2021**
2. Sammour Y, Dezorzi C, Austin BA, Borkon AM, Everley MP, Fendler TJ, et al. PCSK9 inhibitors in heart transplant patients: Safety, efficacy, and angiographic correlates. Journal of Cardiac Failure. 2021;27(7):812-5.
3. Sammour Y, Austin BA, Borkon M, Everley MP, Fendler TJ, Khumri TM, et al. Safety and effectiveness of pcsk9 inhibitors in orthotopic heart transplant patients. Journal of Cardiac Failure. 2020;26(10 Supplement):S22.
4. **Sathiyakumar 2017**

Sathiyakumar V, Martin SS, Jones S, Quinn J, Green R, Lesko A, et al. Real-world PCSK9i experience: The importance of a multidisciplinary approach. Journal of Clinical Lipidology. 2017;11(3):803.

1. **Sbrana 2020**

Sbrana F, Dal Pino B, Bigazzi F, Ripoli A, Volpi E, Lo Surdo G, et al. A large Italian cohort on PCSK9-inhibitors: A single center experience. European Heart Journal, Supplement. 2020;22(SUPPL G):G73-G4.

1. **Sheng 2024**

Sheng F, Wang AY, Miyawaki K, Tsuchiya T, Osada N, Miller R, et al. Real-world clinical profile of patients prescribed evolocumab in Japan. Circulation journal : official journal of the Japanese Circulation Society. 2024((Sheng, Miyawaki, Tsuchiya, Osada) Medical Affairs(Wang) Center for Observational Research, Australia(Miller, Fu) Syneos Health(Okamura) Department of Preventive Medicine and Public Health, Keio University School of Medicine).

1. **Smith 2019**

Smith AB, Johnson D, Sarkar K, Connelly A, Karalis D. Patient characteristics and prescribing patterns for patients approved and denied PCSK9 inhibitors in real-world practice. Journal of Clinical Lipidology. 2019;13(3):e38.

1. **Snel 2022**

Snel M, Descamps O. Long-term safety and effectiveness of alirocumab and evolocumab in familial hypercholesterolemia (FH) in Belgium. Acta Clinica Belgica. 2022;77(Supplement 2):22-3.

1. **Starner 2016**

Starner C, Giguere J, Gunderson B, Gleason P, Johnson S. PCSK9i utilization, cost, utilization management impact, and discontinuation rate among 13 million commercially insured Americans. Journal of Managed Care and Specialty Pharmacy. 2016;22(4-A SUPPL.):S55.

1. **Stoekenbroek 2017**

Stoekenbroek RM, Hartgers ML, Rutte R, de Wijer DD, Stroes ESG, Hovingh GK. PCSK9 inhibitors in clinical practice: Delivering on the promise? Atherosclerosis. 2018;270((Stoekenbroek, Hartgers, Rutte, de Wijer, Stroes, Hovingh) Department of Vascular Medicine, Academic Medical Center, Amsterdam 1100DD, Netherlands):205-10.

1. **Stummer 2023**

Stummer A, Ristl R, Kogler B, Muskovich M, Kossmeier M, Stulnig TM. Patient adherence to fully reimbursed proprotein convertase subtilisin/kexin type 9 inhibitor (PCSK9i) treatment. Wiener Klinische Wochenschrift. 2023;135(13-14):375-82.

1. **Svensson 2024**
2. Svensson MK, James S, Ravn-Fischer A, Villa G, Schalin L, Cars T, et al. A retrospective nationwide analysis of evolocumab use in Sweden and its effect on low-density lipoprotein cholesterol levels. Upsala journal of medical sciences. 2024;129((Svensson, James, Hagstrom) Department of Medical Sciences, Uppsala University, Uppsala, Sweden(Svensson, James, Hagstrom) Uppsala Clinical Research Centre, Uppsala, Sweden(Ravn-Fischer) Department of Cardiology, Sahlgrenska University Hospital, Gothenbur).
3. Svensson MK, James S, Ravn-Fischer A, Pantev E, Villa G, Schalin L, et al. Low-density lipoprotein cholesterol reduction with evolocumab and its use in clinical practice: Evidence from Swedish national register data. European Respiratory Journal. 2022;60(Supplement 66):2680.
4. **Tai 2018**

Tai MH, Shepherd J, Bailey H, Williams N, Hatz M, Campos Tapias I, et al. Real-world treatment patterns of PCSK9 inhibitors among patients with dyslipidemia in Germany, Spain, and the United Kingdom. Current Medical Research and Opinion. 2019;35(5):829-35.

1. **Vicente-Valor 2021**

Vicente-Valor J, Garcia-Gonzalez X, Ibanez-Garcia S, Duran-Garcia ME, de Lorenzo-Pinto A, Rodriguez-Gonzalez C, et al. PCSK9 inhibitors revisited: Effectiveness and safety of PCSK9 inhibitors in a real-life Spanish cohort. Biomedicine and Pharmacotherapy. 2022;146((Vicente-Valor, Garcia-Gonzalez, Ibanez-Garcia, Duran-Garcia, de Lorenzo-Pinto, Rodriguez-Gonzalez, Herranz-Alonso, Sanjurjo-Saez) Pharmacy Department, Hospital General Universitario Gregorio Maranon, Instituto de Investigacion Sanitaria Gregorio Maranon):112519.

1. **Waldmann 2020**
2. Waldmann E, Altenhofer J, Henze K, Parhofer KG. German lipid clinic experience with adherence to PCSK-9-inhibitor therapy. Atherosclerosis. 2020;315((Waldmann, Altenhofer, Henze, Parhofer) Marchioninistr. 15, Internal Medicine Iv, Metabolism And Endocrinology, Munich, Germany):e54.
3. Waldmann E, Altenhofer J, Henze K, Parhofer KG. PCSK9-inhibition in routine clinical practice: Real world data from Germany. Atherosclerosis. 2019;287((Waldmann, Altenhofer, Henze, Parhofer) Hospital of the University of Munich, Department of Internal Medicine IV, Munich, Germany):e38.
4. **Warden 2021**

Warden BA, Purnell JQ, Duell PB, Craigan C, Osborn D, Cabot E, et al. Real-world utilization of pharmacotherapy with new evidence-based cardiovascular indications in an academic preventive cardiology practice. American journal of preventive cardiology. 2021;5(101769122):100144.

1. **Weber 2019**

Weber BR, Collins M, Dajani K. PCSK9 compliance, a single center experience. Clinical Cardiology. 2019;42(Supplement 2):S46-S7.

1. **Wiener 2020**

Wiener C, Levintow SN, Orroth KK, Gill K, Mullard A, McGrath LJ, et al. Real world treatment patterns following the initiation of proprotein convertase subtilisin/kexin type 9 antibody inhibitors among adults in the United States, 2015-2019. Pharmacoepidemiology and Drug Safety. 2020;29(SUPPL 3):55-6.

1. **Wong 2023**

Wong WB, Seetasith A, Hung A, Zullig LL. Impact of list price changes on out-of-pocket costs and adherence in four high-rebate specialty drugs. PLoS ONE. 2023;18(1 January):e0280570.

1. **Xiao 2023**

Xiao F, Gao X, Wang X, Li X, Li R, Yan Q, et al. Real-world adherence to anti-PCSK9 monoclonal antibody (mAb) and its impact on economic burden of cardiovascular diseases in patients with hypercholesterolemia: A retrospective cohort study in China. Value in Health. 2023;26(12 Supplement):S491.

1. **Zafrir 2018**

Zafrir B, Jubran A. Lipid-lowering therapy with PCSK9-inhibitors in the real-world setting: Two-year experience of a regional lipid clinic. Cardiovascular therapeutics. 2018;36(5):e12439.

1. **Zafrir 2020**
2. Zafrir B, Egbaria A, Stein N, Elis A, Saliba W. PCSK9 inhibition in clinical practice: Treatment patterns and attainment of lipid goals in a large health maintenance organization. Journal of Clinical Lipidology. 2021;15(1):202-11.e2.
3. Zafrir B, Hislop E, Volis I, Saliba W. A safety and clinical efficacy analysis of PCSK9 monoclonal antibodies in patients with markedly elevated creatine phosphokinase levels. European Heart Journal. 2021;42(SUPPL 1):2938.
4. Volis I, Hislop E, Saliba W, Zafrir B. A safety and clinical efficacy analysis of PCSK9 monoclonal antibodies in patients with markedly elevated creatine phosphokinase levels. American Journal of Blood Research. 2021;11(4):399-404.
